# Brain network pathophysiology in dystonia

**DOI:** 10.3389/dyst.2025.15446

**Published:** 2026-01-30

**Authors:** David A. Peterson, Myungjoo Kim, Robert Chen, David Eidelberg, Cecile Gallea, Andreas G. Horn, Stephane Lehericy, Anthony R. McIntosh, Joel S. Perlmutter, Anna Sadnicka, Terrence D. Sanger, Emiliano Santarnecchi, Philip A. Starr, Jan K. Teller, Mark Hallett, Kristina Simonyan

**Affiliations:** 1 Institute for Neural Computation, University of California, San Diego, La Jolla, CA, United States,; 2 Computational Neurobiology Laboratory, Salk Institute for Biological Studies, La Jolla, CA,United States,; 3 Division of Neurology, Department of Medicine, University of Toronto and Krembil Research Institute, University Health Network, ON, Canada,; 4 Center for Neurosciences, The Feinstein Institutes for Medical Research, Manhasset, NY, United States,; 5 Paris Brain Institute (ICM), Team Movement Investigations and Therapeutics, CNRS (UMR 7225)/INSERM (U-1127)/Sorbonne-Université, Paris, France,; 6 Department Neurology, Brigham and Women’s Hospital, Boston, MA, United States,; 7 Network Stimulation Institute, Department of Stereotactic and Functional Neurosurgery, University Hospital Cologne, Cologne, Germany,; 8 Center for Brain Circuit Therapeutics Department of Neurology, Brigham and Women’s Hospital, Harvard Medical School, Boston, MA, United States,; 9 MGH Neurosurgery and Center for Neurotechnology and Neurorecovery (CNTR) at MGH Neurology Massachusetts General Hospital, Harvard Medical School, Boston, MA, United States,; 10 Paris Brain Institute (ICM), Centre for NeuroImaging Research (CENIR), Department Neuroradiology, La Pitie-Salpetriere Hospital (APHP), Sorbonne-Université, Paris, France,; 11 Department Biomedical Physiology and Kinesiology, Institute for Neuroscience and Neurotechnology, Simon Fraser University, Burnaby, BC, Canada,; 12 Neurology, Radiology, Neuroscience, Physical Therapy, and Occupational Therapy, Washington University School of Medicine, St. Louis, MO, United States,; 13 Gatsby Computational Neuroscience Unit, University College London, London, United Kingdom,; 14 Department of Clinical and Movement Neurosciences, University College London, London, United Kingdom,; 15 Department Neurology, Children’s Hospital of Orange County, University of California, Irvine, CA, United States,; 16 Department EECS, Children’s Hospital of Orange County, University of California, Irvine, United States,; 17 Department Radiology, Massachusetts General Hospital, Boston, MA, United States,; 18 Department of Neurological Surgery, University of California, San Francisco, CA, United States,; 19 Dystonia Medical Research Foundation, Chicago, IL, United States,; 20 National Institute of Neurological Disorders and Stroke, NIH, Bethesda, MD, United States,; 21 Department Otolaryngology - Head and Neck Surgery, Harvard Medical School and Massachusetts Eye and Ear, Boston, MA, United States,; 22 Department Neurology, Massachusetts General Hospital, Boston, MA, United States

**Keywords:** brain networks, DBS, dystonia, MRI, PET

## Abstract

Dystonia is increasingly recognized as a disorder of brain networks. This review integrates multimodal evidence from human studies to characterize the network-level pathophysiology of dystonia. Structural MRI studies using voxel-based morphometry and diffusion imaging reveal alterations in gray matter volume and white matter connectivity across the sensorimotor cortex, basal ganglia, cerebellum, and thalamus. Functional imaging modalities, including PET, fMRI, EEG, MEG, and fNIRS, demonstrate aberrant activity and connectivity in cortico-striato-pallido-thalamocortical and cerebello-thalamocortical loops. Invasive electrophysiological recordings from deep brain stimulation (DBS) provide high-resolution insights into abnormal oscillatory activity and effective connectivity within these circuits. Non-invasive brain stimulation (NIBS) techniques such as TMS, TES, and TUS provide a means of actively interrogating those networks through transient perturbation. They also provide an avenue for personalized neuromodulation. Computational models, including The Virtual Brain platform, enable integration of multimodal data to simulate dynamic network behavior. Across focal, generalized, and genetic forms of dystonia, shared patterns of network dysfunction are observed, though phenotypic and genotypic subtypes exhibit distinct topographies and circuit-level alterations. These findings underscore the importance of network dysfunction underlying dystonia. This network perspective informs the development of more targeted and individualized diagnostic and therapeutic approaches, including circuit-guided neuromodulation and closed-loop brain stimulation. Advancing multimodal and integrative methodologies will be essential to unraveling the complex dynamics underlying dystonia and translating mechanistic insights into precision interventions.

## Introduction

The current view of how the brain functions is that of networks. Networks play a key role in human brain function. The original movement away from a phrenology view came from the German school at the end of the 19th century and the beginning of the 20th century with Broca’s and Wernicke’s studies of language and aphasia and Liepmann’s studies of movement and apraxia [1]. They stressed the importance of information flowing from one part of the brain to another underlying function. This view was attacked by the British school, including persons such as Head, calling these neurologists “diagram makers” and adopting a more gestalt view of brain function. Geschwind [2, 3] in a game-changing two-part paper in Brain in 1965, brought back the idea of the importance of brain connections to understand normal function and pathophysiology, and, stimulated by advances in MRI and EEG [4], networks have become the predominant model once again.

Increasing data demonstrate that normal movement depends on network function. Similarly, the pathophysiology of disordered movements reflects dysfunction at the network level [5]. This approach provides a basis for understanding the pathophysiology of dystonia.

Dystonia is defined as a “movement disorder characterized by sustained or intermittent abnormal movements, postures, or both” [6]. There are many syndromes of dystonia (axis 1) and many etiologies (axis 2). The pathophysiology of the movement disorder, however, appears to have similarities across this wide spectrum. While it is always optimal to treat the etiology of a disorder since that might eradicate it completely, when this is not possible, it is still often achievable to treat the symptoms. Understanding the network dysfunction, therefore, is not only useful in improving general knowledge about dystonia but also helpful for development of symptomatic treatment. Indeed, this is already clearly the case, since dystonia can be responsive to DBS whose mechanism of action includes brain circuit modification [7].

Studies of the pathophysiology of dystonia over the last several decades have revealed some fundamental abnormalities [8, 9]. The notion that dystonia is a network disorder dates back to at least the 1990s [10, 11] and continues to the current decade [12]. The motor network in particular has been suggested [13], and motor dysfunction in dystonia is often manifest as co-contraction of agonist and antagonist muscles [14]. There is also a loss of reciprocal inhibition at multiple levels, including in the spinal cord [15], in the brainstem in the form of enhanced blink reflex recovery [16], and in the motor cortex [17, 18]. One specific type of inhibition lost is surround inhibition, which predisposes to overflow movement and loss of selective motor control. In addition to overt abnormalities in the motor system, subtle abnormalities also affect the sensory system, including abnormal blood flow responses to vibratory stimulation [19, 20]. There are also abnormalities of brain plasticity with slow motor learning, some types of exaggerated plasticity, and loss of homeostatic plasticity [9]. These various physiological abnormalities, presumably resulting from brain miswiring and/or dysfunction of neurotransmitters such as GABA and dopamine and arising from genetic and environmental factors [21], are likely associated with brain network dysfunction.

This review focuses on multimodal evidence in humans of brain network dysfunction and how it might be ameliorated. Other recent reviews also cover network dysfunction in dystonia, though they exclude genetic etiologies and include rodent models [22] or focus on how DBS has provided insights into the brain networks and physiological mechanisms that underlie motor control, covering not only dystonia but also Parkinson’s disease with substantial attention to animal models [23].

## Organizational overview

A multitude of methods exist for studying brain networks in dystonia. In this review, we have organized them into four main categories: non-invasive brain imaging, invasive brain recordings associated with DBS, non-invasive brain stimulation, and models of brain network dysfunction.

The first part of the review summarizes results from non-invasive brain imaging, the primary measures of network activity currently available for use in humans. The imaging modalities include 1) computed tomography (e.g., for lesions); 2) structural MRI, involving information about grey matter structures from voxel-based morphometry and white matter pathways from diffusion MRI; 3) functional near-infrared spectroscopy (fNIRS), 4) positron emission tomography (PET), including metabolic patterns and the functions of neurotransmitters such as dopamine, GABA, and acetylcholine (Ach); and 5) functional MRI (fMRI), including both resting-state and task-related conditions; and 6) EEG.

The second part of the review summarizes results from invasive brain recordings associated with DBS. Compared to brain imaging methods, recordings during and after DBS surgery enable measures of brain activity with much higher spatial and temporal precision. The temporal precision enables analyses of stimulus-evoked responses and pathological synchronized oscillations hypothesized to play a role in the network pathophysiology. However, these recordings are generally limited to only those locations in the brain that are clinically indicated. Nevertheless, combining imaging enables a broader assay of network effects, and there is increasing use of recordings made simultaneously in multiple DBS targets.

The third part of the review summarizes results from non-invasive brain stimulation (NIBS). These include many applications of TMS and TES that have been explicitly designed to normalize the hallmarks of dystonia pathophysiology by decreasing excitation, increasing inhibition, and modulating abnormal plasticity. Another NIBS method more recently explored in dystonia is transcranial ultrasound stimulation (TUS). All the NIBS modalities could be optimized for each patient by leveraging the various recording modalities, and in some cases (e.g., with EEG, TMS, and TES), this can be done on-line in a closed loop.

The fourth part of the review highlights models for brain network dysfunction in dystonia. Historically, brain network models were constrained in their level of detail and breadth of scope by limitations of computational resources. Continuing advances in computing technologies have dramatically expanded those boundaries, as evidenced, for example, by recent developments of The Virtual Brain (TVB), an informatics platform to simulate whole brain dynamics. Although it is designed to work at the gross level of mean-field dynamics, this spatial level of abstraction is a good match for a large body of prior and ongoing experimental work with brain imaging measures.

The fifth part of the review outlines future directions for applied research into network pathophysiology of dystonia. It points out methodological and analytic standards that could strengthen interpretation of future imaging studies, how advances in DBS technology can provide novel clues about network pathophysiology, and that aspects of longitudinal and developmental dynamics on the dystonia network remain understudied. It also notes how motor behavior can provide important constraints on circuit models of the dysfunction. For example, overtrained movement patterns are thought to be a causal factor in many task-specific dystonias, and conversely properly designed physiotherapy interventions should be able to modulate the dystonic network toward normalized function. The relationships between genotype and phenotype–especially as both axes become better understood and characterized objectively–should also provide a helpful framework for understanding dystonia network pathophysiology. Finally, two specific phenotypic aspects of dystonia are highlighted as meriting more future investigation: tremor in dystonia and functional dystonia. Collectively all these research directions will help maximize what we can learn about the network pathophysiology of dystonia.

Woven throughout all parts of the review are a wide array of analytic methods, a variety of tasks, differential network findings for various dystonia subtypes defined phenomenologically and genetically, and the influence of treatments, including not only botulinum toxin (BoNT) but also DBS and TMS. Future efforts to synthesize these multiple approaches to understanding brain network dysfunction will accelerate progress toward new treatments that directly target the neural network basis of dystonia.

## Non-invasive brain imaging

In routine clinical imaging, most dystonia patients exhibit no overt abnormalities [13]. Regional and network changes are generally subtle and more functional than structural: one may show abnormal function despite structurally normal scans [24]. Functional disturbances often manifest as abnormal network interactions involving basal ganglia, cerebellum, thalamus, and cortex [25]. Bhatia et al. [26] identified lesions associated with dystonia on CT and MRI; their heterogeneous locations suggested that their involvement could be accounted for with dysfunction of a network. Focal lesions may not appear on CT or MRI yet can be evident on functional imaging with PET [27].

### Lesions

In dystonia, lesion-based studies most often implicate the basal ganglia and thalamus [26, 28]. Pediatric lesion-induced dystonias - including from hypoxia, kernicterus, and stroke -implicate two basal ganglia nuclei in particular: the putamen and globus pallidus (GP) [29]. These nuclei participate in the “somato-cognitive-action network” (SCAN) that involves M1 and plays a role in motor integration [30] and the “cingulo-opercular/action-mode network” (CON/AMN) that includes the dorsal anterior cingulate and anterior insula [31], indicating that basal-ganglia injury can influence higher-order motor planning as well as execution. Cortical lesions also contribute: in BSP involving both idiopathic and acquired forms, meta-analytic connectivity modeling revealed bilateral SMA abnormalities [32]. In lesion-based CD [33], affected sites were functionally connected within a network encompassing the cerebellum, GP, striatum, midbrain, thalamus, and somatosensory cortex–a pattern likewise observed in isolated CD.

### Voxel-based morphometry (VBM) and diffusion MRI

VBM quantifies local grey and white matter concentrations across the brain based on structural MRI. Diffusion MRI (dMRI) assesses water diffusion to infer the integrity and connectivity of white matter tracts linking distant regions. Together, these methods enable whole-brain assessment of structural networks, and diffusion-tractography studies have revealed differences in pathways connecting regions implicated in dystonia pathophysiology.

**FIGURE 1:**
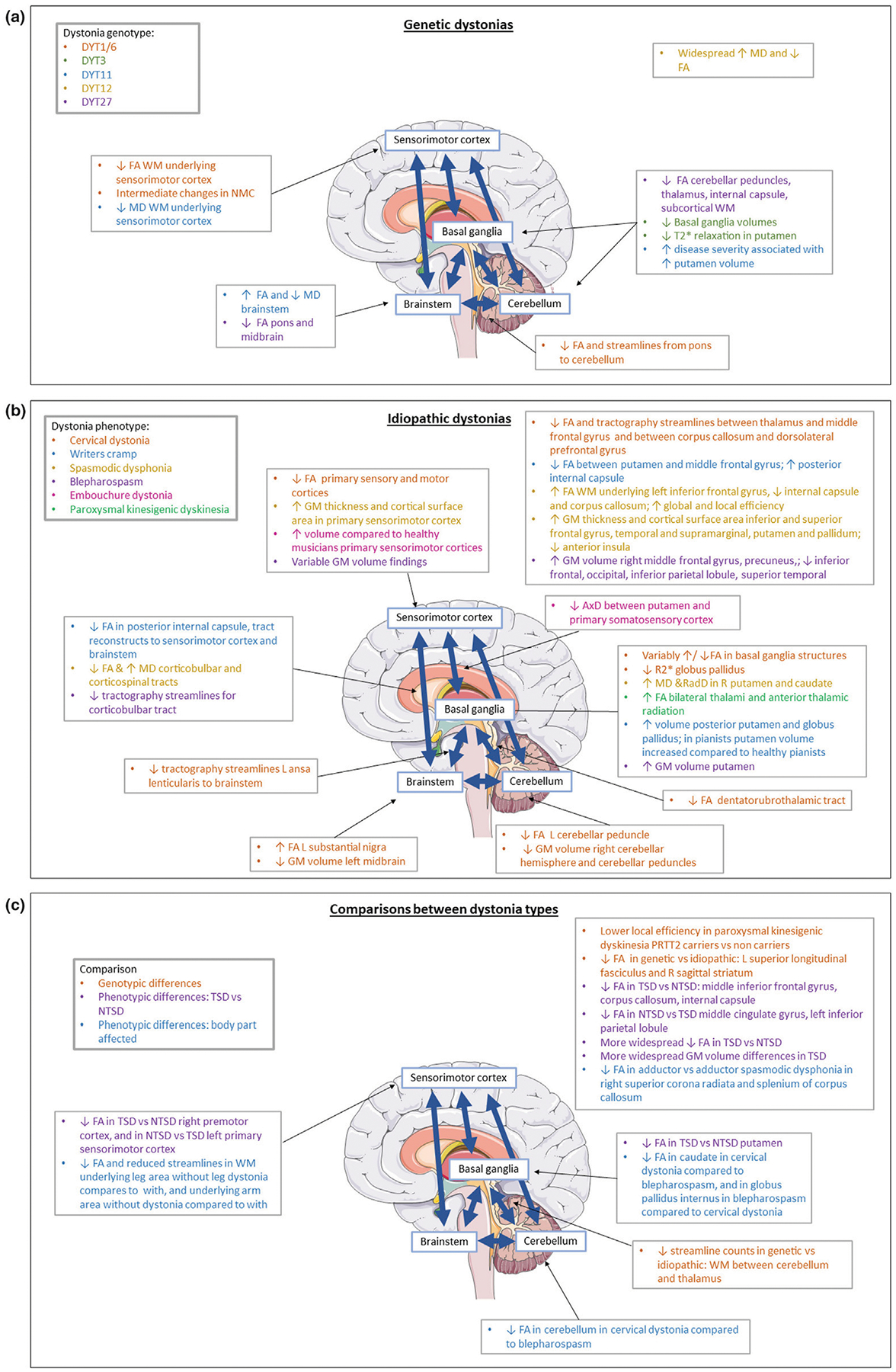
Summary of structural MRI differences relative to healthy controls in dystonia based on genotype, whether the phenotype is task specific, and the body parts affected for (a) genetic dystonias, (b) idiopathic dystonias, and (c) comparing the types of dystonia. FA, fractional anisotropy; GM, grey matter; WM, white matter; L, left; R, right; TSD, task- specific dystonia; NTSD, non- task- specific dystonia. Reproduced with permission from [40].

Structural neuroimaging studies using VBM and diffusion imaging report grey and white matter abnormalities in regions subserving motor execution and sensorimotor integration [34–36]. Although there have been differences between studies and types of isolated dystonia, common abnormalities across the subtypes may occur in sensorimotor, premotor, and parietal cortical areas, basal ganglia, thalamus, and cerebellum [37–39]. MacIver at al provided a critical analysis of methods and results from 37 volumetric and 45 dMRI studies in dystonia [40]. Regional volumetric results appeared highly variable but abnormalities in brainstem, cerebellum, basal ganglia, and sensorimotor cortex occurred most frequently (see Figure 1). Task-specific dystonias exhibited higher grey matter volume than non-task specific dystonias [38, 40]. The white matter pathways connecting implicated brain regions predominantly exhibited lower fractional anisotropy and higher mean diffusivity. Although interpretation of the higher grey matter volume remains unclear, the white matter changes suggest degraded integrity of those pathways, supporting the idea that disruptions across multiple structural connections contribute to dystonia as a network disorder. The genetic dystonias tended to have fewer cerebellothalamic tractography fiber bundles–known as “streamlines” – than the idiopathic dystonias.

Non-task specific dystonias, such as CD and BSP, were found to have more subcortical alterations, whereas task-specific dystonias, such as LD and FHD, were shown to affect cortical structures. In most studies, changes were identified in the somatotopically organized cortical regions corresponding to the body regions affected by dystonia. For instance, changes were observed in the hand sensorimotor region in FHD [41, 42] and the face and laryngeal areas in embouchure dystonia [43] and LD [44], respectively.

#### VBM

Based on VBM, dystonia exhibits distinct grey matter morphological networks because those features distinguish between dystonia and essential tremor and between dystonia and healthy controls with accuracies of 95% and 89%, respectively [45]. On the other hand, in a coordinate-based meta-analysis of 27 VBM studies in dystonia [46], no reliable grey matter volume differences were found in idiopathic dystonia. However, the authors point out that if the subtypes exhibit different volumetric profiles, the inclusion of different subtypes may have diluted the results. In CD, a multimodal meta-analysis of 9 studies [47] found differences across many brain regions, including bilateral precentral and postcentral gyri, bilateral paracentral lobules, right SMA, bilateral dorsolateral superior frontal gyri, left middle temporal gyrus, right inferior parietal gyrus, bilateral median cingulate/paracingulate gyri, lingual gyrus, right caudate, thalamus, and bilateral cerebellum. In LD, a similar multimodal analysis of 21 functional and structural neuroimaging studies, including 31 experiments in 521 LD patients and 448 healthy controls, demonstrated abnormalities in the bilateral primary motor cortices, the left inferior parietal lobule and striatum, the right insula, and the supplementary motor area [48]. In myoclonus dystonia, even the primary visual cortex has been implicated with VBM [49]. Collectively, the large number of regions involved further reinforces the view of dystonia as a network disorder and provides evidence for future investigations probing these targets with new therapies.

#### dMRI

**FIGURE 2:**
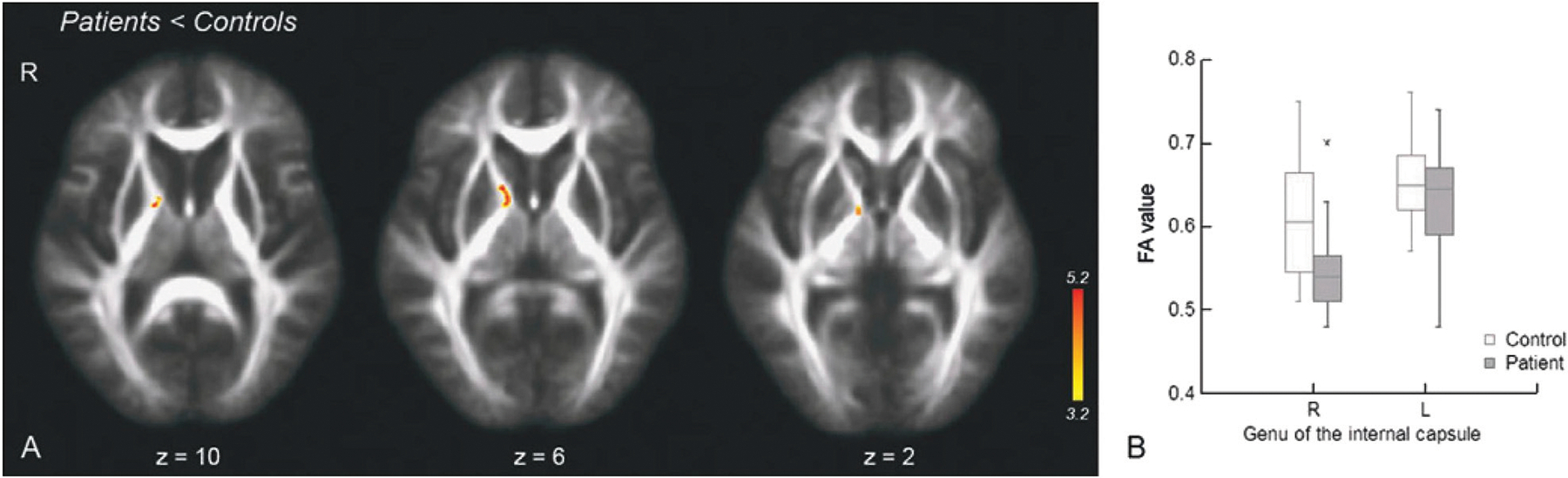
Fractional anisotropy in LD compared to controls. (A) Unbiased whole-brain tract-based spatial statistics (color bar indicates the significance range at Z > 3.2). (B) *a priori* ROI analyses from right genu of the internal capsule (Box plots indicate median and upper and lower quartiles. Error bars indicate the range between the 90th and 10th percentiles. Asterisk indicates significant difference between two groups. R = right; L = left). Reproduced with permission from [50].

DMRI changes were observed in the cortico-striato-pallido-thalamic pathway and the cerebello-thalamocortical pathway across different forms of dystonia. Among the first studies conducted with dMRI in LD patients compared to controls, decreases in white matter integrity were found along the corticobulbar/corticospinal tracts as well as in the brain regions directly or indirectly contributing to these tracts (see Figure 2) [50]. Furthermore, these neuroimaging findings were uniquely substantiated with postmortem brain pathology from a patient with LD and three controls that showed demyelination and degeneration of axonal fibers and clusters of mineral accumulations in the regions found to be abnormal on dMRI.

For BSP and CD, changes were observed in the dentate-rubro-thalamic tract, the brainstem, and the cerebellum [38, 51]. In another study, there were no differences between BSP and CD, but compared to healthy controls both patient groups exhibited fiber loss in the white matter tracts connecting GP, putamen, and thalamus with the primary sensorimotor cortex and SMA [52].

In CD, compared to healthy controls, brain networks exhibited an overall decrease of network strength and increase of local efficiency and node associativity based on graph theoretical analysis of dMRI [53]. Each group was comprised of 30 participants, and the results also held in reproducibility analyses using the Anatomical Automatic Labeling atlas. Quantitative anisotropy based on dMRI has also shown that bilateral tracts between the amygdala and the thalamus have been correlated with transient anxiety, and that bilateral tracts between the amygdala and motor, sensorimotor, and parietal association cortical areas were correlated with more persistent anxious traits [54]. Although there were no differences in TWSTRS and anxiety scales for those on vs. without anti-anxiety medications, the relative timing of the anxiety assay and the imaging were not reported.

In FHD, alterations were reported in white matter tracts connecting the putamen and the dorsal premotor cortex [55], the primary sensorimotor area [56], and the left medial frontal gyrus [57]. In embouchure dystonia, abnormalities were found in tracts connecting the putamen and the primary sensory cortex, the SMA, and the superior parietal cortex [58], and in LD, white matter alterations were described in the superior corona radiata [44].

Differences in structural connectivity based on dMRI were also explored in relationship to the clinical penetrance in carriers of DYT1 and DYT6 mutations [59]. Tractographic analysis disclosed specific changes in the integrity of cerebellothalamocortical (CbTC) pathways, likely of developmental origin, that regulated penetrance and variation of motor and non-motor phenotypes in these individuals [60–62]. Analogous tract changes were also identified in a knock-in mouse model [63, 64].

Taken together, these studies support the hypothesis that dystonia is a network disorder involving networks connecting the striatum, the sensorimotor and fronto-parietal cortices, and the cerebellum.

#### VBM and dMRI heterogeneity

Some results varied between studies and types of dystonia, raising several questions. What would be common or specific to each type of dystonia? What would be due to differences in patient characteristics or imaging protocols that varied between studies? Direct comparisons between different types of isolated dystonia reported interesting findings in this regard. Several studies suggested that the regions affected could differ between task-specific and non-task-specific dystonias using VBM [38, 65] or diffusion imaging [66]. For instance, the cerebellum and the primary sensorimotor areas were commonly affected in both task-specific (FHD and LD) and non-task-specific (BSP and CD) dystonia, whereas regions responsible for dystonic movements (i.e., writing and speaking) were specifically affected in task-specific dystonia [38]. Another study suggested that changes might differ between dystonia types with increased grey matter volume being observed in task-specific dystonia (FHD and LD) and reduced grey matter volume observed in non-task-specific dystonia (BSP and CD) [67]. Finally, some differences in brain abnormalities have also been found when stratifying patients based on their level of training, such as musician’s dystonia, including musician’s FHD and singer’s LD, vs. non-musician’s dystonia, including FHD and LD [65, 68].

In inherited dystonias, structural changes were reported as increased grey matter volume in the right GPi in patients with DYT1 mutation [69], decreased anisotropy in the motor subcortical white matter in patients with DYT1 mutation [70], and reduced cerebello-thalamocortical connectivity in patients with DYT1 and DYT6 mutations [59]. In patients with PRRT2 related paroxysmal kinesigenic dyskinesia, which can exhibit symptoms of dystonia, changes were observed in the basal ganglia cortical network, with reduced grey matter volume in the SMA and right inferior frontal gyrus and reduced mean diffusivity in the left corticospinal tract [71], along with increased fiber density in the cerebellar pathway [72].

Another important question is the relationship between phenotype- and genotype-specific structural alterations. In LD, phenotype-specific changes were observed in the primary sensorimotor cortex and the superior corona radiata, whereas genotype-specific changes were observed in the superior temporal gyrus, the SMA, and the superior longitudinal fasciculus [44]. Two studies have suggested that differences in putaminal volume might represent an endophenotype in inherited dystonia, with increased putaminal volume in asymptomatic DYT1 carriers [73] and unaffected relatives of patients with adult-onset dystonia. The latter had displayed an abnormal temporal discrimination threshold, potentially indicating abnormal sensory processing similar to that seen in their affected relatives [74]. Similarly, putamen volume may be abnormal in people with isolated idiopathic cranial or hand dystonia [75]. Changes in the cerebello-thalamic fiber tract were common to patients with inherited and sporadic dystonias, whereas changes in the thalamocortical fiber tract were only observed in non-manifesting carriers or in non-affected regions of patients with sporadic dystonia [76].

Imaging studies during DBS procedures have also shown the importance of striatal and cerebellar circuits connected to cortical sensorimotor areas. DBS electrodes for dystonia treatment are typically placed in the postero-latero-ventral sensorimotor GPi [77, 78], a key node of the basal ganglia-cortical network. Diffusion-based connectivity between the GPi and the sensorimotor putamen predicted DBS outcomes in CD and correlated with clinical improvement [79]. Effective contacts also localized near the dentato-rubro-thalamic tract [80].

Structural imaging features can further classify dystonia subtypes. Using discriminant analysis, patients with CD and BSP could be distinguished with 100% and 83% accuracy from healthy subjects, respectively. Using more advanced deep learning, patients with LD, CD, and BSP could be distinguished with 98.8% accuracy based on an automatically identified pathophysiological neural network biomarker [81].

#### Limitations of structural imaging

Structural imaging studies in CD [82] and FHD [83] sometimes reported negative findings. Many were underpowered or used uncorrected statistical thresholds, yielding subtle effects. These limitations highlight the importance of studying large groups of patients with robust statistics, encouraging multicentric and international collaborations, especially for inherited rarer forms of dystonia. Because most studies are cross-sectional, they do not allow for determining whether the observed structural changes were the cause or the consequence of the disease, e.g., to dissociate pathophysiological hallmarks from compensatory mechanisms. Studies of the effect of treatment in asymptomatic carriers and longitudinal studies–and more broadly careful alignment of study goals and designs [84] – could provide important information in this regard. The neuropathological correlates of structural imaging changes are still poorly understood. Whether they correspond to changes in cell or fiber number, shape, size, dendritic arborization, or tissue architecture is not known. This insufficient knowledge highlights the need for 1) the development of new validated imaging techniques to study *in-vivo* microstructural changes related to cellular organization (e.g., with diffusion weighted magnetic resonance spectroscopy [85] and 2) comparative imaging and histological studies in animal models and post-mortem human tissue.

#### Summary of structural imaging

Despite these limitations, structural imaging consistently implicates the cortico-striato-pallido-thalamic pathway and the cerebello-thalamocortical pathway in dystonia. They also suggest that not only brain regions involved in these networks but also connections between them are abnormal [35], in line with the view of dystonia as a network disorder. The results from structural imaging also lay the foundation for evaluating the network physiology that is assayed with functional imaging.

### Functional near-infrared spectroscopy (fNIRS)

Functional near-infrared spectroscopy (fNIRS) measures changes in the concentrations of oxygenated and deoxygenated hemoglobin in the cortex. FHD patients exhibit task-specific patterns in their oxygenated hemoglobin distinct from healthy controls [86]: writing increased activation in the right and left motor cortex and SMA, whereas finger tapping decreased activation in the left sensorimotor cortex and bilateral SMA.

### Positron emission tomography (PET)

#### PET measures of metabolism

**FIGURE 3:**
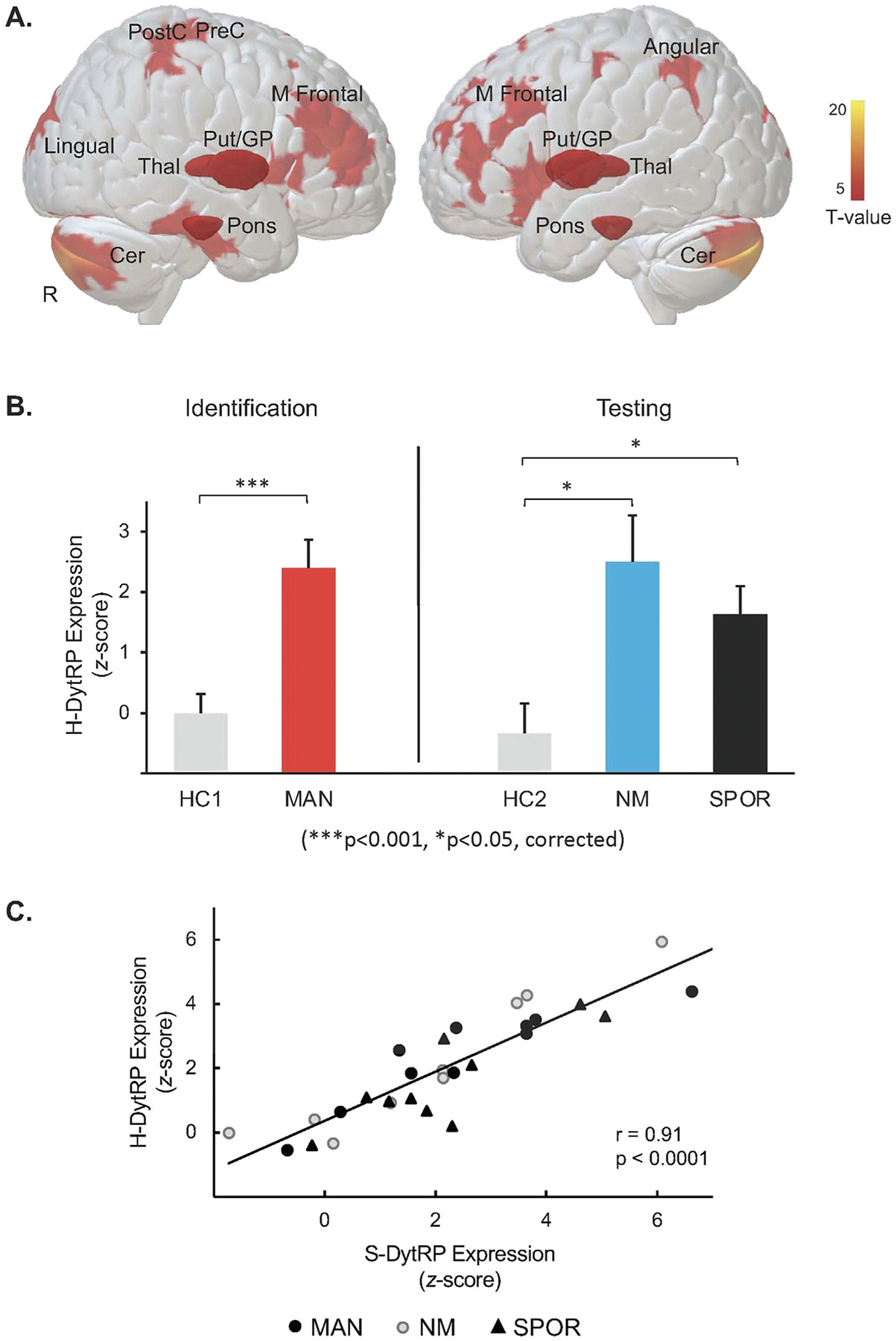
Hereditary dystonia-related pattern (H-DytRP). (A) H-DytRP identified in rs-fMRI scans from manifesting (MAN) gene carriers and healthy control (HC1) subjects. This network was characterized by local contributions from the cerebellum, basal ganglia, thalamus, sensorimotor cortex, and frontal and parieto-occipital association regions. (B) Left: Expression scores for H-DytRP were elevated in the MAN group compared to the HC1 subjects used in network identification. Right: Significant increases in network expression were also seen in the non-manifesting (NM) mutation carriers and patients with sporadic dystonia (SPOR) compared to HC2 testing subjects. (C) Expression values for the H-DytRP were highly correlated with corresponding subject scores for a similar sporadic dystonia-related pattern (S-DytRP) identified in an analysis of the SPOR data. (Cer, cerebellum; Put/GP, putamen/globus pallidus; Thal, thalamus; PostC, postcentral; PreC, precentral; M frontal, middle frontal). T-map thresholded at 4.8 (P < 0.001); color stripe. Error bars represent standard error of the mean. ***P < 0.001; *P < 0.05 relative to HC, corrected for multiple comparisons. Reproduced with permission from [192].

Before fMRI became widespread, PET already illuminated dystonia pathophysiology through altered metabolism and neurotransmission. In 1984, PET revealed basal ganglia dysfunction contralateral to hemidystonia in a person that had normal CT and MRI scans [27]. Later PET studies focused on metabolic brain imaging with 18F-fluorodeoxyglucose (FDG) as a marker of local synaptic activity [87, 88]. Using spatial covariance analysis [89], a reproducible dystonia-related metabolic pattern, termed DytRP, was identified in patients with genotypic and sporadic forms of the disorder [90, 91]. The DytRP network was characterized by significant contributions from the putamen, pons, cerebellum, and sensorimotor cortex. In addition to being expressed in patients with sporadic dystonia, subject scores for this pattern were elevated to a similar degree in manifesting (MAN) and non-manifesting (NM) carriers of DYT1 and DYT6, suggesting that DytRP expression may, in certain populations, be an endophenotypic trait. Results from these PET studies served as the foundation for a “Dystonia-related pattern” subsequently evaluated with rs-fMRI (see Figure 3). Networks involving the cerebellum are also differentially affected by modulators of the GABAA system: in FTSD, compared to placebo, Zolpidem induced hypometabolism in the right cerebellum and hypermetabolism in the left inferior parietal lobule and left cingulum [92].

#### PET measures of neurotransmitter systems

Neurotransmitter-specific PET studies have mostly focused on abnormalities in dopaminergic pathways–especially within the putamen, which has among the highest density of dopamine receptors in the brain. PET studies have also identified GABAergic and possible cholinergic abnormalities. Each of these neurochemical changes reflects regional contributions to the broader dystonia network.

PET studies identified functional abnormalities not easily identifiable by structural imaging, and in multiple cases implicated the putamen, hinting at possible dopaminergic dysfunction. The first demonstrated striatal (later identified as putaminal) alterations in blood flow and oxygen extraction and metabolism contralateral to the affected side of the body in an individual with post-traumatic paroxysmal hemidystonia [27]. This individual had normal anatomy as visualized by CT and MRI. With the methods available at the time, no obvious abnormalities were found in resting blood flow measures in people with isolated, idiopathic dystonia. This lack of resting-state abnormality led to a second series of observations reporting abnormalities in vibration-induced blood flow responses in sensorimotor cortex and SMA in those with unilateral, isolated, idiopathic dystonia [20] unilateral writer’s cramp [93] and cranial dystonia [94]. Interestingly these abnormalities were not only contralateral to the affected side in the limb dystonias but also in the ipsilateral side of the brain. A hint that these findings related to striatal dysfunction came from a study showing the same reduced vibration-induced blood flow that normalized after oral levodopa in participants with dopa-responsive dystonia [19]. Yet, these indirect measures did not directly prove dopaminergic dysfunction.

Direct PET measures of dopaminergic radioligands did demonstrate abnormalities in striatal dopaminergic systems. First, patients with idiopathic, isolated cranial and hand dystonia had reduced striatal binding of a D2-like radioligand ([18F]spiperone) [10] that matched the transient reduction found in an animal model of transient dystonia induced by internal carotid infusion of the selective dopaminergic neurotoxin MPTP [10, 95]. These abnormalities were later determined to be somatotopically organized in the putamen based upon the part of the body affected by dystonia [96]. Subsequent studies confirmed these D2-like changes with a more specific radioligand, [11C]raclopride, in manifesting or nonmanifesting carriers of mutations in DYT1 or DYT6 dystonia [90, 97] as well as in those with idiopathic, isolated FHD or LD [98, 99]. These latter two studies also took advantage of [11C] raclopride, which is displaced by the release of endogenous dopamine, to demonstrate the somatotopically related abnormal phasic striatal release of dopamine in response to symptomatic and asymptomatic motor tasks. However, [18F] spiperone has specific binding to all D2-like receptors, including D2, D3, and D4 dopamine receptors. In contrast, [18F]N-methylbenperidol has a 200-fold greater affinity to D2 receptors compared to D3 or D4, and the application of [18F]N-methylbenperidol did not reveal any striatal differences in D2 receptor binding in a cohort of patients with idiopathic, isolated FHD and cranial dystonia suggesting the previously found D2-like abnormalities may be mediated by D3-specific receptors [100]. An earlier study using [11C]NNC-112, a D1-like selective radioligand also did not find striatal differences in a mixed cohort of patients with isolated hand, cranial, and cervical dystonia [101], whereas using a higher resolution scanner in better-stratified patient cohorts permitted identification of somatotopically related abnormalities in isolated FHD and LD patients [102]. Taken together, these striatal dopaminergic abnormalities were thought to represent dysfunction of the D2-mediated indirect pathway that is important for surround inhibition of unwanted movements during selected motor activity [19], while abnormalities in the D1-mediated direct pathway could reflect excessive action of the pathway important for selective motor activation [102].

Several other studies focused on GABAA receptors using the radioligand [11C]flumazenil. One early study with a small number of participants with either DYT1 dystonia or isolated, idiopathic dystonia found reductions in sensorimotor cortex [103]. In contrast, a larger study focusing solely on isolated, idiopathic CD found higher uptake in the right precentral gyrus and left parahippocampal gyrus and no regions with significant reductions. Interestingly, decreased uptake in cerebellar hemispheres correlated with severity, whereas decreased vermis uptake correlated with disease duration [104]. Higher [11C] flumazenil uptake also occurred in those with idiopathic, isolated FHD in the inferior frontal gyrus but decreased in the cerebellar vermis [105]. Together, these findings implicate abnormalities of cerebellar GABAA and suggest abnormalities in various cortical regions that would fit with dysfunction of cortical inhibition.

Initial studies of cholinergic function using a vesicular acetylcholinergic transport radioligand [106] indicate that posterior putamen may have lower uptake but that this may only occur in younger patients with DYT1 dystonia, while a reduction in cerebellar vermis does not depend upon age [107]. Given the functionally significant role of ACh in the striatum [108], and in particular how it modulates thalamostriatal transmission [109] and the D1- and D2-mediated pathways, PET imaging with ligands for the cholinergic system merit further investigation.

What does all of this mean, and how does it advance our understanding of network mechanisms underlying dystonia? It is likely that selective transmitter abnormalities occur in various forms of dystonia, and commonalities exist across the different forms. Such common findings include dysfunction of striatal dopaminergic systems in isolated focal, idiopathic dystonias, as well as DYT1 and DYT6 dystonias [10, 90, 95–97]. Yet, some abnormalities may be somatotopically organized, reflecting the affected body parts [98, 99, 102]. The dopamine-dependent changes in striatum are consistent with a large body of evidence pointing to a role for dopamine in mediating abnormal synaptic plasticity that could play a role in motor reinforcement learning and the corresponding development of abnormal functional circuits involving the striatum [110, 111]. The striatal dopamine system abnormalities also led to the notion of hypofunctional indirect and hyperfunctional direct basal ganglia pathways [102] with subsequent reduction of cortical inhibition consistent with abnormalities of vibration-induced blood flow responses in idiopathic, isolated dystonia [20, 93, 94] and physiologic observations of reduced cortical inhibition [112]. These data on reduced inhibition are consistent with abnormalities of GABAA receptors in a network that mediates cortical inhibition [103–105]. Although the precise role of cholinergic systems remains to be determined, this complex network interplay likely involves brainstem nuclei, dysregulated thalamostriatal transmission, and a cascade of changes in the D1-mediated direct and D2-mediated indirect pathways through the basal ganglia. Of course, data about cerebellar dysfunction fits well with the increasing understanding of the anatomic and functional relationships between basal ganglia networks and cerebellum [113, 114], including potential relationships between the cholinergic cerebellar vermis with basal ganglia and cortical regions [115, 116].

Most importantly, multimodal studies that combine neuroimaging of transmitter systems with physiologic measures or resting-state functional connectivity will help take these investigations to the next level. For example, the specific putaminal location of a D1 receptor abnormality found with PET [102] was used as a seed for a resting-state functional connectivity study that facilitated identification of specific dysfunctional small-scale networks in people with idiopathic, isolated focal dystonias, whereas rigorous quality control measures eliminated the statistical significance of apparent large-scale, global network abnormalities [117]. Thus, advances in understanding the underlying network dysfunction related to various forms of dystonia will be facilitated by studies that combine various modalities, such as different imaging techniques and other physiologic measures.

### Functional MRI (fMRI)

Functional MRI has yielded important insights into dystonia pathophysiology. It enables simultaneous assessment of distant structures at rest (rs-fMRI) or during tasks, though practical constraints can limit participation for severe generalized dystonia. Most studies focus on focal dystonia and demonstrate impaired brain-network properties.

#### Resting-state fMRI (rs-fMRI)

Intrinsic functional features of brain connections at rest reveal task-free properties of brain networks without the online confound of behavioral performance that may differ between populations. Compared to neurologically normal controls, patients with focal [65, 118–121] and generalized dystonias [122] show either reduced or excessive inter-regional correlations. Because these correlations depend on underlying white-matter architecture, some rs-fMRI abnormalities likely overlap with diffusion-MRI findings.

A meta-review of 46 dystonia rs-fMRI studies [123] most often implicated the sensorimotor cortex, SMA, putamen, parietal cortex, thalamus, and cerebellum, with connectivity changes primarily in the sensorimotor network [123]. Mixed directions of effect likely reflect differences in analytic choices, quality control, and cohort. While common dysfunctions may be part of a general hallmark of dystonia, dysconnectivity patterns in particular networks vary among the different forms of dystonia.

In CD in particular, a meta-analysis of 17 studies using anisotropic effect size-based signed differential mapping (AES-SDM) identified abnormalities in many regions, including bilateral precentral and postcentral gyri, bilateral paracentral lobules, right SMA, bilateral median cingulate/paracingulate gyri, right caudate nucleus and thalamus, right cerebellum and lingual gyrus, right fusiform gyrus, and bilateral precuneus [47]. Sensory network dysfunction at rest encompasses cross-modal sensory areas [124]. Sensorimotor connectivity differs by sensory trick—decreased in patients with a trick and increased in patients without a trick [125]. Perhaps relatedly, connectivity between cortex and cerebellum decreased proportional to BoNT efficacy [126]. Furthermore, compared to healthy controls, the lower range of motion and compromised movement quality during a head “reaching task” seen in CD correlate with decreased functional connectivity among SMA, occipital cortex, and cerebellar regions [127]. In CD patients with GPi-DBS, optimal stimulator settings (compared to non-optimal and stimulator off) reduced activity in sensorimotor cortex in proportion to long-term clinical improvement, and a similar trend appeared in a few cases of generalized dystonia [123, 128].

In BSP, rs-fMRI links spasm intensity to cerebellar and sensorimotor cortical activation, and spasm onset to involvement of the basal ganglia and frontal eye field portion of the superior frontal gyrus [129].

In FHD, rs-fMRI shows distorted digit representation in the somatosensory cortex [130]. It also reveals dysfunctional cortico-subcortical circuits involving somatosensory cortex, primary and secondary motor areas, cerebellum, and basal ganglia [120, 121, 131]. Similarly in musician’s dystonia involving the hand, when compared to healthy musicians, resting state connectivity is reduced within the basal ganglia network [132] but increased in the basal ganglia associative loops with the dorsolateral prefrontal cortex and the premotor cortex [133].

In LD, rs-fMRI demonstrated abnormal functional connectivity within sensorimotor and frontoparietal networks compared to healthy individuals as well as phenotype- and genotype-distinct alterations of these networks, involving primary somatosensory, premotor, and parietal cortices [58, 118]. Battistella et al. [118] was also the first to apply a machine learning algorithm (linear discriminant analysis) to brain imaging data to show the feasibility of this approach for classifying LD patients as distinct from healthy controls with a 71% accuracy based on their differences in the connectivity measures in the left inferior parietal and sensorimotor cortices. When categorizing between different forms of LD, the combination of measures from the left inferior parietal, premotor, and right sensorimotor cortices achieved 81% discriminatory power between familial and sporadic cases, whereas the combination of measures from the right superior parietal, primary somatosensory, and premotor cortices led to 71% accuracy in the classification of adductor vs. abductor forms of LD. Although risk factors for LD remain unclear, in a study using precise demographic and clinical characterization in a large cohort of patients, environmental factors influencing sensory feedback processing explain neural alterations in the parietal, insular, and sensorimotor cortical regions [134].

In a single study involving multiple forms of focal dystonia, dysfunction of sensorimotor cortex and prefrontal division of the thalamus represented a common hallmark of task-specific focal dystonia [68]. A recurrent finding in resting-state studies is the abnormal connectivity between parietal and premotor cortices in different forms of focal dystonia [65, 68, 118, 119, 135–141] and in generalized dystonia [122].

Some forms of genetic dystonia show dysfunction of cerebello-cortical and cerebello-striatal loops [72, 107]. The abnormal outputs from cerebellar cortex to deep cerebellar nuclei would in turn increase the drive of deep cerebellar nuclei to the thalamus, a mechanism that likely plays a critical role in the pathophysiology of dystonia [35]. For instance, in PRRT2 patients, abnormal cerebellar drive toward the thalamic relays of the striatum and motor cortex was partly normalized after cerebellar non-invasive stimulation compared to placebo [72]. In addition, in DYT1 patients, increase of brainstem-striatal functional connectivity was associated with the binding potential of cholinergic ligand in the striatum [107]: higher functional connectivity was associated with lower expressions of acetylcholine vesicular transporter. This suggests concomitant and interdependent functional impairments of cerebellar and striatal nodes.

Another key factor is the role of quality control for analysis of these resting state studies, as less rigor can lead to many statistically significant, yet spurious findings [117]. This is particularly important for interpretation of meta-analyses of numerous studies each of which may not apply such rigor consistently.

#### Task-related fMRI: motor dysfunction

**FIGURE 4:**
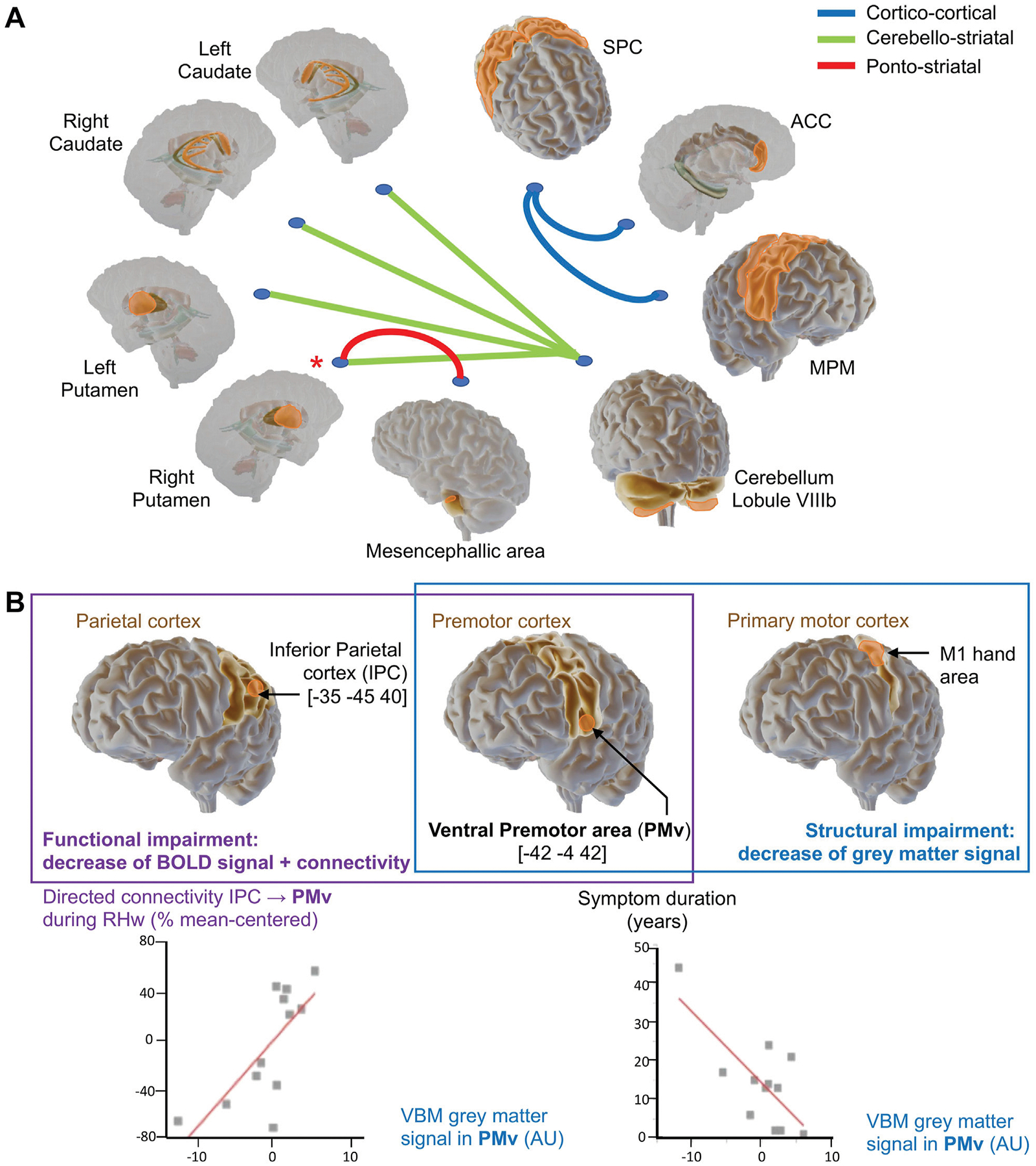
Networks showing functional alterations correlated with structural impairments in dystonia. (A) Compared to controls, DYT1 patients show decreased resting state functional connectivity in striatal, cerebellar, and cortical networks. Large-scale network involving the cholinergic system is altered in some genetic forms of dystonia. Asterisk (*) indicates that mecencephalo-striatal connectivity correlates with deficient binding of ACh PET-ligand in the putamen. (MPM, motor premotor; ACC, anterior cingulate cortex; SPC, superior parietal cortex). Adapted from [108]. (B) WC patients showed task-specific decrease of activation and directed connectivity between the inferior parietal cortex (IPC) and the ventral premotor cortex (PMv) during right hand writing. This was accompanied with decrease of grey matter volume in the M1 hand area and in the task specific PMv. PMv appears as an important hub in task-specific dystonia, linking structural with functional deficits and clinical characteristics of focal hand dystonia. Adapted from [151].

Considering motor tasks, network dysfunction is present at all stages of motor control, i.e., motor planning, motor preparation, and motor execution. During task periods preceding movement onset or the imagination of hand movements, FHD patients have impaired cortical and basal ganglia activation [142, 143] that sometimes extends to the cerebellum [142]. Abnormal motor planning was often related to task-related dysfunction of parietal and lateral premotor areas during imagined movement [144, 145] and during the execution of right (symptomatic) handwriting compared to other tasks (tapping, zigzagging) performed with different limbs (left hand, right foot) [146]. In particular, an increase in practice-related activity in the premotor cortex, later associated with motor consolidation, suggests the formation of abnormal motor engrams [55]. During motor execution, hyperactivity in primary somatosensory and motor cortices were generally observed in different forms of focal dystonia [147–151], and in myoclonus dystonia [152]. For CD, BSP, and LD, task-related dysfunctions were demonstrated in cortical, cerebellum and/or basal ganglia activation during non-symptomatic tasks [82, 148, 153–156]. In the context of LD, when ADSD patients were compared to controls, cerebellar activation was reduced during symptomatic phonation and modified during the asymptomatic tasks [148, 157]. Network regions involved in different forms of dystonia for symptomatic [144, 146] and non-symptomatic [156, 158, 159] tasks may indicate that motor commands are elaborated and executed through a common pathway that contributes importantly to the pathophysiology of dystonia (see Figure 4). A recent study showed abnormal involvement of cognitive and visual networks during rest periods interleaved with task execution [159]. Task-related connectivity studies showed that patients with focal dystonia have changes in the strength of cortico-cortical motor and cortico-basal ganglia connections, and abnormal cerebello-thalamocortical connections [55, 140, 160]. Task-related connectivity in dystonic hand tremor showed specific involvement of associative cortical, cerebellar and striatal regions [161].

Beyond parieto-premotor cortices and cerebellar regions, larger studies of focal dystonia patients (n>= 30) showed altered connectivity in broader networks encompassing insular [162, 163] and prefrontal areas [164] extending the dysfunctional network to cognitive-limbic associative areas. For instance, these findings were observed during reward learning with increased activation in the anterior cingulate cortex [165]. They were also observed when resting-state connectivity was associated with offline task performance, as abnormal communication between cerebellum and pre-SMA correlated with impaired agency (the loss of perception of control over one’s action) during a visuomotor task [166].

#### Task-related fMRI: sensory dysfunction

Several paradigms allow for investigating task-related sensory dysfunction, including sensory stimulation of a body part, passive movements, and discrimination in time or space between two sensory stimuli. Patients with isolated dystonia affecting a specific body part showed an abnormal representation of the symptomatic limb in the somatosensory cortex, with excessive overlap of cortical representation of digits for writer’s cramp [130] and of mouth for embouchure dystonia [167], as well as abnormal activation of lips, face, or digit areas during sensory stimulation [168, 169]. Sensory stimulation also engaged dysfunction of the primary sensory cortex unrelated to the affected limb [170, 171] and other brain areas, including GPi [172], striatum and cerebellum [61, 173, 174]. Dysfunction of the putamen was often observed in different forms of dystonia during tasks involving perceptual judgements [175–177], with dysfunction that extended to the superior colliculus in CD [171] and insular cortex in FHD [176]. In LD, abnormal temporal discrimination was associated with dysfunction in the middle frontal and primary somatosensory cortices, while cerebellar and striatal dysfunctions were form (sporadic/familial)- or symptom-specific [178]. However, contrary to altered visual temporal discrimination, auditory temporal discrimination and olfactory function have not been found to be statistically altered in LD patients, suggesting that these are likely not candidate endophenotypic markers of LD [179].

Obviously, sensory processing is often coupled with motor output. In daily life, perception and action are part of an interactive cycle, as we perceive the results of our action through our senses, and sensory feedback are used to initiate or correct our movements. In task-specific FHD, conditions during which somatosensory and proprioceptive information is used to further plan the movement, elicited impaired activation of primary sensorimotor cortices, as well as posterior parietal and premotor areas [169]. This importantly suggests that parieto-premotor dysfunction is also present during the delay when patients had to use somatosensory information for motor planning.

#### Nodal weighting

Because dystonia appears to involve larger networks than originally thought, investigating the relative weight of individual regions (nodes) in the functional and structural networks can provide a better understanding of the network’s complex disorganization. Moreover, focusing on pathological models with dysfunction in a particular node could help answer the question of the node’s contribution within the network. In this line of reasoning, a study considered two genetically modified mouse models of DYT1 dystonia, the first had conditional knock-in (KI) in neurons that express dopamine-2 receptors (D2-KI), while model 2 had conditional KI in Purkinje cells of the cerebellum (Pcp2-KI) [180]. The results suggest that, in DYT1, dopaminergic D2 neurons have detrimental effect on sensory functions and functional connectivity, whereas the cerebellum functional role within the sensorimotor network protects against dystonia-like motor deficits. Cerebellar involvement in FHD depends on the complexity of symptoms, also suggesting a compensatory role of the cerebellum [160]. Along the same line of reasoning, a rare form of dystonia with ADCY5 mutation presents a primary dysfunction within the striatum but not in the cerebellum [181]. The ADCY5 pathological model provides the unique opportunity to test how a primary striatal dysfunction affects cerebellar activity, which we expect could compensate for striatal dysfunction. However, in the most common forms of dystonia, whether cerebellar abnormalities are primary or secondary to striatal dysfunction remains unclear. In a study involving patients with PRRT2 mutation, which induces dystonia among other hyperkinetic symptoms [182], aberrant cerebellar output can drive striatal dysfunction [72]. In some cases, the cerebellum can have detrimental influence within the sensorimotor network such as in dystonic tremor [183], or in myoclonus dystonia (DYT11) [166, 184]. Despite its systematic involvement in multiple forms of dystonia, the cerebellum likely has different functional weights within the sensorimotor network, depending on environmental and genetic factors.

#### Network analytics

Eidelberg et al. developed a method to map disease networks in rs-fMRI data based on independent component analysis (ICA) [185, 186]. Applying this approach to scans from clinically manifesting (MAN) dystonia mutation carriers and healthy control subjects, an rs-fMRI-based DytRP was identified (Figure 3A) with topographic features similar to its earlier PET counterpart [187]. As with the PET-based DytRP, expression of the rs-fMRI network was elevated in NM mutation carriers and in patients with sporadic dystonia (Figure 3B) and correlated with clinical dystonia ratings measured in affected individuals. This network mapping approach also allowed for detailed analysis of the functional connections linking DytRP nodes, as had been undertaken previously in Parkinson’s disease [188, 189]. Genotypic and sporadic dystonias were both characterized by positive correlations between CbTC and pontine DytRP regions, suggesting distinct facilitatory nodal interactions in these groups. This contrasted with the negative correlations between CbTC nodes that were present in healthy subjects. Of note, increases in cortico-striatal or cortico-cortical connectivity were more pronounced in patients with genotypic forms of the disorder.

Recent studies of disease network architecture using graph metrics such as degree centrality, clustering, path length, and small worldness [187–190] identified differences in the patterns of functional connectivity in dystonia mutation carriers with and without motor manifestations. The data overall show how network mapping and graph theoretic methods can provide novel insights into the circuit abnormalities that underlie isolated dystonia. Other methods consider the inter-dependent involvement of cortical and cerebellar nodes, given the degree of convergence between cortical inputs on cerebellar nodes [191]. Such methods could bring more insight into the functional roles of the cerebello-cortical subdivisions in the pathophysiology of dystonia.

#### Summary of fMRI

The conclusions drawn from these resting-state and task-related studies converge towards 1) common networks that are affected in most forms of dystonia involve sensory cortices, striatum, and cerebellum; 2) cortico-cortical projections associated with abnormal representation of fine motor skills, i.e., parieto-premotor connections; and 3) network dysfunction extending to include cognitive-limbic associative nodes. Whether certain nodes have deleterious or beneficial contribution to behavioral output or clinical symptoms should be probed using specific pathological models and/or neuromodulation strategies [72] used in conjunction with fMRI.

### Electroencephalography (EEG)

Although used in only a few studies of dystonia, EEG is one of the oldest non-invasive measures of brain function [4, 192] and allows comparisons of activity between groups at rest and during tasks. While scalp recordings generally reflect underlying cortical activity, source-modeling techniques enable deeper localization. EEG signals can be decomposed into frequency bands since different frequencies reflect different brain processes. Thus, it is possible to get frequency information over time from various brain locations, largely limited to the cortex. Correlations between pairs of EEG channels can indicate communication between regions or indicate that both are jointly influenced from a third source. Correlations are referred to as functional connectivity. Using more sophisticated algorithms, it is possible to identify the causal influence of one region on another–this is called effective connectivity. The analysis of all (or many of) the possible connections in one model is called graph theory, and this gives a more systemic picture of the brain network.

Most of the studies applying EEG to dystonia have been done in FHD. Somatosensory evoked potential studies show distorted digit representation in the somatosensory cortex [130, 193]. FHD patients also exhibit task-specific patterns in their EEG distinct from healthy controls [86]. Abnormal shape and amplitude of readiness potential were observed during motor preparation [194]. In the bilateral sensorimotor cortex, writing elicits increased low gamma power and less mu-beta and beta attenuation. There is also reduced connectivity between the SMA and the left sensorimotor cortex. During finger-tapping, patients failed to attenuate the mu-alpha, mu-beta, and beta power, and there were no changes in connectivity.

**FIGURE 5:**
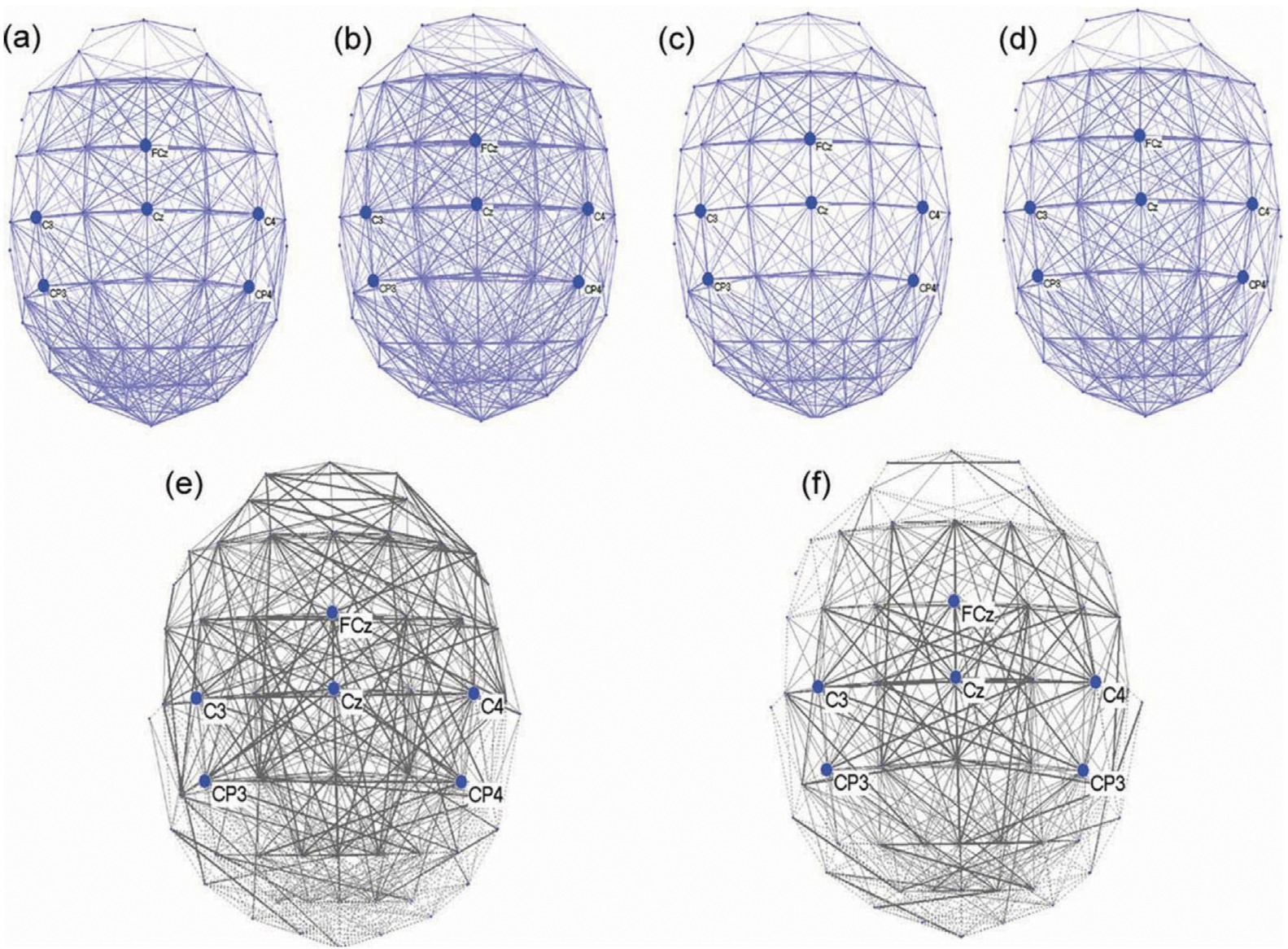
EEG beta band functional connectivity in healthy volunteers in rest (a) and task (b) conditions and in FHD patients in rest (c) and task (d) conditions. Corresponding task-related changes in healthy volunteers (e) and FHD patients (f), with solid lines indicating increased connectivity during a task and dotted lines indicating decreased connectivity during a task. Six bold nodes are channels of interest. Reproduced with permission from [200].

**FIGURE 6:**
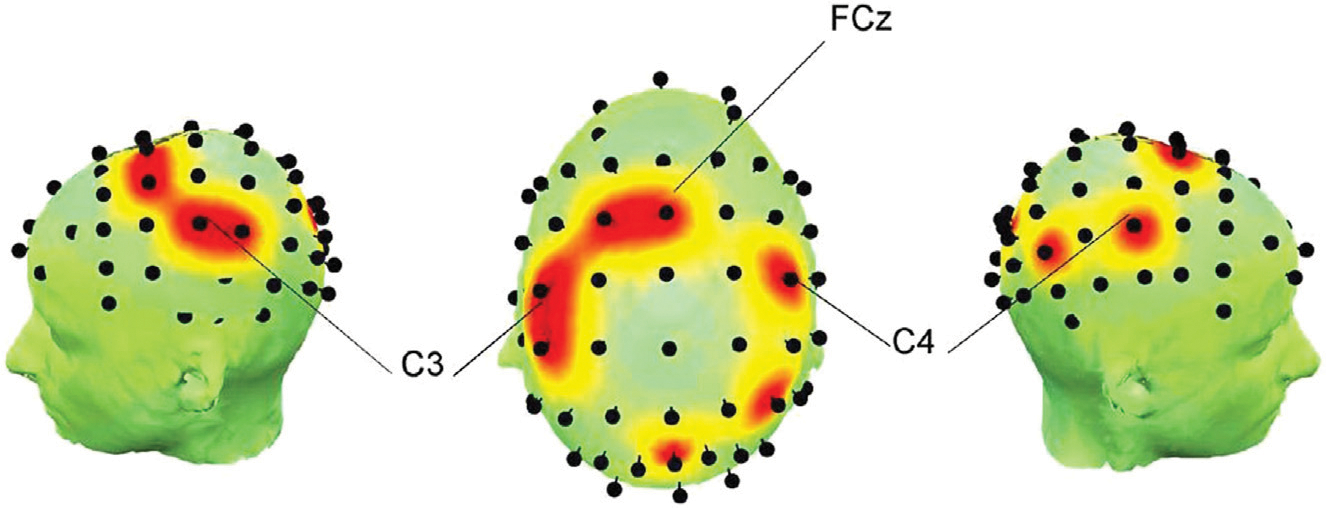
Normalized spatial distributions of *Enodal*, a measure of global communication efficiency in beta band at each node, viewed from the left, top, and right aspects. Labelled channels exhibit significantly different *Enodal*, at a cost of 0.28 corresponding to the maximal interaction in *Eglob* differences. Main group effect found at FCz, corresponding to the SMA. Reproduced with permission from [202].

Brain connectivity in FHD compared with healthy controls was studied with 58-channel EEG using a technique called mutual information analysis [195]. Studies were done at rest and during a simple finger tapping task that did not produce dystonia. Mutual information is a measure of linear and non-linear coupling and was computed in alpha, beta, and gamma frequency bands. Most of the interest was linear and in the beta band. The task produced increased mutual information in both groups. However, mutual information was decreased in the patients at both rest and in action (see Figure 5). The data from healthy volunteers were then analyzed with graph theory using a measure of efficiency [196]. Efficiency during the finger tapping was increased selectively in the beta band, and regional efficiency was most increased in bilateral primary motor and left sensory area. A similar analysis was done in the FHD patients with different results [197]. While the beta network was efficient at rest, the efficiency decreased with the motor task (see Figure 6). Evaluating the regional efficiency, there was an increase over the SMA, but it decreased with the motor task. Collectively, the findings indicate an abnormal network at rest, greater disruption with a motor task, and motor area inefficiencies.

Several subsequent similar studies in FHD all used different methods. One study employed an isometric movement that did not induce dystonia, utilized magnetoencephalogram (MEG), and focused specifically on coherence between sources at M1 and S1 [198]. Coherence in different frequencies was similar at rest and was reduced only during movement in patients and only in the gamma band. A second study, again using non-symptomatic movements, used effective connectivity of EEG and machine learning [199]. The most sensitive difference between patients and heathy volunteers was a decrease in beta effective connectivity during movement from the contralateral premotor area to other nodes. A third study using EEG coherence looked at writing, sharpening a pencil (a task that did not induce dystonia), and imagination of those same two tasks [200]. The only abnormality identified was a reduction of interhemispheric alpha coherence between the two motor areas and only during actual handwriting. A fourth additional study used EEG transfer entropy at rest and during writing to evaluate effective connectivity [201]. They used graph analysis metrics and found reduced nodes in the beta frequency during writing. When investigating imaginary coherence during the processing of somatosensory information used to plan sequential finger movements, communication between parietal and frontal electrodes was decreased at mu and beta frequencies in writer’s cramp compared to healthy controls [169].

In LD, symptomatic speaking was compared to two asymptomatic tasks–whispering and writing–using high-density EEG [202]. Speaking produced increased gamma synchronization in middle/superior frontal gyri, primary somatosensory cortex, and superior parietal lobule, with disrupted prefrontal–parietal coupling. Writing showed decreased beta synchronization, most prominently in right superior frontal gyrus; whispering was normal.

Despite methodological differences, results converge: focal dystonia shows sensorimotor-network disconnection—stronger during movement than rest—primarily affecting prefrontal–parietal links, with abnormalities variably in alpha, beta, or gamma bands. EEG/MEG therefore provide valuable spatiotemporal insight; combined with DBS they can probe cortical–subcortical coupling, and improved montages/analytics may enable deeper source localization and open new horizons for investigating cerebello-cortical electrophysiological signals [203].

## Deep brain stimulation (DBS)

For some types of dystonia–especially including but not limited to the generalized forms–DBS has been a revolutionary treatment. DBS also enables direct probing of dystonia networks. First, DBS activation, combined with imaging, can point to associated network changes. Effective DBS for dystonia–usually in the GPi–is associated with increased metabolism not only at the stimulation site but also in related network nodes of STN, putamen, and primary sensorimotor cortices [204]. GPi DBS also normalizes functional coupling patterns in the basal ganglia, thalamus, and brainstem [205]. Although less commonly used in dystonia, preliminary evidence suggests that STN and thalamic motor targets can show subtype-specific efficacy for BSP, CD, and appendicular forms of dystonia [206].

Second, DBS in unconventional targets can provide additional evidence about the regions and networks implicated in dystonia. DBS in the field of Forel in a small series of otherwise refractory dystonia cases, including one each of lingual, cranio-cervico-axial, and hemidystonia, implicates the pallidothalamic tracts, i.e., the primary GPi output to thalamic nuclei [207]. Likewise, the pedunculopontine nucleus (PPN) has been implicated in dystonia [208] and although not usually a DBS target for isolated dystonia, DBS in PPN can decrease the axial dystonia evident in Parkinson’s disease [209].

Third, and perhaps more significantly, the implantation of DBS electrodes for the treatment of dystonia provides an otherwise rare opportunity to understand network abnormalities through invasive brain recording in humans. Invasive human brain recording is evolving in several ways. Earlier work was based on brief recordings done intraoperatively or via leads externalized temporarily. The new availability of commercial DBS devices that provide brain sensing as well as stimulation, allows a shift to a chronic recording paradigm [210]. Paired with wearable monitors of motor function, chronic brain recording is ideal for a deeper understanding of personalized neural signatures of specific motor signs. The field of invasive recordings is also transitioning from single site recording (basal ganglia only) to multisite recordings, which can include other subcortical regions as well as sensory and motor cortex through insertion of electrocortigraphy leads through the same surgical exposure as the DBS leads.

### DBS electrode localization and diffusion MRI

Co-registering DBS electrode locations to a standard stereotactic space can be a powerful method to explore several key questions [211]. First, signatures from electrophysiological recordings can be mapped to anatomical space. Elevated local field potential activity in the theta band recorded from GPi-DBS electrodes correlates with symptom severity in CD [212] and this localizes to the posterolateral GPi.

Although much of the data are from rodent and non-human primate models, evidence including recordings from DBS patients suggests multiple changes in GPi neuronal physiology: lower firing rates, firing patterns that are less tonic and more irregular and bursty, increased oscillatory power in delta (1–3 Hz) and theta (3–8 Hz) ranges, and broadened somatosensory receptive fields, especially for symptomatic body regions (as reviewed in a proposed box-and-arrow network model of dystonia pathophysiology [213]).

Second, electrode localizations could help identify an optimal stimulation site (“sweet spot”). A multi-center study of 87 patients linked best outcomes to stimulation sites in the posterolateral GPi and, more precisely, its ventral border [214]. While considering this location as an optimal spot, the distribution of optimal contacts across this large cohort varied widely, suggesting subtype, pathology, and somatotopic symptom expression were important predictors of optimal DBS response. In other words, there may not be one optimal DBS target for all patients with dystonia.

Third, electrode localizations could also link treatment outcomes to distributed brain networks. In the past and in other diseases, tractography derived from dMRI has been used to associate DBS stimulation sites with structural brain networks [215–219]. However, tractography in the pallidal region is problematic because of its proximity to the internal capsule. Cortical input to the pallidum is known to traverse mainly through the striatopallidofugal bundle [220]. However, when seeding connections from the pallidum using dMRI based tractography, many results include the internal capsule as a false positive connection [221]. A potential solution to this problem was introduced by a basal ganglia atlas that had not been constructed based on tractography but on expert anatomical knowledge [222]. Pathways included in this resource should be free from false-positive connections, and all included tracts will match our current anatomical knowledge.

**FIGURE 7:**
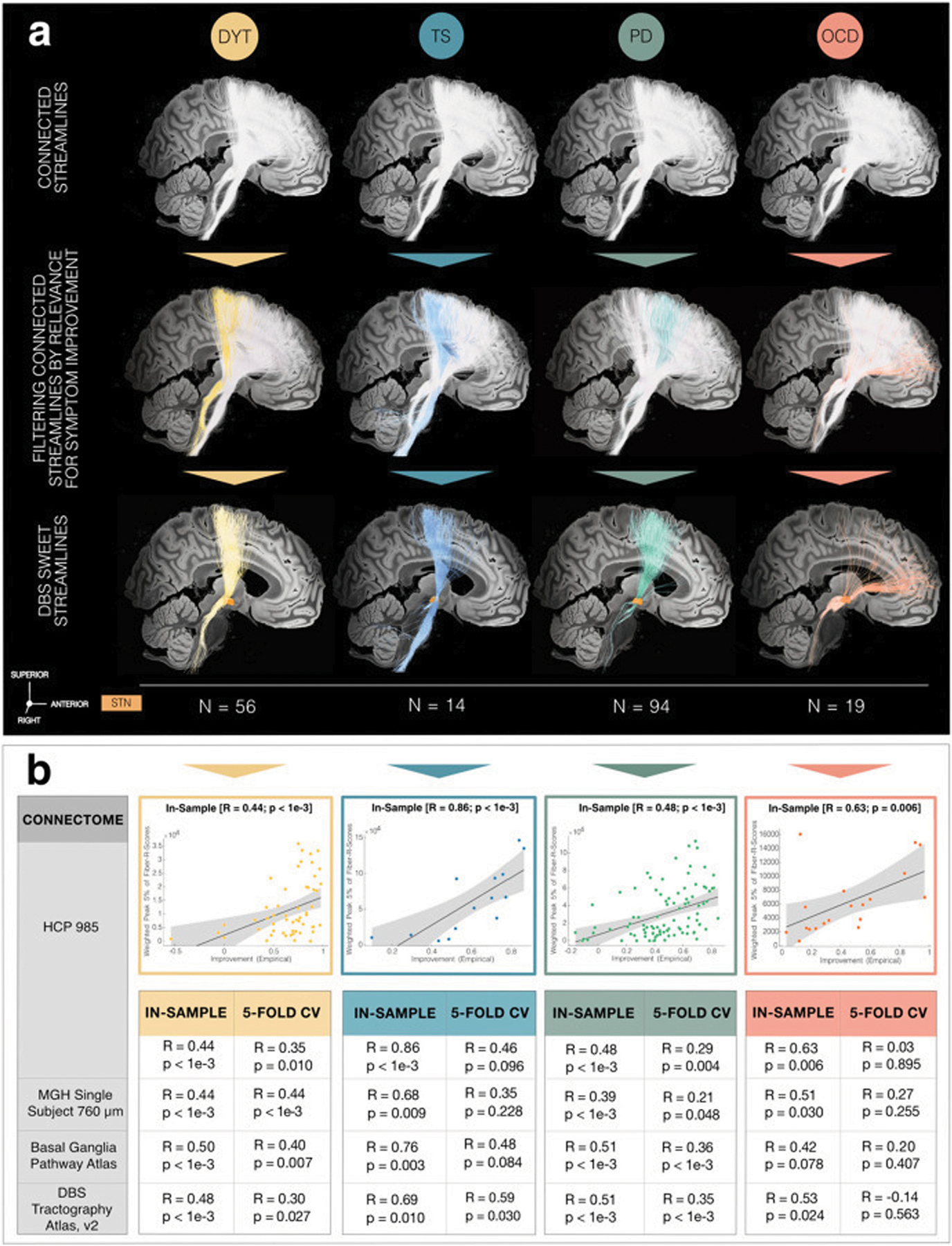
Sweet streamline models in the context of bilateral STN DBS implants. (a) Sweet streamlines (n = 56; peak R = 0.36) associated with beneficial stimulation outcomes were filtered from a population-based group connectome. The top row demonstrates the set of connections (in white) seeding from stimulation volumes across patients. Among these plain connections, only those were isolated via DBS Fiber Filtering (middle row) whose modulation was Spearman’s rank correlated with clinical outcomes (bottom row). Sweet streamlines are displayed in thresholded and binarized fashion. Results are shown against a sagittal slice (x = −5 mm) of the 7T MRI *ex vivo* 100-μm human brain template, in conjunction with a three-dimensional model of the right STN in template space from the DISTAL atlas, version 1.1. (b) In-sample correlations and 5-fold cross validations are reported for models informed on normative connectomes. Plots in the top row represent the fitting of a linear model to determine the degree to which the overlap of E-field magnitudes with selected HCP 985 Connectome sweet streamlines explains variance in empirical clinical outcome across the cohort, as calculated using Spearman’s correlation (two-sided tests). The magnitude of E-field overlap with sweet streamline models in this analysis is expressed as weighted peak 5% of Fiber R scores under each E-field, averaged across bilateral scores per patient. Gray shaded areas indicate 95% confidence intervals. Reproduced with permission from [5].

This detailed atlas was applied to DBS electrode localizations in 80 patients from five institutions to study networks associated with optimal response in cervical and generalized dystonia [223]. While this study confirmed that optimal stimulation sites mapped to the posterolateral somatomotor region of the GPi, it provided evidence for differential treatment mechanisms in cervical vs. generalized dystonia. Namely, response in CD mapped to pallidofugal fibers that projected radially into the internal capsule, along the main axis of the basal ganglia, such as the comb system of Edinger [224]. In contrast, optimal response in generalized dystonia mapped to pallidothalamic bundles such as the ansa and lenticular fasciculi. While projections of both systems are known to reunite in the thalamus, the finding could motivate differences in networks associated with cervical and generalized forms of dystonia. This overall approach of using DBS treatment outcomes to make inferences about networks implicated in dystonia has also been extended to STN-DBS (see Figure 7) [5]. Recently, a larger study was carried out to elucidate optimal stimulation sites and networks in subthalamic DBS for dystonia [206]. While axial forms of dystonia (such as cervical and truncal phenotypes) were best treated by directing the electrical field to the ventral oral posterior nucleus of the thalamus (and cerebellothalamic circuitries), appendicular forms were best treated when stimulating the subthalamic nucleus proper (and basal ganglia circuitries).

In sum, these three examples show the unique potential and insights gained from studies that combine precise DBS electrode reconstructions with tractography from dMRI and can compare electrophysiology, clinical effects and involved networks on a group level [225].

### Simultaneous recordings in GPi and thalamus in pediatric movement disorders

Dystonia is a prominent symptom of many pediatric movement disorders. To refine DBS targeting in pediatric movement disorders with heterogeneous distributions of CNS pathology, a protocol was developed using temporary depth electrodes at multiple candidate sites. Recording and test stimulation are performed over 5 days in a neuromodulation monitoring unit (NMU) with the child awake and able to participate in usual daily activities [226]. Subsequently, a total of four permanent DBS leads are implanted, usually in a combination of pallidal and thalamic targets [226]. This procedure raised the success rate in children with secondary dystonia from 50% to greater than 90%, and it expands the potentially effective targets. Diagnoses include secondary dystonia, primary dystonia, and Tourette syndrome. Only one of the 33 children did not proceed to permanent electrode implantation due to lack of an effective target [227].

These recordings yield new insight into DBS mechanisms for dystonia and related movement disorders. Stimulation-evoked potentials captured simultaneously across depth electrodes at multiple DBS frequencies reveal inter-regional connectivity and the spatiotemporal spread of stimulation. Comparison of correlations in spontaneous brain activity with the evoked potential shapes suggests that the DBS signal propagates at least partly along physiological pathways, enabling frequency-dependent maps of orthodromic and antidromic propagation useful for parameter selection.

Clinical results up to 5 years after implantation in children with secondary dystonia suggest that stimulation in the optimal thalamic target can almost completely alleviate the hyperkinetic component dystonia, whereas stimulation in the optimal target in GPi only partly alleviates the hypertonic component. This observation suggests that the mechanism of the hyperkinetic and hypertonic components may be different, and support symptom-specific target selection in pediatric cases.

Unexpectedly, both GPi and thalamic regions are relatively quiet at rest and increase their activity with attempts at voluntary movement [226, 227]– opposite the typically high resting GPi activity in Parkinson’s disease and in healthy non-human primates. Because GPi outputs inhibit thalamic targets, an excitatory drive to thalamic targets has been suggested, most likely arising from cortical glutamatergic efferent pathways back to thalamus. This supports a model in which the basal ganglia normally modulate and select activity in thalamocortical loops, and decreased firing in GPi leads to failure of modulation and selectivity. This could provide an explanation for both hypertonia due to excessive drive to motor cortices, as well as hyperkinetic movements due to failure of inhibition of unwanted thalamocortical dynamics. Further studies are needed to determine the mechanism by which stimulation in GPi or thalamus can selectively ameliorate these different components of dystonia.

The specific network functions of thalamic relays between cerebellum and striatum [228, 229], and indeed even different thalamic motor nuclei (i.e., Voa/Vop and VIM), still need to be elucidated and may play an important role in dystonia. In two adolescents with dystonia secondary to cerebral palsy, compared to a Tourette patient without dystonia, there was a drawing task-related increase in magnitude of activity in the GPi and Vim nucleus of the thalamus [230]. There was no such difference in other thalamic nuclei. Because GPi and Vim are the primary nodes in BG and cerebellar output pathways, respectively, it implicates both pathways in the altered motor control evident in this form of dystonia. A follow-on study supported the higher activity in the GPi, as well as stronger coupling from STN to GPi than from GPi to STN [231].

In pediatric dystonia, benzodiazepines reduce BG and thalamus activity and the efficacy of transmission between them [232], so future studies also need to carefully control for the influence of oral medications.

### Oscillopathies

The theoretical foundation for network models of movement disorders–including Parkinson’s disease as well as dystonia–now incorporates an “oscillatory synchronization” framework, the idea that abnormal synchronization of neuronal populations underlies specific signs and symptoms across brain disorders. One emerging concept is that the extent to which DBS attenuates pathological synchrony serves as a key biomarker of therapeutic efficacy [23, 233–235]. This principle, established in modeling motor fluctuations in Parkinson’s disease [236], is now being applied to dystonia.

Local field potentials and electrocorticography provide sensitive measures of oscillatory synchronization (see EEG section). Theta band (4–8 Hz) oscillatory activity within motor networks of the basal ganglia and cortex is associated with adult-onset CD [212]. Recently identified cortical gamma band oscillations (60–80 Hz) may represent another signature of dystonia [237]. Characterizing these rhythms is directly informing therapy, enabling identification of stimulation paradigms and parameters that normalize exaggerated oscillatory patterns. Current sense-and-stimulate devices could support even richer network analyses if they allowed recording with higher channel counts and could be attached to a wider variety of leads tailored for different recording sites.

## Non-invasive brain stimulation (NIBS)

The vast majority of dystonia patients do not undergo DBS surgery. Non-invasive brain stimulation (NIBS) methods provide less direct but still meaningful ways to modulate neural activity. Although generally limited to targeting only superficial structures like the cortex, NIBS can influence wider interconnected networks, including deeper structures, as demonstrated by combined neurophysiological and imaging studies showing widespread changes [238, 239].

Because dystonia pathophysiology involves decreased inhibition across multiple levels of the nervous system leading to co-contraction of agonist and antagonist muscles, distorted digit representation associated with loss of surround inhibition, and possibly excessive plasticity, most NIBS studies have aimed to reduce cortical excitability, which has been hypothesized to lead to increased inhibition and reduced abnormal plasticity. Accordingly, prior work has used rTMS or low-intensity TES such as tDCS to target nodes in the dystonia network, such as the motor cortex, premotor cortex, SMA, or cerebellum.

### Transcranial magnetic stimulation (TMS)

**FIGURE 8:**
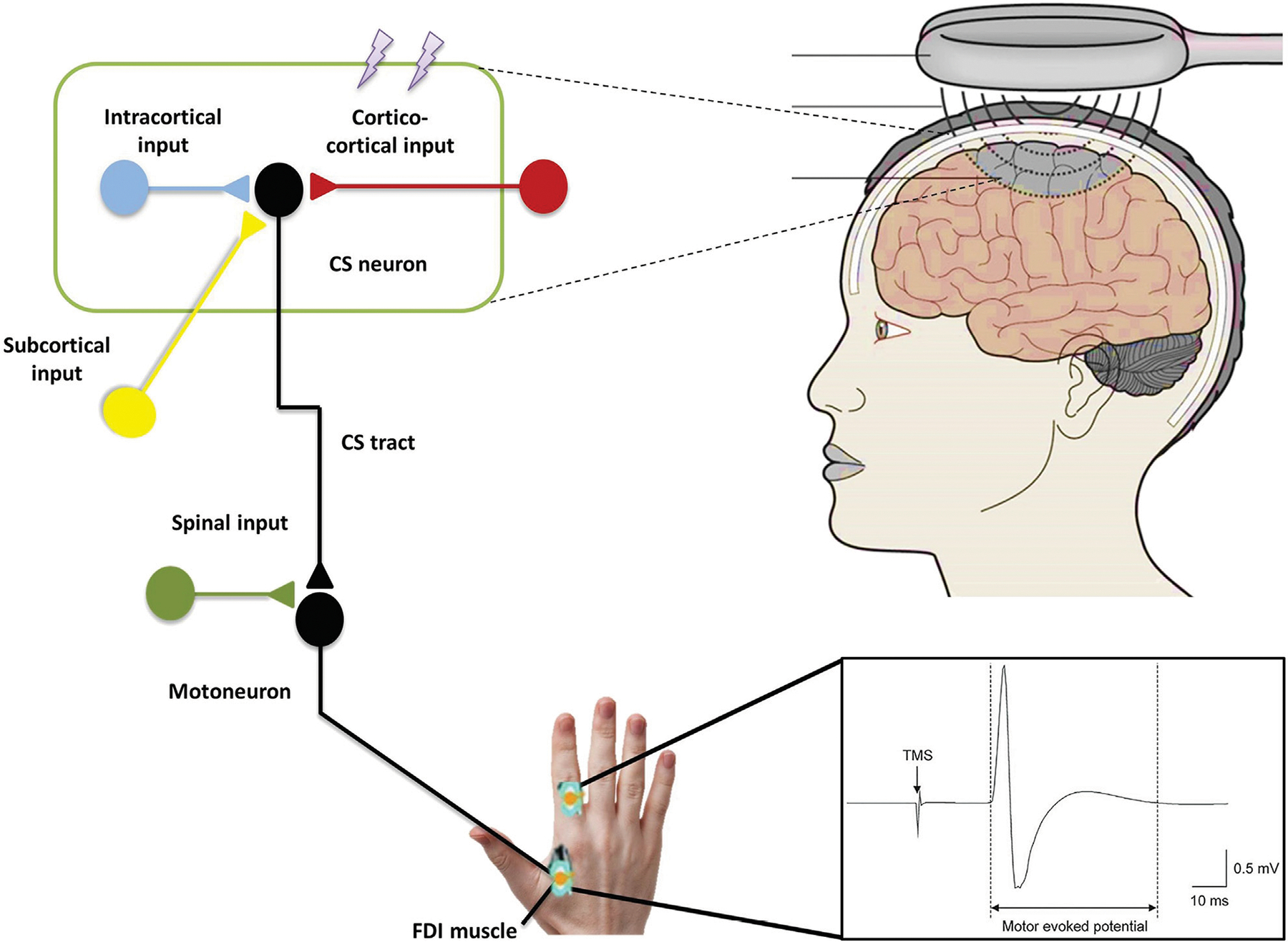
Schematic representation of TMS demonstrating the magnetic field generated with the magnetic coil placed over the hand area of the primary motor cortex. This, in turn, induces electrical current to activate cortical circuits (lightning bolts indicating the electromagnetic pulses) leading to activation of corticospinal neurons and subsequently alpha motor neurons in the spinal cord that innervate the muscle of interest, e.g., first dorsal interosseous muscle (FDI). This leads to motor evoked potential (MEP) recorded with surface EMG. Reproduced with permission from [297].

Many studies in focal dystonia have used transcranial magnetic stimulation (TMS), the most widely used form of non-invasive brain stimulation. It can be applied with many different protocols, the simplest involving motor cortex stimulation and measurement of corresponding muscle activation (see Figure 8). Repetitive TMS (rTMS) is commonly used to induce plasticity. Most rTMS studies in dystonia employ designed inhibitory protocols such as low frequency (~1 Hz) rTMS [17] or continuous theta burst stimulation (cTBS) [240]. FHD is the most studied condition. Low frequency rTMS over the primary motor cortex [241] and premotor cortex [242, 243] improves writing in patients with FHD. In CD, one study testing several cortical sites found that a single session of 0.2 Hz rTMS to dorsal premotor cortex and motor cortex stimulation produced the greatest reduction in dystonia [244], while 10 sessions of bilateral cerebellar cTBS reduced CD severity relative to sham stimulation [245]. In FHD and CD, a single session of repetitive cerebellar stimulation produced distinct immediate post-effects on cortical plasticity: cerebellar regulation of cortical plasticity was lost in FHD, but preserved in CD [246]. In CD, neck proprioceptive inputs may modulate the relationship between cerebellar output and cortical plasticity [246]. In FHD, loss of cerebellar control over sensorimotor plasticity correlated with impaired adaptive reaching [247]. In BSP, low frequency rTMS to the anterior cingulate cortex has yielded promising results [248]. In summary, inhibitory rTMS targeting premotor cortex, motor cortex, and cerebellum appear potentially beneficial for FHD and CD, whereas the anterior cingulate cortex is a promising target for BSP. Larger randomized controlled trials are still needed.

Future TMS studies in dystonia should control for pharmacologic state. In a study of FTSD with 24 participants, Zolpidem flattened rest and active input/output curves and reduced ICF compared to placebo [92]. BoNT also influences central physiology and therefore the response to TMS. In general, BoNT decreases sensorimotor activation during voluntary movements [249]. Electrophysiological evidence from TMS and reflex studies suggests BoNT-related plasticity in cortex and brainstem, respectively [250]. These plastic changes may persist, as clinical observations indicate lasting modifications of dystonic motor features beyond individual BoNT cycles.

FHD is also associated with plasticity–as measured using TMS in with a paradigm known as paired associative stimulation–that is excessive [251, 252], and abnormally regulated [253]. However, the findings remain controversial because some studies did not find excessive plasticity in dystonia [254]. Nevertheless, the complex longitudinal dynamics of various types of plasticity in dystonia, and the ability of TMS to measure and modulate plasticity at a macroscopic level, make plasticity an important direction for future research with TMS.

### Transcranial electrical stimulation (TES)

TES encompasses both tDCS and tACS. A study using cortical cathodal (excitatory) tDCS over motor cortex found no benefit in FHD [255], whereas anodal tDCS of the ipsilateral cerebellum produced conflicting results [254, 256]. In musician’s dystonia, improvements have been reported with cathodal tDCS to the motor cortex of the affected side and anodal tDCS to the unaffected side combined with motor training [257] or with bilateral parietal (cathode left, anode right) tDCS [258]. Although no formal studies exist in CD, case reports describe benefit from bilateral anodal cerebellar tDCS [259] and bilateral motor cortical 15 Hz tACS [260]. Overall, evidence for tDCS or tACS in dystonia remains preliminary, and further studies across different subtypes are needed.

### Transcranial ultrasound stimulation (TUS)

Low-intensity TUS is a novel NIBS method offering greater focality and penetration depth than other NIBS modalities. This is particularly important for dystonia, as several of the regions implicated are deeper, subcortical structures. Human studies show stimulation duration-dependent reductions in cortical excitability during the application of TUS (the “online” effect) [261]. Plasticity or offline effects have also been demonstrated. In non-human primates, fMRI demonstrated that 40 s of TUS to the frontal polar cortex or SMA altered functional connectivity between each site and their normal “connectional fingerprint” – the cortical areas with which they normally show connectivity as determined by BOLD correlations, e.g., for SMA it is primarily M1, superior parietal lobe, and middle cingulate cortex–for up to 60 min [262]. In humans, 80 s of TUS delivered in a theta burst pattern increased cortical excitability for at least 30 min [263]. In Parkinson disease and dystonia, recordings from DBS electrodes in the GPi showed that TUS can effectively modulate GPi activity, producing protocol-specific changes in neural activity [264]. Collectively, these findings position TUS as a promising non-invasive neuromodulation approach for dystonia.

### Integrating brain imaging and NIBS: toward multimodal, personalized noninvasive neuromodulation

Dystonia is a multifaceted condition; therefore, multimodal approaches that integrate neuroimaging and neurophysiology data into a unified pathophysiological framework offer a logical path toward deeper understanding and improved treatment. Within this context, NIBS techniques provide valuable tools for probing brain activity, elucidating mechanisms, and identifying novel therapeutic targets. In particular, TMS and TES can be combined with electrophysiology or neuroimaging to determine: 1) *where* to stimulate, by tailoring target regions to each patient’s individual anatomy or functional fingerprint; 2) *how* to personalize stimulation parameters (e.g., intensity, frequency) based on individual connectomic and biophysical models using structural and fMRI data, and 3) *when* to deliver stimulation by employing closed-loop, feedback-triggered paradigms guided by online measures such as EEG.

The optimal neuroimaging technique depends on the intervention’s objective. Structural MRI (T1- or T2-weighted) is highly effective for anatomical targeting, but combining it with metabolic (e.g., PET) and functional modalities (e.g., fMRI, ASL) has become standard practice for target selection [265]. Recent advances in hardware now enable modulation of neural *circuits* rather than isolated cortical areas, allowing simultaneous engagement of multiple network nodes and even interaction between networks [266]. This can be accomplished, for instance, with multicoil TMS, including cortico-cortical paired-associative-stimulation paradigms that deliver semi-synchronous stimulation to two brain regions [267]. Because this approach relies on Hebbian spike-timing–dependent plasticity, tuning it to circuit timing–by integrating diffusion imaging [268] and EEG–should permit measurement and modulation of network-level dynamics relevant to dystonia pathophysiology. Similarly, multichannel TES montages can focally stimulate specific cortical targets and simultaneously stimulate different areas belonging to the same or different networks to probe their dynamic interplay [269]. Recently developed biophysical modeling algorithms derive features from individual neuroimaging data to create realistic 3D head models and simulate stimulation-induced electric field distributions induced by TES or TMS [270, 271]. Such personalized models not only improve field control but also allow optimization of stimulation parameters in advance, enhancing precision and efficacy.

**FIGURE 9:**
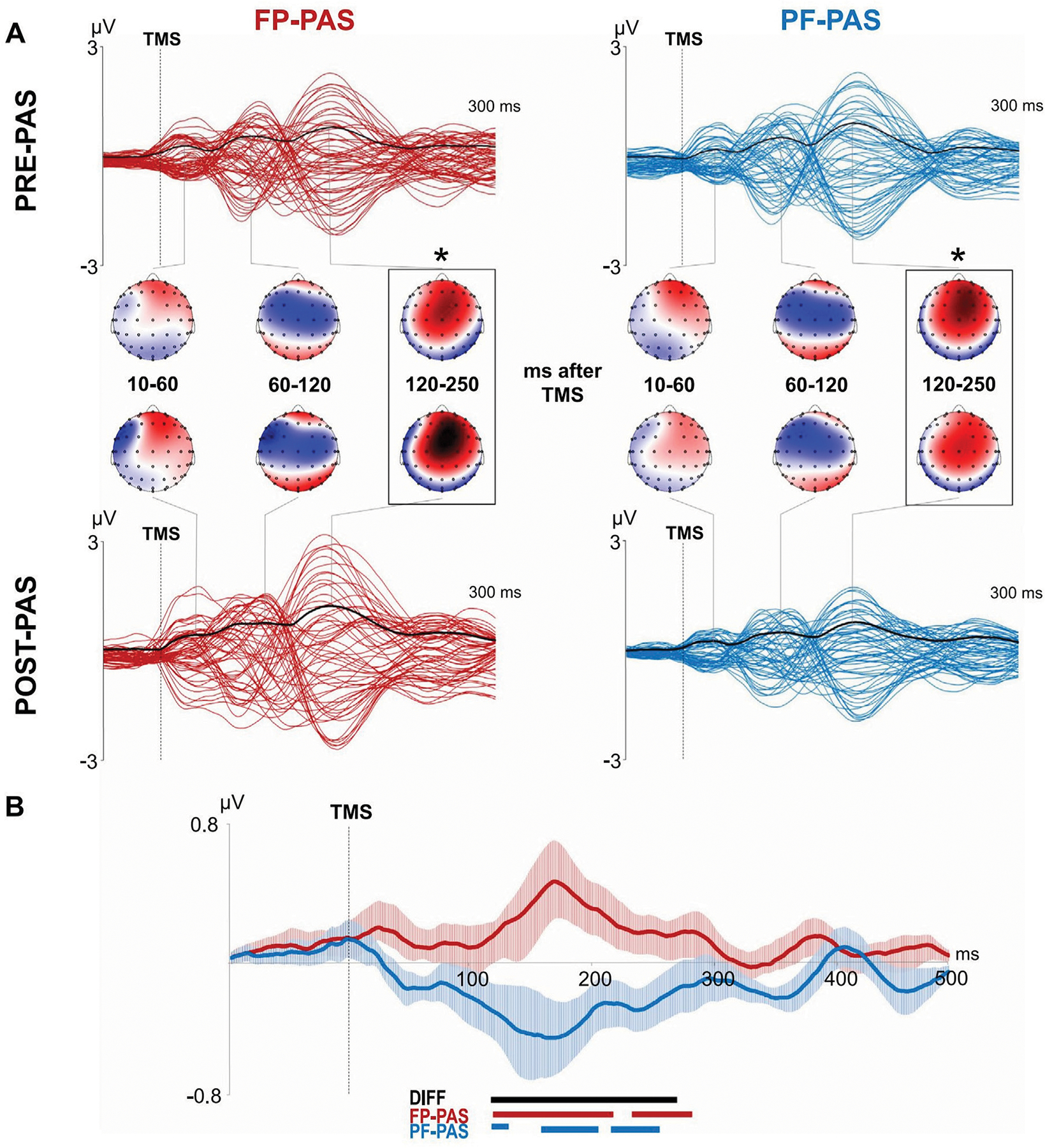
Network-level spike-timing-dependent plasticity (STDP) demonstrated with PAS TMS and EEG-based evoked potentials in DLPFC in healthy volunteers. Red: stimulation in DLPFC before PPC (“FP-PAS”). Blue: stimulation in PPC before DLPFC (“PF-PAS”). (A) Evoked potentials before (“PRE”) and after (“POST”) PAS, including per-trial time series (“butterfly plots”) and average time-windowed spatial distributions. Asterisks indicate significant differences (p < 0.05). (B) Global mean field power differences (POST-PRE), with thick lines underneath showing periods of statistically significant (p < 0.025) divergence and the bidirectionality of the induced plasticity. Reproduced with permission from [279].

Multimodal NIBS can also be delivered online by combining TES or TMS with neurophysiology recordings such as scalp EEG. Simultaneous TMS-EEG paradigms allow measurement of the brain’s real-time response to direct perturbation, enabling study of causal interactions between regions with high temporal resolution and providing insight into effective connectivity, cortical inhibition/excitation, and plasticity [272]. Macroscopic, network level forms of spike-timing dependent plasticity can be induced using two-site TMS and the effects quantified with evoked potentials in the EEG (see Figure 9) [273]. The combination of TES-EEG methods can increase the temporal precision of TES manipulations and enable brain state-dependent modulation. In closed-loop tACS-EEG protocols, phase and amplitude of ongoing brain activity are used to automatically adjust stimulation parameters and maximize entrainment of neural activity. This approach has been used, for example, to enhance slow-wave sleep and memory consolidation [274, 275] and could be adapted to target pathological oscillations in dystonia in real-time.

In summary, NIBS paradigms benefit substantially from integration with imaging methods, providing extensive information about cortical functional dynamics with high temporal (e.g., EEG) and spatial (e.g., MRI) resolution. However, a comprehensive review of NIBS studies across different dystonia subtypes [276] concludes that the results to date remain inconclusive. A likely reason is that most studies have targeted only a single stimulation site–typically somatosensory cortex, primary motor cortex, dorsal premotor cortex, or cerebellum [9, 277] – rather than addressing network-level dysfunction. Supporting this view, a recent study demonstrated top-down causal alterations of functional connectivity within the sensorimotor network in isolated focal dystonia [135]. Future work should therefore prioritize personalized multimodal stimulation protocols designed to influence both within-network and between-network dynamics.

## Computational models

### The brainstem and the neural integrator model

A key motivation for considering brainstem dysfunction in dystonia came from elegant clinical observations of head movements in CD [278]. In some patients, rotating the head away from the clinical null position toward a desired target is followed by an involuntary slow drift back towards the null, then a faster corrective movement toward the target. This pattern resembles gaze evoked nystagmus, which occurs when cerebellar feedback to the oculomotor neural integrator is impaired [279]. This prompted the question of whether an analogous neural integrator exists for head movements and whether it is dysfunctional in CD. Experimental work in animals points to a midbrain region–the interstitial nucleus of Cajal (INC) – as having properties consistent with a head-movement neural integrator [280]. This hypothesis is compelling for two reasons. First, it shifts attention to the brainstem, where inputs from a range of neuroanatomical locations and sensory modalities converge on the neural integrator. The neural integrator hypothesis can, therefore, accommodate the considerable diversity of findings in the literature; malfunction in any one of the inputs or the integrator itself could result in the abnormal neural control of head movements. Second, it yields testable predictions; for example, cerebellar and proprioceptive inputs to the INC become potential targets for central or peripheral neuromodulation.

Beyond the neural integrator model and the INC, three additional points about the brainstem are noteworthy: 1) although most of INC’s connections are with brainstem, spinal cord, and cerebellar regions, the PPN is another key integrative brainstem nucleus with reciprocal connections to BG and thalamus, providing a more direct interaction with BGTC loops heavily implicated in dystonia, 2) a theoretical framework based on rodent work implicates descending projections from basal ganglia to brainstem circuits in BSP [111], and 3) brainstem nuclei are seldom mentioned in lists of brain regions involved in dystonia networks, likely because they are difficult to delineate in standard neuroimaging. Higher field strength MRI should begin to address this limitation. Future dystonia network models should therefore incorporate these and other brainstem nodes.

### The virtual brain

Because dystonia seems to involve complex brain networks involving many regions, a potentially fruitful way to integrate and better understand pathophysiological data from many modalities is with computational simulations of those networks. For example, neuroimaging data can be merged with dynamic mean-field models to create large-scale brain simulations using a neuroinformatics platform such as The Virtual Brain (TVB; [281]). It is open source, written in Python, and includes a graphical interface to support usability. TVB is model agnostic: users can select from a library of mean-field models, ranging from simple oscillators to more complicated neural population models. In addition, the open-source framework allows users to add their own hypothesized model. Outside of dystonia, several applications of TVB have been demonstrated. Although recently adapted for mouse studies [282], TVB is most commonly used to model empirical human data (e.g., fMRI or EEG), to demonstrate how structural connectivity and local dynamics jointly shape intrinsic, resting state activity [283]. Initial clinical applications focused on stroke, where patient-specific models showed that local excitability predicted physiotherapy-related motor recovery. In epilepsy, development of the Epileptor model [284] enabled prediction of seizure focus location and is now being tested in a national clinical trial for clinical-decision support [285]. TVB has also been applied to dementia, where model parameters outperformed standard neuroimaging metrics in predicting cognitive performance across the disease spectrum [286]. More directly relevant to dystonia, recent work integrated detailed models of the basal ganglia in TVB to simulate DBS effects [287], demonstrating renormalization of circuit dynamics after stimulation and illustrating the potential to personalize stimulation parameters and target selection. TVB therefore represents a promising computational tool for investigating and treating pathophysiological brain networks in dystonia. Regardless of the specific modeling framework, the close coordination of modeling and experiments would inform each other in an iterative loop that facilitates progress in how we come to understand brain network pathophysiology in dystonia.

## Discussion

### Future imaging studies

Future dystonia research with all the imaging modalities should heed lessons learned from meta-analyses, which commonly include a critical review of methodological details in past studies and make corresponding recommendations for maximizing the informativeness of future imaging experiments [40]. Relatedly, future imaging studies would also benefit from larger-sized cohorts and adopting ongoing advances in analytics. As in neuroscience more broadly, research into dystonia network pathophysiology should incorporate algorithmic advances from the broader field of network science [288]. As but one example, functional connectivity gleaned from rs-fMRI can benefit from widely used [135] and emerging [289] methods to infer causality in the networks. Yet, attention to rigor in quality control plays a critical role for interpretation of findings [84] and improved consistency would enhance reproducibility and facilitate meta-analyses.

### Advances in DBS

When there is clinical justification to do so, DBS-associated recordings should take advantage of multiple recording sites, ideally simultaneously. In parallel, advances in DBS technology–whether via adaptive programming, coordinated reset stimulation protocols, or, ultimately, greater cell and circuit specificity–may provide not only greater treatment efficacy but also entirely new insights into the network pathophysiology of dystonia [23].

### Vagus nerve stimulation (VNS)

VNS using an implantable device is an approved treatment for drug-resistant epilepsy and depression. When paired with rehabilitation, VNS improves upper limb motor function after ischemic stroke [290]. The vagus nerve can also be stimulated non-invasively via the outer ear, which receives cutaneous supply by the auricular branch of the nerve [291], or percutaneously in the neck–a method that has shown promise for treating freezing of gait in Parkinson’s disease [292]. Given these findings, and the vagus nerve’s indirect influence on prefrontal cortical and cerebellar regions through other brainstem nuclei, VNS–possibly combined with rehabilitation–may represent a potential therapy for dystonia.

### Longitudinal dynamics

Most studies of network pathophysiology in dystonia are either cross-sectional or represent a small number of points in time (e.g., pre-/post-treatment). Although it would add an additional dimension to an already complex enterprise, evaluating how the network pathophysiology changes over longer time scales would strengthen understanding of the natural history of the disorder. If we had a better understanding of this process, it could provide a foundation for developing disease-modifying therapies. As an important subset of this, developmental aspects of dystonias, especially for but not limited to childhood-onset dystonias, would benefit from investigations into how the brain networks implicated in dystonia develop [293].

### Network implications of over-trained motor patterns

Our understanding of dystonia at the molecular level has expanded considerably over the past two decades [294]. Yet many dystonia subtypes, particularly focal forms, are also likely shaped by environmental influences [295, 296]. Task-specific dystonias, for example, have been linked to multiple environmental risk factors [297], and such features can help infer why motor control and skill reproduction break down under certain conditions [134, 298]. A motor-control framework is valuable not only because it clarifies mechanism, but also because it provides a shared language for discussing impairments with patients and for developing targeted interventions. One such intervention stems from the observation that patients appear trapped in an over-trained dystonic motor pattern and behavioral interventions that stochastically inject variability into movement repetitions during retraining can disrupt this pattern [299]. A substantial proportion of patients have returned to professional performance after such an intervention [300]. The neuroanatomical network underlying task-specific dystonia likely spans a broad sensorimotor hierarchy and will vary depending on whether one is trying to find the network responsible for vulnerabilities endowed by certain risk factors, the dystonia motor pattern itself, or the clinical trajectory of the disorder. That said, associative higher-order regions such as the premotor and parietal cortex emerge repeatedly across multiple research approaches [136, 298]. This task-specificity has motivated proposals for therapeutic brain-computer interfaces (BCIs) that enable patients to modulate pathological brain activity so that it more closely resembles activity during an asymptomatic task, with the expectation of symptom reduction [22]. A clinical trial of such a BCI intervention in LD patients (NCT04421365) is currently underway.

### Can we target the dystonia network through a common dystonic phenotype?

As molecular insights expand, it is worth asking whether we have neglected the features of the dystonic phenotype itself [301]. Can dystonia as a phenotype–defined by its characteristic motor features rather than by etiology (e.g., DYT-TOR1A) or subtype (e.g., adult-onset focal dystonia) – be investigated as a meaningful entity in its own right? The dystonic phenotype has reliable clinical features, recognizable kinematic characteristics, and many effective interventions act at the systems control level rather than the molecular level. For example, DBS provides substantial benefit in dystonia, yet its mechanism is comparatively coarse, most likely modulating activity or excitability of target regions rather than injecting normal patterns of neural activity or selectively modifying abnormal patterns [302]. This raises the question of whether shared kinematic signatures could help characterize dysfunctional networks in dystonia, analogous to how oscillatory movement features serve as teaching signals for adaptive neuromodulation of tremor [303, 304]. Likewise, neuro-physiotherapy engages the dystonic network in its entirety, and an emerging evidence base supporting its use for specific motor control axes within specific subsets of dystonia [305, 306]. Until targeted molecular therapies are available, approaches that act on common features of the dystonic phenotype may represent an effective way to both probe and modulate the underlying network.

### Tremor and dystonia

Tremor is recognized as an important aspect of dystonia [6, 307]. Because of increasing interest in tremor in dystonia, and because the associated terminology continues to evolve, we consider this an important topic for future research into the network pathophysiology of dystonia. The term “dystonic tremor” has been widely used [308, 309], but the consensus from a group of specialists in dystonia and tremor have suggested that this term has had variable interpretations and can be misleading [310]. They suggest that the term “tremor” should be reserved for movements that are rhythmic, and that, in the context of dystonia, repetitive movements that appear grossly arrhythmic should be called “jerky dystonia.” But precisely how rhythmicity is operationally defined remains unclear. In our present treatment, our descriptions of dystonia with tremor include the traditional, broadly defined dystonic tremor.

In general, dystonia with and without tremor share the same overall circuit pathophysiology, encompassing basal ganglia, cerebellum, and sensorimotor cortex [311]. However, dystonia with tremor seems to exhibit a stronger contribution from CbTC loops that might be more rhythmically engaged. In dystonia with tremor in the upper limb or head, there was increased volume of motor cortex and the same thalamic region that shows tremor-locked activity, and cerebellum-thalamic connectivity was positively correlated with tremor power [183]. In the context of LD, dystonic voice tremor patients exhibited additional abnormalities on fMRI in medial frontal gyrus, cerebellum, and posterior limb of internal capsule [312]. Compared to essential tremor, dystonic tremor patients exhibited greater reductions in functional connectivity between cortex, BG, thalamus, and cerebellum [161]. Single unit neuronal recordings during procedures previously used to ablate the INC for CD found firing properties that differed for CD with versus without tremor, for thalamic subregions receiving projections from either GPi or cerebellum [313]. The firing patterns in GPi may be more nuanced: CD with and without jerky tremor had similar firing patterns, but CD with sinusoidal tremor showed a different distribution of firing pattern properties [314, 315]. Interestingly, one study suggests that different types of tremor show different responses to non-invasive stimulation; tACS suppressed or enhanced tremor in a phase-dependent fashion for sinusoidal but not for jerky tremor, and for cerebellar but not motor cortical stimulation [316]. Collectively, the evidence to date suggests that networks involving the cerebellum play an important role in at least some types of tremor seen in dystonia.

### Functional dystonia

Although this review was inherently focused on organic dystonia, contemporary views of functional movement disorders, including functional dystonia, view it as having pathophysiology that can inform our understanding of dystonia more broadly defined. Organic and functional dystonias exhibit substantial overlap in their brain network abnormalities, including decreased cortical inhibition [317, 318]. But there are also several differences. Functional dystonia exhibited decreased volume of caudate, nucleus accumbens, putamen, and thalamus [319]. At rest, functional dystonia’s metabolic demands measured with PET were increased in the cerebellum and BG and decreased in motor cortex, a pattern opposite of that found in organic dystonia [320]. Functional dystonia also exhibited decreased functional connectivity between the right temporoparietal junction and a) bilateral sensorimotor cortex [321] and b) dorsal and rostral prefrontal cortex [322]. Given the role of the temporoparietal junction in comparing internal predictions of motor intentions with actual motor events, this might explain the altered sense of self-agency characteristic of functional dystonia. Functional dystonia also may be associated with altered emotional processing, because during emotional processing tasks functional dystonia patients exhibited decreased activation in right medial temporal gyrus, bilateral precuneus, and left insula [323]. A limited number of cases of functional dystonia patients receiving DBS showed GPi firing rates similar to organic dystonia [324]. Non-invasive brain stimulation over left dorsolateral prefrontal cortex alleviated symptoms in functional dystonia, including intermittent theta burst TMS [325] and anodal tDCS [326]. As with most research with organic dystonia, the study results are associations, and therefore cannot inform what is cause vs. effect in terms of brain network changes.

## Summary

In summary, there is a large and growing body of evidence that has begun to characterize dysfunctional networks in dystonia. The evidence comes from a multitude of modalities for measuring brain regional and network activity in humans. Across the vast literature on this topic, it is difficult to determine how much specific dystonia subtypes, tasks, and study designs variously contribute to the heterogeneity of results [22]. In general, there is convergent evidence implicating networks that include primary sensorimotor cortical areas, several nuclei in the BG, the thalamus, the cerebellum, and the brainstem. However, there is also evidence for numerous additional regions, primarily in the form of a variety of cortical areas beyond primary sensorimotor territories, such as premotor, supplementary motor, and parietal cortices.

There is a synergistic relationship between the research into these networks and the development of new and improved treatments. Naturally, the research into the networks informs new treatment development. But also measuring brain network activity in response to treatment, as well as during the process of implanting DBS electrodes, for example, can inform understanding of the dysfunctional networks. Ultimately, as our knowledge of the specific dysfunctions of the intricate networks involved in dystonia improves, it should, in turn, give rise to improved and personalized therapies, including oral drugs, BoNT, DBS, and NIBS.

## Glossary

ACh: Acetylcholine
ADSD: adductor spasmodic dysphonia
AES-SDM: anisotropic effect size-based signed differential mapping
BG: basal ganglia
BGTC: basal ganglia thalamocortical
BOLD: blood oxygen level-dependent
BoNT: botulinum neurotoxin
BSP: blepharospasm
CD: cervical dystonia
CbTC: cerebellothalamocortical
CT: computed tomography
cTBS: continuous theta burst stimulation
DBS: deep brain stimulation
dMRI: diffusion MRI
EEG: electroencephalography
FDG: fluorodeoxyglucose
FHD: focal hand dystonia
fMRI: functional MRI
fNIRS: functional near-infrared spectroscopy
FTSD: focal task-specific dystonia
GABA: gamma-aminobutyric acid
GABAA: GABA A (i.e., GABA receptor type)
GP: globus pallidus
GPi: globus pallidus, internal segment
ICA: independent components analysis
INC: interstitial nucleus of Cajal
LD: laryngeal dystonia
M1: primary motor cortex
MAN: manifesting
MEG: magnetoencephalography
MPTP: 1-methyl-4-phenyl-1,2,3,6-tetrahydropyridine
MRI: magnetic resonance imaging
NIBS: noninvasive brain stimulation
NM: non-manifesting
PET: positron emission tomography
PPN: pedunculopontine nucleus
rs-fMRI: resting state functional MRI
rTMS: repetitive TMS
S1: primary somatosensory cortex
SMA: supplementary motor area
STN: subthalamic nucleus
tACS: transcranial alternating current stimulation
tDCS: transcranial direct current stimulation
TES: transcranial electrical stimulation
TMS: transcranial magnetic stimulation
TUS: transcranial ultrasound
TVB: The Virtual Brain
VBM: voxel-based morphometry
VNS: vagus nerve stimulation

## References

[1] Hallett M Apraxia: the rise, fall and resurrection of diagrams to explain how the brain works. Brain (2015) 138(1):229–31. doi:10.1093/brain/awu302

[2] Disconnexion NG. Syndromes in animals and man. I. Brain (1965) 88:237–94. doi:10.1093/brain/88.2.237 5318481

[3] Geschwind N Disconnexion syndromes in animals and man. II. Brain (1965) 88:585–644. doi:10.1093/brain/88.3.585 5318824

[4] Hallett M, de Haan W, Deco G, Dengler R, Iorio R, Gallea C, Human brain connectivity: clinical applications for clinical neurophysiology. Clin Neurophysiol (2020) 131(7):1621–51. doi:10.1016/j.clinph.2020.03.031 32417703

[5] Hollunder B, Ostrem JL, Sahin IA, Rajamani N, Oxenford S, Butenko K, Mapping dysfunctional circuits in the frontal cortex using deep brain stimulation. Nat Neurosci (2024) 27(3):573–86. doi:10.1038/s41593-024-01570-1 38388734 PMC10917675

[6] Albanese A, Bhatia KP, Fung VSC, Hallett M, Jankovic J, Klein C, Definition and classification of dystonia. Movement Disord (2025) 40(7):1248–59. doi:10.1002/mds.30220 40326714 PMC12273609

[7] Middlebrooks EH, Domingo RA, Vivas-Buitrago T, Okromelidze L, Tsuboi T, Wong JK, Neuroimaging advances in deep brain stimulation: review of indications, anatomy, and brain connectomics. Am J Neuroradiology (2020) 41(9):1558–68. doi:10.3174/ajnr.A6693 PMC7583111 32816768

[8] Conte A, Defazio G, Mascia M, Belvisi D, Pantano P, Berardelli A. Advances in the pathophysiology of adult-onset focal dystonias: recent neurophysiological and neuroimaging evidence. F1000Research (2020) 9:67. doi:10.12688/f1000research.21029.2 PMC6993830 32047617

[9] Quartarone A, Hallett M. Emerging concepts in the physiological basis of dystonia. Movement Disord (2013) 28(7):958–67. doi:10.1002/mds.25532 23893452 PMC4159671

[10] Perlmutter JS, Tempel LW, Black KJ, Parkinson D, Todd RD. MPTP induces dystonia and parkinsonism: clues to the pathophysiology of dystonia. Neurology (1997) 49(5):1432–8. doi:10.1212/wnl.49.5.1432 9371934

[11] Mink JW, Thach WT. Basal ganglia intrinsic circuits and their role in behavior. Curr Opin Neurobiol (1993) 3(6):950–7. doi:10.1016/0959-4388(93)90167-W 8124079

[12] Latorre A, Rocchi L, Bhatia KP. Delineating the electrophysiological signature of dystonia. Exp Brain Res (2020) 238(7–8):1685–92. doi:10.1007/s00221-020-05863-2 32712678

[13] Jinnah HA, Neychev V, Hess EJ. The anatomical basis for dystonia: the motor network model. Tremor and Other Hyperkinetic Movements (2017) 7(0):506. doi:10.5334/tohm.383 29123945 PMC5673689

[14] van der Kamp W, Berardelli A, Rothwell JC, Thompson PD, Day BL, Marsden CD. Rapid elbow movements in patients with torsion dystonia. J Neurol Neurosurg and Psychiatry (1989) 52(9):1043–9. doi:10.1136/jnnp.52.9.1043 2795073 PMC1031738

[15] Nakashima K, Rothwell JC, Day BL, Thompson PD, Shannon K, Marsden CD. Reciprocal inhibition between forearm muscles in patients with writer’s cramp and other occupational cramps, symptomatic hemidystonia and hemiparesis due to stroke. Brain (1989) 112(3):681–97. doi:10.1093/brain/112.3.681 2731027

[16] Berardelli A, Rothwell JC, Day BL, Marsden CD. Pathophysiology of blepharospasm and oromandibular dystonia. Brain (1985) 108(3):593–608. doi:10.1093/brain/108.3.593 4041776

[17] Chen R, Wassermann EM, Caños M, Hallett M. Impaired inhibition in writer’s cramp during voluntary muscle activation. Neurology (1997) 49(4):1054–9. doi:10.1212/WNL.49.4.1054 9339689

[18] Ridding MC, Sheean G, Rothwell JC, Inzelberg R, Kujirai T. Changes in the balance between motor cortical excitation and inhibition in focal, task specific dystonia. J Neurol Neurosurg and Psychiatry (1995) 59(5):493–8. doi:10.1136/jnnp.59.5.493 8530933 PMC1073711

[19] Perlmutter JS, Mink JW. Dysfunction of dopaminergic pathways in dystonia. Adv Neurol (2004) 94:163–70. 14509670

[20] Tempel LW, Perlmutter JS. Abnormal vibration-induced cerebral blood flow responses in idiopathic dystonia. Brain (1990) 113(3):691–707. doi:10.1093/brain/113.3.691 2364264

[21] Rauschenberger L, Knorr S, Pisani A, Hallett M, Volkmann J, Ip CW. Second hit hypothesis in dystonia: dysfunctional cross talk between neuroplasticity and environment? Neurobiol Dis (2021) 159:105511. doi:10.1016/j.nbd.2021.105511 34537328

[22] Gill JS, Nguyen MX, Hull M, van der Heijden ME, Nguyen K, Thomas SP, Function and dysfunction of the dystonia network: an exploration of neural circuits that underlie the acquired and isolated dystonias. Dystonia (Lausanne, Switzerland) (2023) 2:11805. doi:10.3389/dyst.2023.11805 38273865 PMC10810232

[23] Gittis AH, Sillitoe RV. Circuit-specific deep brain stimulation provides insights into movement control. Annu Review Neuroscience (2024) 47(1):63–83. doi:10.1146/annurev-neuro-092823-104810 38424473

[24] Zeuner KE, Knutzen A, Granert O, Trampenau L, Baumann A, Wolff S, Never too little: grip and lift forces following probabilistic weight cues in patients with writer’s cramp. Clin Neurophysiol (2021) 132(12):2937–47. doi:10.1016/j.clinph.2021.09.010 34715418

[25] Thomsen M, Lange LM, Zech M, Lohmann K. Genetics and pathogenesis of dystonia. Annu Review Pathology (2024) 19:99–131. doi:10.1146/annurev-pathmechdis-051122-110756 37738511

[26] Bhatia KP, Marsden CD. The behavioural and motor consequences of focal lesions of the basal ganglia in man. Brain (1994) 117(4):859–76. doi:10.1093/brain/117.4.859 7922471

[27] Perlmutter JS, Raichle ME. Pure hemidystonia with basal ganglion abnormalities on positron emission tomography. Ann Neurol (1984) 15(3): 228–33. doi:10.1002/ana.410150303 6609680

[28] Corp DT, Greenwood CJ, Morrison-Ham J, Pullinen J, McDowall GM, Younger EFP, Clinical and structural findings in patients with lesion-induced dystonia. Neurology (2022) 99(18):e1957–e1967. doi:10.1212/WNL.0000000000201042 35977840 PMC9651464

[29] Gelineau-Morel R, Dlamini N, Bruss J, Cohen AL, Robertson A, Alexopoulos D, Network localization of pediatric lesion-induced dystonia. Ann Neurol (2025) 98:152–62. doi:10.1002/ana.27224 40059836 PMC12176519

[30] Gordon EM, Chauvin RJ, Van AN, Rajesh A, Nielsen A, Newbold DJ, A somato-cognitive action network alternates with effector regions in motor cortex. Nature (2023) 617(7960):351–9. doi:10.1038/s41586-023-05964-2 37076628 PMC10172144

[31] Dosenbach NUF, Raichle M, Gordon EM. The brain’s cingulo-opercular action-mode network (2024). doi:10.31234/osf.io/2vt79

[32] Xu J, Zhang X, Cheng Q, Zhang H, Zhong L, Luo Y, Abnormal supplementary motor areas are associated with idiopathic and acquired blepharospasm. Parkinsonism and Related Disorders (2024) 121:106029. doi:10.1016/j.parkreldis.2024.106029 38394948

[33] Corp DT, Joutsa J, Darby RR, Delnooz CCS, van de Warrenburg BPC, Cooke D, Network localization of cervical dystonia based on causal brain lesions. Brain (2019) 142(6):1660–74. doi:10.1093/brain/awz112 31099831 PMC6536848

[34] Simonyan K, Ludlow CL. Abnormal structure-function relationship in Spasmodic dysphonia. Cereb Cortex (2012) 22(2):417–25. doi:10.1093/cercor/bhr120 21666131 PMC3256408

[35] Shakkottai VG, Batla A, Bhatia K, Dauer WT, Dresel C, Niethammer M, Current opinions and areas of consensus on the role of the cerebellum in dystonia. The Cerebellum (2017) 16(2):577–94. doi:10.1007/s12311-016-0825-6 27734238 PMC5336511

[36] Lehéricy S, Tijssen MAJ, Vidailhet M, Kaji R, Meunier S. The anatomical basis of dystonia: current view using neuroimaging. Movement Disord (2013) 28(7): 944–57. doi:10.1002/mds.25527 23893451

[37] Draganski B, Thun-Hohenstein C, Bogdahn U, Winkler J, May A. Motor circuit’ gray matter changes in idiopathic cervical dystonia. Neurology (2003) 61(9): 1228–31. doi:10.1212/01.WNL.0000094240.93745.83 14610125

[38] Ramdhani RA, Kumar V, Velickovic M, Frucht SJ, Tagliati M, Simonyan K. What’s special about task in dystonia? A voxel-based morphometry and diffusion weighted imaging study. Movement Disord (2014) 29(9):1141–50. doi:10.1002/mds.25934 24925463 PMC4139455

[39] Obermann M, Yaldizli O, Greiff A, Lachenmayer ML, Buhl AR, Tumczak F, Morphometric changes of sensorimotor structures in focal dystonia. Movement Disord (2007) 22(8):1117–23. doi:10.1002/mds.21495 17443700

[40] MacIver CL, Tax CMW, Jones DK, Peall KJ. Structural magnetic resonance imaging in dystonia: a systematic review of methodological approaches and findings. Eur J Neurol (2022) 29(11):3418–48. doi:10.1111/ene.15483 35785410 PMC9796340

[41] Delmaire C, Vidailhet M, Elbaz A, Bourdain F, Bleton JP, Sangla S, Structural abnormalities in the cerebellum and sensorimotor circuit in writer’s cramp. Neurology (2007) 69(4):376–80. doi:10.1212/01.wnl.0000266591.49624.1a 17646630

[42] Garraux G, Bauer A, Hanakawa T, Wu T, Kansaku K, Hallett M. Changes in brain anatomy in focal hand dystonia. Ann Neurol (2004) 55(5):736–9. doi:10.1002/ana.20113 15122716

[43] Mantel T, Altenmüller E, Li Y, Meindl T, Jochim A, Lee A, Abnormalities in grey matter structure in embouchure dystonia. Parkinsonism and Relat Disord (2019) 65:111–6. doi:10.1016/j.parkreldis.2019.05.008 31147222

[44] Bianchi S, Battistella G, Huddleston H, Scharf R, Fleysher L, Rumbach AF, Phenotype- and genotype-specific structural alterations in Spasmodic dysphonia. Movement Disord (2017) 32(4):560–8. doi:10.1002/mds.26920 28186656 PMC5578762

[45] Gui H, Xiao P, Xu B, Zhao X, Wang H, Tao L, Machine learning models for diagnosis of essential tremor and dystonic tremor using grey matter morphological networks. Parkinsonism and Related Disorders (2024) 124:106985. doi:10.1016/j.parkreldis.2024.106985 38718478

[46] Wang ZY, Chen F, Sun HH, Li HL, Hu JB, Dai ZY, No reliable gray matter alterations in idiopathic dystonia. Front Neurol (2025) 16:1510115. doi:10.3389/fneur.2025.1510115 40098684 PMC11911186

[47] Huang X, Zhang M, Li B, Shang H, Yang J. Structural and functional brain abnormalities in idiopathic cervical dystonia: a multimodal meta-analysis. Parkinsonism and Relat Disord (2022) 103:153–65. doi:10.1016/j.parkreldis.2022.08.029 36100530

[48] Kshatriya N, Battistella G, Simonyan K. Structural and functional brain alterations in laryngeal dystonia: a coordinate-based activation likelihood estimation meta-analysis. Hum Brain Mapp (2024) 45(14):e70000. doi:10.1002/hbm.70000 39305101 PMC11415616

[49] Tarrano C, Wattiez N, Delorme C, McGovern EM, Brochard V, Thobois S, Visual sensory processing is altered in myoclonus dystonia. Movement Disord (2020) 35(1):151–60. doi:10.1002/mds.27857 31571302

[50] Simonyan K, Tovar-Moll F, Ostuni J, Hallett M, Kalasinsky VF, Lewin-Smith MR, Focal white matter changes in Spasmodic dysphonia: a combined diffusion tensor imaging and neuropathological study. Brain (2008) 131(2): 447–59. doi:10.1093/brain/awm303 18083751 PMC2376833

[51] Sondergaard RE, Rockel CP, Cortese F, Jasaui Y, Pringsheim TM, Sarna JR, Microstructural abnormalities of the dentatorubrothalamic tract in cervical dystonia. Movement Disord (2021) 36(9):2192–8. doi:10.1002/mds.28649 34050556

[52] Giannì C, Piervincenzi C, Belvisi D, Tommasin S, De Bartolo MI, Ferrazzano G, Cortico-subcortical white matter bundle changes in cervical dystonia and blepharospasm. Biomedicines (2023) 11(3):753. doi:10.3390/biomedicines11030753 36979732 PMC10044819

[53] Zhang J, Luo Y, Zhong L, Liu H, Yang Z, Weng A, Topological alterations in white matter anatomical networks in cervical dystonia. BMC Neurology (2024) 24(1):179. doi:10.1186/s12883-024-03682-4 38802755 PMC11129473

[54] Mahajan A, Stoub T, Gonzalez DA, Stebbins G, Gray G, Warner-Rosen T, Understanding anxiety in cervical dystonia: an imaging study. Movement Disorders Clinical Practice (2024) 11(8):1008–12. doi:10.1002/mdc3.14070 38747154 PMC11329561

[55] Gallea C, Balas M, Bertasi E, Valabregue R, García-Lorenzo D, Coynel D, Increased cortico-striatal connectivity during motor practice contributes to the consolidation of motor memory in writer’s cramp patients. NeuroImage: Clin (2015) 8:180–92. doi:10.1016/j.nicl.2015.04.013 26106542 PMC4473821

[56] Delmaire C, Vidailhet M, Wassermann D, Descoteaux M, Valabregue R, Bourdain F, Diffusion abnormalities in the primary sensorimotor pathways in writer’s cramp. Arch Neurol (2009) 66(4):502–8. doi:10.1001/archneurol.2009.8 19364935

[57] Berndt M, Li Y, Gora-Stahlberg G, Jochim A, Haslinger B. Impaired white matter integrity between premotor cortex and basal ganglia in writer’s cramp. Brain Behav (2018) 8(10):e01111. doi:10.1002/brb3.1111 30239158 PMC6192408

[58] Mantel T, Dresel C, Welte M, Meindl T, Jochim A, Zimmer C, Altered sensory system activity and connectivity patterns in adductor Spasmodic dysphonia. Scientific Rep (2020) 10(1):10179. doi:10.1038/s41598-020-67295-w PMC7311401 32576918

[59] Argyelan M, Carbon M, Niethammer M, Uluǧ AM, Voss HU, Bressman SB, Cerebellothalamocortical connectivity regulates penetrance in dystonia. J Neurosci (2009) 29(31):9740–7. doi:10.1523/JNEUROSCI.2300-09.2009 19657027 PMC2745646

[60] Vo A, Sako W, Dewey SL, Eidelberg D, Uluğ AM. 18FDG-microPET and MR DTI findings in Tor1a+/− heterozygous knock-out mice. Neurobiol Dis (2015) 73: 399–406. doi:10.1016/j.nbd.2014.10.020 25447231

[61] Sako W, Fujita K, Vo A, Rucker JC, Rizzo JR, Niethammer M, The visual perception of natural motion: abnormal task-related neural activity in DYT1 dystonia. Brain (2015) 138(12):3598–609. doi:10.1093/brain/awv282 26419798 PMC4840548

[62] Fujita K, Sako W, Vo A, Bressman SB, Eidelberg D. Disruption of network for visual perception of natural motion in primary dystonia. Hum Brain Mapp (2018) 39(3):1163–74. doi:10.1002/hbm.23907 29214728 PMC5807185

[63] Uluğ AM, Vo A, Argyelan M, Tanabe L, Schiffer WK, Dewey S, Cerebellothalamocortical pathway abnormalities in torsinA DYT1 knock-in mice. Proc Natl Acad Sci (2011) 108(16):6638–43. doi:10.1073/pnas.1016445108 21464304 PMC3081027

[64] Lerner RP, Niethammer M, Eidelberg D. Understanding the anatomy of dystonia: determinants of penetrance and phenotype. Curr Neurol Neurosci Rep (2013) 13(11):401. doi:10.1007/s11910-013-0401-0 24114145 PMC3883436

[65] Bianchi S, Fuertinger S, Huddleston H, Frucht SJ, Simonyan K. Functional and structural neural bases of task specificity in isolated focal dystonia. Movement Disord (2019) 34(4):555–63. doi:10.1002/mds.27649 30840778 PMC6945119

[66] Berman BD, Honce JM, Shelton E, Sillau SH, Nagae LM. Isolated focal dystonia phenotypes are associated with distinct patterns of altered microstructure. NeuroImage: Clin (2018) 19:805–12. doi:10.1016/j.nicl.2018.06.004 30013924 PMC6024227

[67] Tomić A, Agosta F, Sarasso E, Svetel M, Kresojević N, Fontana A, Brain structural changes in focal dystonia—what about task specificity? A multimodal MRI study. Movement Disord (2021) 36(1):196–205. doi:10.1002/mds.28304 32979238

[68] Fuertinger S, Simonyan K. Task-specificity in focal dystonia is shaped by aberrant diversity of a functional network kernel. Movement Disord (2018) 33(12): 1918–27. doi:10.1002/mds.97 30264427 PMC6309584

[69] Egger K, Mueller J, Schocke M, Brenneis C, Rinnerthaler M, Seppi K, Voxel based morphometry reveals specific gray matter changes in primary dystonia. Movement Disord (2007) 22(11):1538–42. doi:10.1002/mds.21619 17588241

[70] Carbon M, Kingsley PB, Su S, Smith GS, Spetsieris P, Bressman S, Microstructural white matter changes in carriers of the DYT1 gene mutation. Ann Neurol (2004) 56(2):283–6. doi:10.1002/ana.20177 15293281

[71] Li HF, Yang L, Yin D, Chen WJ, Liu GL, Ni W, Associations between neuroanatomical abnormality and motor symptoms in paroxysmal kinesigenic dyskinesia. Parkinsonism and Relat Disord (2019) 62:134–40. doi:10.1016/j.parkreldis.2018.12.029 30635245

[72] Ekmen A, Meneret A, Valabregue R, Beranger B, Worbe Y, Lamy JC, Cerebellum dysfunction in patients with *PRRT2* -Related paroxysmal Dyskinesia. Neurology (2022) 98(10):e1077–e1089. doi:10.1212/WNL.0000000000200060 35058336

[73] Draganski B, Schneider SA, Fiorio M, Klöppel S, Gambarin M, Tinazzi M, Genotype–phenotype interactions in primary dystonias revealed by differential changes in brain structure. NeuroImage. (2009) 47(4):1141–7. doi:10.1016/j.neuroimage.2009.03.057 19344776 PMC2741581

[74] Bradley D, Whelan R, Walsh R, Reilly RB, Hutchinson S, Molloy F, Temporal discrimination threshold: VBM evidence for an endophenotype in adult onset primary torsion dystonia. Brain (2009) 132(9):2327–35. doi:10.1093/brain/awp156 19525326

[75] Black KJ, Ȯṅgur D, Perlmutter JS. Putamen volume in idiopathic focal dystonia. Neurology (1998) 51(3):819–24. doi:10.1212/WNL.51.3.819 9748033

[76] Vo A, Sako W, Niethammer M, Carbon M, Bressman SB, Uluğ AM, Thalamocortical connectivity correlates with phenotypic variability in Dystonia. Cereb Cortex (2015) 25(9):3086–94. doi:10.1093/cercor/bhu104 24860017 PMC4537447

[77] Vidailhet M, Yelnik J, Lagrange C, Fraix V, Grabli D, Thobois S, Bilateral pallidal deep brain stimulation for the treatment of patients with dystonia-choreoathetosis cerebral palsy: a prospective pilot study. The Lancet Neurol (2009) 8(8):709–17. doi:10.1016/S1474-4422(09)70151-6 19576854

[78] Elias GJB, Boutet A, Joel SE, Germann J, Gwun D, Neudorfer C, Probabilistic mapping of deep brain stimulation: insights from 15 years of therapy. Ann Neurol (2021) 89(3):426–43. doi:10.1002/ana.25975 33252146

[79] Raghu ALB, Eraifej J, Sarangmat N, Stein J, FitzGerald JJ, Payne S, Pallido-putaminal connectivity predicts outcomes of deep brain stimulation for cervical dystonia. Brain (2021) 144(12):3589–96. doi:10.1093/brain/awab280 34293093 PMC8719844

[80] Coenen VA, Allert N, Paus S, Kronenbürger M, Urbach H, Mädler B. Modulation of the cerebello-thalamo-cortical network in thalamic deep brain stimulation for tremor. Neurosurgery (2014) 75(6):657–70. doi:10.1227/NEU.0000000000000540 25161000

[81] Valeriani D, Simonyan K. A microstructural neural network biomarker for dystonia diagnosis identified by a DystoniaNet deep learning platform. Proc Natl Acad Sci (2020) 117(42):26398–405. doi:10.1073/pnas.2009165117 33004625 PMC7586425

[82] Burciu RG, Hess CW, Coombes SA, Ofori E, Shukla P, Chung JW, Functional activity of the sensorimotor cortex and cerebellum relates to cervical dystonia symptoms. Hum Brain Mapp (2017) 38(9):4563–73. doi:10.1002/hbm.23684 28594097 PMC5547035

[83] Merchant SHI, Frangos E, Parker J, Bradson M, Wu T, Vial-Undurraga F, The role of the inferior parietal lobule in writer’s cramp. Brain (2020) 143(6): 1766–79. doi:10.1093/brain/awaa138 32428227 PMC7296854

[84] Ito C, Al-Hassany L, Kurth T, Glatz T. Distinguishing description, prediction, and causal inference. Neurology (2025) 104(4):e210171. doi:10.1212/WNL.0000000000210171 39899793

[85] Palombo M, Shemesh N, Ronen I, Valette J. Insights into brain microstructure from *in vivo* DW-MRS. NeuroImage (2018) 182:97–116. doi:10.1016/j.neuroimage.2017.11.028 29155183

[86] Marques Paulo AJ, Sato JR, de Faria DD, Balardin J, Borges V, de Azevedo Silva SM, Task-related brain activity in upper limb dystonia revealed by simultaneous fNIRS and EEG. Clin Neurophysiol (2024) 159:1–12. doi:10.1016/j.clinph.2023.12.008 38232654

[87] Patel AB, Lai JCK, Chowdhury GMI, Hyder F, Rothman DL, Shulman RG, Direct evidence for activity-dependent glucose phosphorylation in neurons with implications for the astrocyte-to-neuron lactate shuttle. Proc Natl Acad Sci (2014) 111(14):5385–90. doi:10.1073/pnas.1403576111 24706914 PMC3986127

[88] Stoessl AJ. Glucose utilization: still in the synapse. Nat Neurosci (2017) 20(3): 382–4. doi:10.1038/nn.4513 28230843

[89] Eidelberg D Metabolic brain networks in neurodegenerative disorders: a functional imaging approach. Trends Neurosciences (2009) 32(10):548–57. doi:10.1016/j.tins.2009.06.003 PMC2782537 19765835

[90] Carbon M, Eidelberg D. Abnormal structure-function relationships in hereditary dystonia. Neuroscience (2009) 164(1):220–9. doi:10.1016/j.neuroscience.2008.12.041 19162138 PMC2760608

[91] Niethammer M, Carbon M, Argyelan M, Eidelberg D. Hereditary dystonia as a neurodevelopmental circuit disorder: evidence from neuroimaging. Neurobiol Dis (2011) 42(2):202–9. doi:10.1016/j.nbd.2010.10.010 20965251 PMC3062649

[92] Vogelnik Žakelj K, Trošt M, Tomše P, Petrović IN, Tomić Pešić A, Radovanović S, Zolpidem improves task-specific dystonia: a randomized clinical trial integrating exploratory transcranial magnetic stimulation and [18F] FDG-PET imaging. Parkinsonism and Relat Disord (2024) 124:107014. doi:10.1016/j.parkreldis.2024.107014 38823169

[93] Tempel LW, Perlmutter JS. Abnormal cortical responses in patients with writer’s cramp. Neurology (1993) 43:2252–7. doi:10.1212/wnl.43.11.2252 8232938

[94] Feiwell RJ, Black KJ, McGee-Minnich LA, Synder AZ, MacLeod AM, Perlmutter KS. Diminished regional cerebral blood flow response to vibration in patients with blepharospasm. Neurology (1999) 52(2):291–7. doi:10.1212/wnl.52.2.291 9932946

[95] Todd RD, Carl J, Harmon S, O’Malley KL, Perlmutter JS. Dynamic changes in striatal dopamine D2 and D3 receptor protein and mRNA in response to 1-methyl-4-phenyl-1,2,3,6-tetrahydropyridine (MPTP) denervation in baboons. J Neurosci (1996) 16(23):7776–82.doi:10.1523/JNEUROSCI.16-23-07776.1996 8922433 PMC6579075

[96] Black KJ, Snyder AZ, Mink JW, Tolia VN, Revilla FJ, Moerlein SM, Spatial reorganization of putaminal dopamine D2-like receptors in cranial and hand dystonia. PLoS One (2014) 9(2):e88121. doi:10.1371/journal.pone.0088121 24520350 PMC3919754

[97] Asanuma K, Ma Y, Okulski J, Dhawan V, Chaly T, Carbon M, Decreased striatal D2 receptor binding in non-manifesting carriers of the DYT1 dystonia mutation. Neurology (2005) 64(2):347–9. doi:10.1212/01.WNL.0000149764.34953.BF 15668438

[98] Berman BD, Hallett M, Herscovitch P, Simonyan K. Striatal dopaminergic dysfunction at rest and during task performance in writer’s cramp. Brain (2013) 136(12):3645–58. doi:10.1093/brain/awt282 24148273 PMC3859223

[99] Simonyan K, Berman BD, Herscovitch P, Hallett M. Abnormal striatal dopaminergic neurotransmission during rest and task production in Spasmodic dysphonia. J Neurosci (2013) 33(37):14705–14. doi:10.1523/JNEUROSCI.0407-13.2013 24027271 PMC3771037

[100] Karimi M, Moerlein SM, Videen TO, Luedtke RR, Taylor M, Mach RH, Decreased striatal dopamine receptor binding in primary focal dystonia: a D2 or D3 defect? Mov Disord (2011) 26(1):100–6. doi:10.1002/mds.23401 20960437 PMC3025272

[101] Karimi MS, Moerlein SM, Videen TO, Su Y, Flores HP, Perlmutter JS. Striatal dopamine D1-like receptor binding is unchanged in primary focal dystonia. Mov Disord (2013) 28(14):2002–6. doi:10.1002/mds.25720 24151192 PMC4086787

[102] Simonyan K, Cho H, Sichani AH, Rubien-Thomas E, Hallett M. The direct basal ganglia pathway is hyperfunctional in focal dystonia. Brain (2017) 140(12): 3179–90. doi:10.1093/brain/awx263 29087445 PMC5841143

[103] Garibotto V, Romito LM, Elia AE, Soliveri P, Panzacchi A, Carpinelli A, *In vivo* evidence for GABA(A) receptor changes in the sensorimotor system in primary dystonia. Mov Disord (2011) 26(5):852–7. doi:10.1002/mds.23553 21370265

[104] Berman BD, Pollard RT, Shelton E, Karki R, Smith-Jones PM, Miao Y. GABAA receptor availability changes underlie symptoms in isolated cervical dystonia. Front Neurol (2018) 9:188. doi:10.3389/fneur.2018.00188 29670567 PMC5893646

[105] Gallea C, Herath P, Voon V, Lerner A, Ostuni K, Saad Z, Loss of inhibition in sensorimotor networks in focal hand dystonia. Neuroimage Clin (2018) 17:90–7. doi:10.1016/j.nicl.2017.10.011 29062685 PMC5645005

[106] Petrou M, Frey KA, Kilbourn MR, Scott PJ, Raffel DM, Bohnen NI, *In vivo* imaging of human cholinergic nerve terminals with (−)-5-(18)F-fluoroethoxybenzovesamicol: biodistribution, dosimetry, and tracer kinetic analyses. J Nucl Med (2014) 55(3):396–404.doi:10.2967/jnumed.113.124792 24481024

[107] Mazere J, Dilharreguy B, Catheline G, Vidailhet M, Deffains M, Vimont D, Striatal and cerebellar vesicular acetylcholine transporter expression is disrupted in human DYT1 dystonia. Brain (2021) 144(3):909–23. doi:10.1093/brain/awaa465 33638639

[108] Assous M Striatal cholinergic transmission. Focus on nicotinic receptors’ influence in striatal circuits. Eur J Neurosci (2021) 53(8):2421–42. doi:10.1111/ejn.15135 33529401 PMC8161166

[109] Ding JB, Guzman JN, Peterson JD, Goldberg JA, Surmeier DJ. Thalamic gating of corticostriatal signaling by cholinergic interneurons. Neuron (2010) 67(2): 294–307.doi:10.1016/j.neuron.2010.06.017 20670836 PMC4085694

[110] Peterson DA, Sejnowski TJ, Poizner H. Convergent evidence for abnormal striatal synaptic plasticity in dystonia. Neurobiol Dis (2010) 37(3):558–73. doi:10.1016/j.nbd.2009.12.003 20005952 PMC2846420

[111] Peterson DA, Sejnowski TJ. A dynamic circuit hypothesis for the pathogenesis of blepharospasm. Front Comput Neurosci (2017) 11:11. doi:10.3389/fncom.2017.00011 28326032 PMC5340098

[112] Beck S, Schubert M, Richardson SP, Hallett M. Surround inhibition depends on the force exerted and is abnormal in focal hand dystonia. J Appl Physiol (2009) 107(5):1513–8. doi:10.1152/japplphysiol.91580.2008 19713426 PMC2777802

[113] Bostan AC, Dum RP, Strick PL. The basal ganglia communicate with the cerebellum. Proc.Natl.Acad.Sci.U.S.A. (2010) 107(18):8452–6. doi:10.1073/pnas.1000496107 20404184 PMC2889518

[114] Hacker CD, Perlmutter JS, Criswell SR, Ances BM, Synder AZ. Resting state functional connectivity of the striatum in Parkinson’s disease. Brain (2012) 135(12): 3699–711. doi:10.1093/brain/aws281 23195207 PMC3525055

[115] Maiti B, Koller JM, Snyder AZ, Tanenbaum AB, Noris SA, Campbell MC, Cognitive correlates of cerebellar resting-state functional connectivity in Parkinson disease. Neurology (2020) 94(4):e384–e396. doi:10.1212/WNL.0000000000008754 31848257 PMC7079688

[116] Maiti B, Rawson KS, Tanenbaum AB, Koller JM, Snyder AZ, Campbell MC, Functional connectivity of vermis correlates with future gait impairments in parkinson’s disease. Mov Disord (2021) 36(11):2559–68. doi:10.1002/mds.28684 34109682 PMC8595492

[117] Norris SA, Morris AE, Campbell MC, Karimi M, Adeyemo B, Paniello RC, Regional, not global, functional connectivity contributes to isolated focal dystonia. Neurology (2020) 95(16):e2246–e2258. doi:10.1212/WNL.0000000000010791 32913023 PMC7713779

[118] Battistella G, Fuertinger S, Fleysher L, Ozelius LJ, Simonyan K. Cortical sensorimotor alterations classify clinical phenotype and putative genotype of Spasmodic dysphonia. Eur J Neurol (2016) 23(10):1517–27. doi:10.1111/ene.13067 27346568 PMC5308055

[119] Delnooz CCS, Helmich RC, Toni I, van de Warrenburg BPC. Reduced parietal connectivity with a premotor writing area in writer’s cramp. Movement Disord (2012) 27(11):1425–31. doi:10.1002/mds.25029 22886735

[120] Dresel C, Li Y, Wilzeck V, Castrop F, Zimmer C, Haslinger B. Multiple changes of functional connectivity between sensorimotor areas in focal hand dystonia. J Neurol Neurosurg and Psychiatry (2014) 85(11):1245–52. doi:10.1136/jnnp-2013-307127 24706945

[121] Zito GA, Tarrano C, Jegatheesan P, Ekmen A, Béranger B, Rebsamen M, Somatotopy of cervical dystonia in motor-cerebellar networks: evidence from resting state fMRI. Parkinsonism and Relat Disord (2022) 94:30–6. doi:10.1016/j.parkreldis.2021.11.034 34875561

[122] Premi E, Diano M, Gazzina S, Cauda F, Gualeni V, Tinazzi M, Functional connectivity networks in asymptomatic and symptomatic *DYT1* carriers. Movement Disord (2016) 31(11):1739–43. doi:10.1002/mds.26725 27453152

[123] Marapin RS, van der Horn HJ, van der Stouwe AMM, Dalenberg JR, de Jong BM, Tijssen MAJ. Altered brain connectivity in hyperkinetic movement disorders: a review of resting-state fMRI. NeuroImage: Clin (2023) 37:103302. doi:10.1016/j.nicl.2022.103302 36669351 PMC9868884

[124] Li Z, Prudente CN, Stilla R, Sathian K, Jinnah HA, Hu X. Alterations of resting-state fMRI measurements in individuals with cervical dystonia. Hum Brain Mapp (2017) 38(8):4098–108. doi:10.1002/hbm.23651 28504361 PMC5553075

[125] Sarasso E, Agosta F, Piramide N, Bianchi F, Butera C, Gatti R, Sensory trick phenomenon in cervical dystonia: a functional MRI study. J Neurol (2020) 267(4):1103–15. doi:10.1007/s00415-019-09683-5 31897600

[126] Hok P, Hvizdošová L, Otruba P, Kaiserová M, Trnečková M, Tüdös Z, Botulinum toxin injection changes resting state cerebellar connectivity in cervical dystonia. Scientific Rep (2021) 11(1):8322. doi:10.1038/s41598-021-87088-z PMC8050264 33859210

[127] Sarasso E, Emedoli D, Gardoni A, Zenere L, Canu E, Basaia S, Cervical motion alterations and brain functional connectivity in cervical dystonia. Parkinsonism and Relat Disord (2024) 120:106015. doi:10.1016/j.parkreldis.2024.106015 38325256

[128] Loh A, Elias GJB, Germann J, Boutet A, Gwun D, Yamamoto K, Neural correlates of optimal deep brain stimulation for cervical dystonia. Ann Neurol (2022) 92(3):418–24. doi:10.1002/ana.26450 35785489

[129] Glickman A, Nguyen P, Shelton E, Peterson DA, Berman BD. Basal ganglia and cerebellar circuits have distinct roles in blepharospasm. Parkinsonism and Relat Disord (2020) 78:158–64. doi:10.1016/j.parkreldis.2020.06.034 32891945

[130] Nelson AJ, Blake DT, Chen R. Digit-specific aberrations in the primary somatosensory cortex in Writer’s cramp. Ann Neurol (2009) 66(2):146–54. doi:10.1002/ana.21626 19743446

[131] Mohammadi B, Kollewe K, Samii A, Beckmann CF, Dengler R, Münte TF. Changes in resting-state brain networks in writer’s cramp. Hum Brain Mapp (2012) 33(4):840–8. doi:10.1002/hbm.21250 21484954 PMC6870480

[132] Kita K, Rokicki J, Furuya S, Sakamoto T, Hanakawa T. Resting-state basal ganglia network codes a motor musical skill and its disruption from dystonia. Movement Disord (2018) 33(9):1472–80. doi:10.1002/mds.27448 30277603 PMC6220822

[133] Alpheis S, Sinke C, Burek J, Krüger THC, Altenmüller E, Scholz DS. Increased functional connectivity of motor regions and dorsolateral prefrontal cortex in musicians with focal hand dystonia. J Neurol (2025) 272(4):281. doi:10.1007/s00415-025-13018-y 40119933 PMC11929630

[134] de Lima Xavier L, Simonyan K. The extrinsic risk and its association with neural alterations in Spasmodic dysphonia. Parkinsonism and Relat Disord (2019) 65:117–23.doi:10.1016/j.parkreldis.2019.05.034 PMC6774802 31153765

[135] Battistella G, Simonyan K. Top-down alteration of functional connectivity within the sensorimotor network in focal dystonia. Neurology (2019) 92(16): e1843–e1851. doi:10.1212/WNL.0000000000007317 30918091 PMC6550502

[136] Hanekamp S, Simonyan K. The large-scale structural connectome of task-specific focal dystonia. Hum Brain Mapp (2020) 41(12):3253–65. doi:10.1002/hbm.25012 32311207 PMC7375103

[137] Haslinger B, Noé J, Altenmüller E, Riedl V, Zimmer C, Mantel T, Changes in resting-state connectivity in musicians with embouchure dystonia. Movement Disord (2017) 32(3):450–8.doi:10.1002/mds.26893 27911020

[138] Mantel T, Meindl T, Li Y, Jochim A, Gora-Stahlberg G, Kräenbring J, Network-specific resting-state connectivity changes in the premotor-parietal axis in writer’s cramp. NeuroImage: Clin (2018) 17:137–44. doi:10.1016/j.nicl.2017.10.001 29085775 PMC5650679

[139] Huang XF, Zhu MR, Shan P, Pei CH, Liang ZH, Zhou HL, Multiple neural networks malfunction in primary blepharospasm: an independent components analysis. Front Hum Neurosci (2017) 11:235. doi:10.3389/fnhum.2017.00235 28539879 PMC5423973

[140] Rothkirch I, Granert O, Knutzen A, Wolff S, Gövert F, Pedersen A, Dynamic causal modeling revealed dysfunctional effective connectivity in both, the cortico-basal-ganglia and the cerebello-cortical motor network in writers’ cramp. NeuroImage: Clin (2018) 18:149–59.doi:10.1016/j.nicl.2018.01.015 29868443 PMC5984595

[141] Battistella G, Termsarasab P, Ramdhani RA, Fuertinger S, Simonyan K. Isolated focal dystonia as a disorder of large-scale functional networks. Cereb Cortex (2015) 27:bhv313–1215.doi:10.1093/cercor/bhv313 PMC6075177 26679193

[142] Jankowski J, Paus S, Scheef L, Bewersdorff M, Schild HH, Klockgether T, Abnormal movement preparation in task-specific focal hand dystonia. PLoS ONE (2013) 8(10):e78234.doi:10.1371/journal.pone.0078234 24167610 PMC3805688

[143] Castrop F, Dresel C, Hennenlotter A, Zimmer C, Haslinger B. Basal ganglia-premotor dysfunction during movement imagination in writer’s cramp. Movement Disord (2012) 27(11):1432–9.doi:10.1002/mds.24944 22328061

[144] Delnooz CCS, Helmich RC, Medendorp WP, de Warrenburg BPC, Toni I. Writer’s cramp:increased dorsal premotor activity during intended writing. Hum Brain Mapp (2013) 34(3):613–25. doi:10.1002/hbm.21464 22113948 PMC6870150

[145] de Vries PM, Johnson KA, de Jong BM, Gieteling EW, Bohning DE, George MS, Changed patterns of cerebral activation related to clinically normal hand movement in cervical dystonia. Clin Neurol Neurosurg (2008) 110(2):120–8. doi:10.1016/j.clineuro.2007.09.020 18006221

[146] Gallea C, Horovitz SG, Najee-Ullah M, Hallett M, Hallett M. Impairment of a parieto-premotor network specialized for handwriting in writer’s cramp. Hum Brain Mapp (2016) 37(12):4363–75.doi:10.1002/hbm.23315 27466043 PMC5217828

[147] Lerner A, Shill H, Hanakawa T, Bushara K, Goldfine A, Hallett M. Regional cerebral blood flow correlates of the severity of writer’s cramp symptoms. NeuroImage (2004) 21(3):904–13.doi:10.1016/j.neuroimage.2003.10.019 15006657

[148] Simonyan K, Ludlow CL. Abnormal activation of the primary somatosensory cortex in Spasmodic Dysphonia: an fMRI study. Cereb Cortex (2010) 20(11): 2749–59.doi:10.1093/cercor/bhq023 20194686 PMC2951850

[149] Islam T, Kupsch A, Bruhn H, Scheurig C, Schmidt S, Hoffmann KT. Decreased bilateral cortical representation patterns in writer’s cramp: a functional magnetic resonance imaging study at 3.0 T. Neurol Sci (2009) 30(3): 219–26. doi:10.1007/s10072-009-0045-7 19277833

[150] Kuo YL, Chen M, Kimberley TJ. Probing the inhibitory motor circuits in adductor laryngeal dystonia during a dystonia-unrelated task. Parkinsonism and Relat Disord (2023) 115:105812.doi:10.1016/j.parkreldis.2023.105812 PMC10592018 37651926

[151] Haslinger B, Altenmüller E, Castrop F, Zimmer C, Dresel C. Sensorimotor overactivity as a pathophysiologic trait of embouchure dystonia. Neurology (2010) 74(22):1790–7. doi:10.1212/WNL.0b013e3181e0f784 20513815

[152] Beukers RJ, Foncke EMJ, van der Meer JN, Nederveen AJ, de Ruiter MB, Bour LJ, Disorganized sensorimotor integration in mutation-positive myoclonus-dystonia. Arch Neurol (2010) 67(4):469–74. doi:10.1001/archneurol.2010.54 20385914

[153] Obermann M, Yaldizli O, Greiff AD, Konczak J, Lachenmayer ML, Tumczak F, Increased basal-ganglia activation performing a non-dystonia-related task in focal dystonia. Eur J Neurol (2008) 15(8):831–8. doi:10.1111/j.1468-1331.2008.02196.x 18557921

[154] Filip P, Gallea C, Lehéricy S, Bertasi E, Popa T, Mareček R, Disruption in cerebellar and basal ganglia networks during a visuospatial task in cervical dystonia. Movement Disord (2017) 32(5):757–68. doi:10.1002/mds.26930 28186664

[155] Zeuner KE, Knutzen A, Granert O, Götz J, Wolff S, Jansen O, Increased volume and impaired function: the role of the basal ganglia in writer’s cramp. Brain Behav (2015) 5(2):e00301. doi:10.1002/brb3.301 25642386 PMC4309880

[156] Wu CC, Fairhall SL, McNair NA, Hamm JP, Kirk IJ, Cunnington R, Impaired sensorimotor integration in focal hand dystonia patients in the absence of symptoms. J Neurol Neurosurg and Psychiatry (2010) 81(6):659–65. doi:10.1136/jnnp.2009.185637 19965853

[157] Kuo YL, Chen M, Huynh BP, Kimberley TJ. Task-dependency of the cerebellar-motor network in adductor laryngeal dystonia. Parkinsonism and Relat Disord (2024) 125:107038. doi:10.1016/j.parkreldis.2024.107038 PMC12885225 38896975

[158] Moore RD, Gallea C, Horovitz SG, Hallett M. Individuated finger control in focal hand dystonia: an fMRI study. NeuroImage (2012) 61(4):823–31. doi:10.1016/j.neuroimage.2012.03.066 22484405 PMC3376234

[159] Berman BD, Groth CL, Shelton E, Sillau SH, Sutton B, Legget KT, Hemodynamic responses are abnormal in isolated cervical dystonia. J Neurosci Res (2020) 98(4):692–703. doi:10.1002/jnr.24547 31692015 PMC7015799

[160] Schill J, Zeuner KE, Knutzen A, Tödt I, Simonyan K, Witt K. Functional neural networks in writer’s cramp as determined by graph-theoretical analysis. Front Neurol (2021) 12:744503. doi:10.3389/fneur.2021.744503 34887826 PMC8650489

[161] DeSimone JC, Archer DB, Vaillancourt DE, Wagle Shukla A. Network-level connectivity is a critical feature distinguishing dystonic tremor and essential tremor. Brain (2019) 142(6):1644–59. doi:10.1093/brain/awz085 30957839 PMC6536846

[162] O’Flynn LC, Simonyan K. Short- and long-term central action of botulinum neurotoxin treatment in laryngeal dystonia. Neurology (2022) 99(11):e1178–e1190.doi:10.1212/WNL.0000000000200850 35764404 PMC9536744

[163] Kita K, Furuya S, Osu R, Sakamoto T, Hanakawa T. Aberrant cerebello-cortical connectivity in pianists with focal task-specific dystonia. Cereb Cortex (2021) 31(10):4853–63. doi:10.1093/cercor/bhab127 34013319

[164] Khosravani S, Chen G, Ozelius LJ, Simonyan K. Neural endophenotypes and predictors of laryngeal dystonia penetrance and manifestation. Neurobiol Dis (2021) 148:105223. doi:10.1016/j.nbd.2020.105223 33316367 PMC8284879

[165] Zeuner KE, Knutzen A, Granert O, Sablowsky S, Götz J, Wolff S, Altered brain activation in a reversal learning task unmasks adaptive changes in cognitive control in writer’s cramp. NeuroImage: Clin (2016) 10:63–70. doi:10.1016/j.nicl.2015.11.006 26702397 PMC4669532

[166] Tarrano C, Galléa C, Delorme C, McGovern EM, Atkinson-Clement C, Barnham IJ, Association of abnormal explicit sense of agency with cerebellar impairment in myoclonus-dystonia. Brain Commun (2024) 6:fcae105. doi:10.1093/braincomms/fcae105 38601915 PMC11004927

[167] Uehara K, Furuya S, Numazawa H, Kita K, Sakamoto T, Hanakawa T. Distinct roles of brain activity and somatotopic representation in pathophysiology of focal dystonia. Hum Brain Mapp (2019) 40(6):1738–49. doi:10.1002/hbm.24486 30570801 PMC6865568

[168] Dresel C, Bayer F, Castrop F, Rimpau C, Zimmer C, Haslinger B. Botulinum toxin modulates basal ganglia but not deficient somatosensory activation in orofacial dystonia. Movement Disord (2011) 26(8):1496–502. doi:10.1002/mds.23497 21604301

[169] Langbour N, Michel V, Dilharreguy B, Guehl D, Allard M, Burbaud P. The cortical processing of sensorimotor sequences is disrupted in writer’s cramp. Cereb Cortex (2016) 27:bhw108–2559. doi:10.1093/cercor/bhw108 27114174

[170] Maguire F, Reilly RB, Simonyan K. Normal temporal discrimination in musician’s dystonia is linked to aberrant sensorimotor processing. Movement Disord (2020) 35(5):800–7. doi:10.1002/mds.27984 31930574 PMC7818836

[171] Mc Govern EM, Killian O, Narasimham S, Quinlivan B, Butler JB, Beck R, Disrupted superior collicular activity may reveal cervical dystonia disease pathomechanisms. Scientific Rep (2017) 7(1):16753. doi:10.1038/s41598-017-17074-x PMC5711841 29196716

[172] Chang EF, Turner RS, Ostrem JL, Davis VR, Starr PA. Neuronal responses to passive movement in the globus pallidus internus in primary dystonia. J Neurophysiol (2007) 98(6):3696–707. doi:10.1152/jn.00594.2007 17942626

[173] Peller M, Zeuner KE, Munchau A, Quartarone A, Weiss M, Knutzen A, The basal ganglia are hyperactive during the discrimination of tactile stimuli in writer’s cramp. Brain (2006) 129(10):2697–708. doi:10.1093/brain/awl181 16854945

[174] Obermann M, Vollrath C, de Greiff A, Gizewski ER, Diener H, Hallett M, Sensory disinhibition on passive movement in cervical dystonia. Movement Disord (2010) 25(15):2627–33. doi:10.1002/mds.23321 20725914

[175] Kimmich O, Molloy A, Whelan R, Williams L, Bradley D, Balsters J, Temporal discrimination, a cervical dystonia endophenotype: penetrance and functional correlates. Movement Disord (2014) 29(6):804–11. doi:10.1002/mds.25822 24482092

[176] Tödt I, Baumann A, Knutzen A, Granert O, Tzvi E, Lindert J, Abnormal effective connectivity in the sensory network in writer’s cramp. NeuroImage: Clin (2021) 31:102761. doi:10.1016/j.nicl.2021.102761 34298476 PMC8378794

[177] Schneider SA, Pleger B, Draganski B, Cordivari C, Rothwell JC, Bhatia KP, Modulatory effects of 5Hz rTMS over the primary somatosensory cortex in focal dystonia—An fMRI-TMS study. Movement Disord (2010) 25(1):76–83. doi:10.1002/mds.22825 20058321 PMC2929458

[178] Termsarasab P, Ramdhani RA, Battistella G, Rubien-Thomas E, Choy M, Farwell IM, Neural correlates of abnormal sensory discrimination in laryngeal dystonia. NeuroImage: Clin (2016) 10:18–26. doi:10.1016/j.nicl.2015.10.016 26693398 PMC4660380

[179] Frankford SA, O’Flynn LC, Simonyan K. Sensory processing in the auditory and olfactory domains is normal in laryngeal dystonia. J Neurol (2023) 270(4): 2184–90. doi:10.1007/s00415-023-11562-z 36640203 PMC10352682

[180] Wilkes BJ, Adury RZ, Berryman D, Concepcion LR, Liu Y, Yokoi F, Cell-specific Dyt1 ΔGAG knock-in to basal ganglia and cerebellum reveal differential effects on motor behavior and sensorimotor network function. Exp Neurol (2023) 367:114471. doi:10.1016/j.expneurol.2023.114471 37321386 PMC10695146

[181] Hervé D Identification of a specific assembly of the G protein golf as a critical and regulated module of dopamine and adenosine-activated cAMP pathways in the striatum. Front Neuroanat (2011) 5:48. doi:10.3389/fnana.2011.00048 21886607 PMC3155884

[182] Méneret A, Roze E. Paroxysmal movement disorders: an update. Revue Neurologique (2016) 172(8–9):433–45. doi:10.1016/j.neurol.2016.07.005 27567459

[183] Nieuwhof F, Toni I, Dirkx MF, Gallea C, Vidailhet M, Buijink AWG, Cerebello-thalamic activity drives an abnormal motor network into dystonic tremor. NeuroImage: Clin (2022) 33:102919. doi:10.1016/j.nicl.2021.102919 34929584 PMC8688717

[184] van der Salm SMA, van der Meer JN, Nederveen AJ, Veltman DJ, van Rootselaar AF, Tijssen MAJ. Functional MRI study of response inhibition in myoclonus dystonia. Exp Neurol (2013) 247:623–9. doi:10.1016/j.expneurol.2013.02.017 23474191

[185] Vo A, Sako W, Fujita K, Peng S, Mattis PJ, Skidmore FM, Parkinson’s disease-related network topographies characterized with resting state functional MRI. Hum Brain Mapp (2017) 38(2):617–30. doi:10.1002/hbm.23260 27207613 PMC5118197

[186] Schindlbeck KA, Vo A, Mattis PJ, Villringer K, Marzinzik F, Fiebach JB, Cognition-related functional topographies in parkinson’s disease: localized loss of the ventral default mode network. Cereb Cortex (2021) 31(11):5139–50. doi:10.1093/cercor/bhab148 34148072 PMC8491681

[187] Vo A, Nguyen N, Fujita K, Schindlbeck KA, Rommal A, Bressman SB, Disordered network structure and function in dystonia: pathological connectivity vs. adaptive responses. Cereb Cortex (2023) 33(11):6943–58. doi:10.1093/cercor/bhad012 36749014 PMC10233302

[188] Niethammer M, Tang CC, Vo A, Nguyen N, Spetsieris P, Dhawan V, Gene therapy reduces Parkinson’s disease symptoms by reorganizing functional brain connectivity. Sci Translational Med (2018) 10(469):eaau0713. doi:10.1126/scitranslmed.aau0713 30487248

[189] Schindlbeck KA, Vo A, Nguyen N, Tang CC, Niethammer M, Dhawan V, LRRK2 and GBA variants exert distinct influences on parkinson’s disease-specific metabolic networks. Cereb Cortex (2020) 30(5):2867–78. doi:10.1093/cercor/bhz280 31813991 PMC7197067

[190] Ko JH, Spetsieris PG, Eidelberg D. Network structure and function in parkinson’s disease. Cereb Cortex (2017) 28:1–15. doi:10.1093/cercor/bhx267 PMC6215468 29088324

[191] King M, Shahshahani L, Ivry RB, Diedrichsen J. A task-general connectivity model reveals variation in convergence of cortical inputs to functional regions of the cerebellum. eLife (2023) 12:e81511. doi:10.7554/eLife.81511 37083692 PMC10129326

[192] Rossini PM, Iorio RD, Bentivoglio M, Bertini G, Ferreri F, Gerloff C, Methods for analysis of brain connectivity: an IFCN-sponsored review. Clin Neurophysiol (2019) 130(10):1833–58. doi:10.1016/j.clinph.2019.06.006 31401492

[193] Bara-Jimenez W, Catalan MJ, Hallett M, Gerloff C. Abnormal somatosensory homunculus in dystonia of the hand. Ann Neurol (1998) 44(5):828–31. doi:10.1002/ana.410440520 9818942

[194] Toro C, Deuschl G, Hallett M. Movement-related electroencephalographic desynchronization in patients with hand cramps: evidence for motor cortical involvement in focal dystonia. Ann Neurol (2000) 47(4):456–61. doi:10.1002/1531-8249(200004)47:4456::AID-ANA83.0.CO;2-Q 10762156

[195] Jin S, Lin P, Auh S, Hallett M. Abnormal functional connectivity in focal hand dystonia: mutual information analysis in EEG. Movement Disord (2011) 26(7): 1274–81. doi:10.1002/mds.23675 21506166 PMC3119738

[196] Jin SH, Lin P, Hallett M. Reorganization of brain functional small-world networks during finger movements. Hum Brain Mapp (2012) 33(4):861–72. doi:10.1002/HBM.21253 21484955 PMC6870111

[197] Jin SH, Lin P, Hallett M. Abnormal reorganization of functional cortical small-world networks in focal hand dystonia. PLoS ONE (2011) 6(12):e28682. doi:10.1371/journal.pone.0028682 22174867 PMC3236757

[198] Melgari JM, Zappasodi F, Porcaro C, Tomasevic L, Cassetta E, Rossini PM, Movement-induced uncoupling of primary sensory and motor areas in focal task-specific hand dystonia. Neuroscience (2013) 250:434–45. doi:10.1016/j.neuroscience.2013.07.027 23876327

[199] Chen CC, Macerollo A, Heng HM, Lu MK, Tsai CH, Daniyal, Low-frequency oscillations in cortical level to help diagnose task-specific dystonia. Neurobiol Dis (2021) 157:105444. doi:10.1016/j.nbd.2021.105444 34265424

[200] Thirugnanasambandam N, Zimmerman T, Pillai AS, Shields J, Horovitz SG, Hallett M. Task-specific interhemispheric hypoconnectivity in writer’s cramp – an EEG study. Clin Neurophysiol (2020) 131(5):985–93. doi:10.1016/j.clinph.2020.01.011 32193164 PMC8214401

[201] Baltazar CA, Machado BS, de Faria DD, Paulo AJM, Silva SMCA, Ferraz HB, Brain connectivity in patients with dystonia during motor tasks. J Neural Eng (2020) 17(5):56039. doi:10.1088/1741-2552/abbbd6 32977316

[202] Ehrlich SK, Battistella G, Simonyan K. Temporal signature of task-specificity in isolated focal laryngeal dystonia. Movement Disord (2023) 38(10):1925–35. doi:10.1002/mds.29557 37489600 PMC10615685

[203] Kumar A, Lin CC, Kuo SH, Pan MK. Physiological recordings of the cerebellum in movement disorders. The Cerebellum (2022) 22(5):985–1001. doi:10.1007/s12311-022-01473-6 36070135 PMC10354710

[204] Honkanen EA, Rönkä J, Pekkonen E, Aaltonen J, Koivu M, Eskola O, GPi-DBS-induced brain metabolic activation in cervical dystonia. J Neurology, Neurosurgery, Psychiatry (2024) 95(4):300–8. doi:10.1136/jnnp-2023-331668 37758453

[205] Filip P, Jech R, Fečíková A, Havránková P, Růžička F, Mueller K, Restoration of functional network state towards more physiological condition as the correlate of clinical effects of pallidal deep brain stimulation in dystonia. Brain Stimulation (2022) 15(5):1269–78. doi:10.1016/j.brs.2022.08.025 36096443

[206] Butenko K, Neudorfer C, Dembek TA, Hollunder B, Meyer GM, Li N, Engaging dystonia networks with subthalamic stimulation. Proc Natl Acad Sci (2025) 122(2):e2417617122. doi:10.1073/pnas.2417617122 39773021 PMC11745339

[207] Horisawa S, Kohara K, Murakami M, Fukui A, Kawamata T, Taira T. Deep brain stimulation of the Forel’s field for dystonia: preliminary results. Front Hum Neurosci (2021) 15:768057. doi:10.3389/fnhum.2021.768057 34912201 PMC8667223

[208] Su Jhui, Wen HY, Yang Y, yu LR, Teng F, xi LL, Dystonia and the pedunculopontine nucleus: current evidences and potential mechanisms. Front Neurol (2022) 13:1065163. doi:10.3389/fneur.2022.1065163 36504662 PMC9727297

[209] Shih LC, Vanderhorst VG, Lozano AM, Hamani C, Moro E. Improvement of pisa syndrome with contralateral pedunculopontine stimulation. Movement Disord (2013) 28(4):555–6. doi:10.1002/mds.25301 23389993 PMC3622834

[210] Starr PA. Totally implantable bidirectional neural prostheses: a flexible platform for innovation in neuromodulation. Front Neurosci (2018) 12:619. doi:10.3389/fnins.2018.00619 30245616 PMC6137308

[211] Horn A The impact of modern-day neuroimaging on the field of deep brain stimulation. Curr Opin Neurol (2019) 32(4):511–20. doi:10.1097/WCO.0000000000000679 30844863

[212] Neumann W, Horn A, Ewert S, Huebl J, Brücke C, Slentz C, A localized pallidal physiomarker in cervical dystonia. Ann Neurol (2017) 82(6):912–24. doi:10.1002/ana.25095 29130551

[213] Hendrix CM, Vitek JL. Toward a network model of dystonia. Ann New York Acad Sci (2012) 1265(1):46–55. doi:10.1111/j.1749-6632.2012.06692.x 22823747

[214] Reich MM, Horn A, Lange F, Roothans J, Paschen S, Runge J, Probabilistic mapping of the antidystonic effect of pallidal neurostimulation: a multicentre imaging study. Brain (2019) 142(5):1386–98. doi:10.1093/brain/awz046 30851091

[215] Al-Fatly B, Ewert S, Kübler D, Kroneberg D, Horn A, Kühn AA. Connectivity profile of thalamic deep brain stimulation to effectively treat essential tremor. Brain (2019) 142(10):3086–98. doi:10.1093/brain/awz236 31377766

[216] Okromelidze L, Tsuboi T, Eisinger RS, Burns MR, Charbel M, Rana M, Functional and structural connectivity patterns associated with clinical outcomes in deep brain stimulation of the globus pallidus internus for generalized dystonia. Am J Neuroradiology (2020) 41(3):508–14. doi:10.3174/ajnr.A6429 PMC7077906 32054614

[217] Baldermann JC, Melzer C, Zapf A, Kohl S, Timmermann L, Tittgemeyer M, Connectivity profile predictive of effective deep brain stimulation in obsessive-compulsive disorder. Biol Psychiatry (2019) 85(9):735–43. doi:10.1016/j.biopsych.2018.12.019 30777287

[218] Li N, Baldermann JC, Kibleur A, Treu S, Akram H, Elias GJB, A unified connectomic target for deep brain stimulation in obsessive-compulsive disorder. Nat Commun (2020) 11(1):3364. doi:10.1038/s41467-020-16734-3 32620886 PMC7335093

[219] Horn A, Reich M, Vorwerk J, Li N, Wenzel G, Fang Q, Connectivity predicts deep brain stimulation outcome in Parkinson disease. Ann Neurol (2017) 82(1):67–78. doi:10.1002/ana.24974 28586141 PMC5880678

[220] Nieuwenhuys R, Voogd J, van Huijzen C. The human central nervous System. Springer Science (2013).

[221] Maier-Hein KH, Neher PF, Houde JC, Côté MA, Garyfallidis E, Zhong J, The challenge of mapping the human connectome based on diffusion tractography. Nat Commun (2017) 8(1):1349. doi:10.1038/s41467-017-01285-x 29116093 PMC5677006

[222] Petersen MV, Mlakar J, Haber SN, Parent M, Smith Y, Strick PL, Holographic reconstruction of axonal pathways in the human brain. Neuron (2019) 104(6):1056–64.e3. doi:10.1016/j.neuron.2019.09.030 31708306 PMC6948195

[223] Horn A, Reich MM, Ewert S, Li N, Al-Fatly B, Lange F, Optimal deep brain stimulation sites and networks for cervical vs. generalized dystonia. Proc Natl Acad Sci (2022) 119(14):e2114985119. doi:10.1073/pnas.2114985119 35357970 PMC9168456

[224] Horn A, Ewert S, Alho EJL, Axer M, Heinsen H, Fonoff ET, Teaching NeuroImages: *in vivo* visualization of edinger comb and Wilson pencils. Neurology (2019) 92(14):e1663–e1664. doi:10.1212/WNL.0000000000007252 30936236 PMC6448452

[225] Treu S, Strange B, Oxenford S, Neumann WJ, Kühn A, Li N, Deep brain stimulation: imaging on a group level. NeuroImage (2020) 219:117018. doi:10.1016/j.neuroimage.2020.117018 32505698

[226] Sanger TD, Liker M, Arguelles E, Deshpande R, Maskooki A, Ferman D, Pediatric deep brain stimulation using awake recording and stimulation for target selection in an inpatient neuromodulation monitoring unit. Brain Sci (2018) 8(7): 135. doi:10.3390/brainsci8070135 30018276 PMC6070881

[227] Liker MA, Sanger TD, MacLean JA, Nataraj J, Arguelles E, Krieger M, Stereotactic awake basal ganglia electrophysiological recording and stimulation (SABERS): a novel staged procedure for personalized targeting of deep brain stimulation in pediatric movement and neuropsychiatric disorders. J Child Neurol (2024) 39(1–2):33–44. doi:10.1177/08830738231224057 38409793

[228] Ichinohe N, Mori F, Shoumura K. A di-synaptic projection from the lateral cerebellar nucleus to the laterodorsal part of the striatum via the central lateral nucleus of the thalamus in the rat. Brain Res (2000) 880(1–2):191–7. doi:10.1016/S0006-8993(00)02744-X 11033006

[229] Aïssa HB, Sala RW, Georgescu Margarint EL, Frontera JL, Varani AP, Menardy F, Functional abnormalities in the cerebello-thalamic pathways in a mouse model of DYT25 dystonia. eLife (2022) 11:e79135. doi:10.7554/eLife.79135 35699413 PMC9197392

[230] Hernandez-Martin E, Arguelles E, Liker M, Robison A, Sanger TD. Increased movement-related signals in both basal ganglia and cerebellar output pathways in two children with dystonia. Front Neurol (2022) 13:989340. doi:10.3389/fneur.2022.989340 36158959 PMC9500435

[231] Hernandez-Martin E, Kasiri M, Abe S, MacLean J, Olaya J, Liker M, Globus pallidus internus activity increases during voluntary movement in children with dystonia. iScience (2023) 26(7):107066. doi:10.1016/j.isci.2023.107066 37389183 PMC10300218

[232] Hernandez-Martin E, Vidmark JSL, MacLean JA, Sanger TD. What is the effect of benzodiazepines on deep brain activity? A study in pediatric patients with dystonia. Front Neurol (2023) 14:1215572. doi:10.3389/fneur.2023.1215572 37638186 PMC10457157

[233] Hammond C, Ammari R, Bioulac B, Garcia L. Latest view on the mechanism of action of deep brain stimulation. Movement Disord (2008) 23(15):2111–21. doi:10.1002/mds.22120 18785230

[234] Wang DD, de Hemptinne C, Miocinovic S, Ostrem JL, Galifianakis NB, San Luciano M, Pallidal deep-brain stimulation disrupts pallidal beta oscillations and coherence with primary motor cortex in Parkinson’s disease. The J Neurosci (2018) 38(19):4556–68. doi:10.1523/JNEUROSCI.0431-18.2018 29661966 PMC5943981

[235] Feldmann LK, van Rheede JJ, Busch JL, Fleming JE, Mathiopoulou V, Denison T, Diurnal modulation of subthalamic beta oscillatory power in Parkinson’s disease patients during deep brain stimulation. Npj Parkinson’s Dis (2022) 8(1):88. doi:10.1038/s41531-022-00350-7 35804160 PMC9270436

[236] Gilron R, Little S, Perrone R, Wilt R, de Hemptinne C, Yaroshinsky MS, Long-term wireless streaming of neural recordings for circuit discovery and adaptive stimulation in individuals with Parkinson’s disease. Nat Biotechnol (2021) 39(9):1078–85. doi:10.1038/s41587-021-00897-5 33941932 PMC8434942

[237] Miocinovic S, Swann NC, de Hemptinne C, Miller A, Ostrem JL, Starr PA. Cortical gamma oscillations in isolated dystonia. Parkinsonism and Relat Disord (2018) 49:104–5. doi:10.1016/j.parkreldis.2018.01.017 29371063

[238] Rastogi A, Cash R, Dunlop K, Vesia M, Kucyi A, Ghahremani A, Modulation of cognitive cerebello-cerebral functional connectivity by lateral cerebellar continuous theta burst stimulation. NeuroImage. (2017) 158:48–57. doi:10.1016/j.neuroimage.2017.06.048 28669908

[239] Strafella AP, Paus T, Fraraccio M, Dagher A. Striatal dopamine release induced by repetitive transcranial magnetic stimulation of the human motor cortex. Brain (2003) 126(12):2609–15. doi:10.1093/brain/awg268 12937078

[240] Huang YZ, Edwards MJ, Rounis E, Bhatia KP, Rothwell JC. Theta burst stimulation of the human motor cortex. Neuron (2005) 45(2):201–6. doi:10.1016/j.neuron.2004.12.033 15664172

[241] Siebner HR, Tormos JM, Baumann AOC, Auer C, Catala MD, Conrad B, Low-frequency repetitive transcranial magnetic stimulation of the motor cortex in writer’s cramp. Neurology (1999) 52(3):529–37. doi:10.1212/WNL.52.3.529 10025782

[242] Havrankova P, Jech R, Walker ND, Operto G, Tauchmanova J, Vymazal J, Repetitive TMS of the somatosensory cortex improves writer’s cramp and enhances cortical activity. Neuro Endocrinology Letters (2010) 31(1):73–86. 20150883

[243] Murase N, Rothwell JC, Kaji R, Urushihara R, Nakamura K, Murayama N, Subthreshold low-frequency repetitive transcranial magnetic stimulation over the premotor cortex modulates writer’s cramp. Brain (2005) 128(1):104–15. doi:10.1093/brain/awh315 15483042

[244] Richardson SP, Tinaz S, Chen R. Repetitive transcranial magnetic stimulation in cervical dystonia: effect of site and repetition in a randomized pilot trial. PLOS ONE (2015) 10(4):e0124937. doi:10.1371/journal.pone.0124937 25923718 PMC4414555

[245] Koch G, Porcacchia P, Ponzo V, Carrillo F, Cáceres-Redondo MT, Brusa L, Effects of two weeks of cerebellar theta burst stimulation in cervical dystonia patients. Brain Stimulation (2014) 7(4):564–72. doi:10.1016/j.brs.2014.05.002 24881805

[246] Popa T, Hubsch C, James P, Richard A, Russo M, Pradeep S, Abnormal cerebellar processing of the neck proprioceptive information drives dysfunctions in cervical dystonia. Scientific Rep (2018) 8(1):2263. doi:10.1038/s41598-018-20510-1 PMC5797249 29396401

[247] Hubsch C, Roze E, Popa T, Russo M, Balachandran A, Pradeep S, Defective cerebellar control of cortical plasticity in writer’s cramp. Brain (2013) 136(7):2050–62. doi:10.1093/brain/awt147 23801734 PMC3692031

[248] Shukla AW, Hu W, Legacy J, Deeb W, Hallett M. Combined effects of rTMS and botulinum toxin therapy in benign essential blepharospasm. Brain Stimulation (2018) 11(3):645–7. doi:10.1016/j.brs.2018.02.004 29530449

[249] Hok P, Veverka T, Hluštík P, Nevrlý M, Kaňovský P. The central effects of botulinum toxin in Dystonia and spasticity. Toxins (2021) 13(2):155. doi:10.3390/toxins13020155 33671128 PMC7922085

[250] Wagle Shukla A Basis of movement control in dystonia and why botulinum toxin should influence it? Toxicon : Official Journal Int Soc Toxinology (2024) 237: 107251. doi:10.1016/j.toxicon.2023.107251 PMC13250885 37574115

[251] Cho HJ, Shin HW, Panyakaew P, Kassavetis P, Popa T, Wu T, Differential induction of Parieto-motor plasticity in writer’s cramp and cervical dystonia. Neurobiol Dis (2024) 202:106724. doi:10.1016/j.nbd.2024.106724 39491631 PMC11578765

[252] Quartarone A, Bagnato S, Rizzo V, Siebner HR, Dattola V, Scalfari A, Abnormal associative plasticity of the human motor cortex in writer’s cramp. Brain (2003) 126(12):2586–96. doi:10.1093/brain/awg273 14506068

[253] Quartarone A, Rizzo V, Bagnato S, Morgante F, Sant’Angelo A, Romano M, Homeostatic-like plasticity of the primary motor hand area is impaired in focal hand dystonia. Brain (2005) 128(8):1943–50. doi:10.1093/brain/awh527 15872016

[254] Sadnicka A, Hamada M, Bhatia KP, Rothwell JC, Edwards MJ. A reflection on plasticity research in writing dystonia. Movement Disord (2014) 29(8):980–7. doi:10.1002/mds.25908 24821685

[255] Benninger DH, Lomarev M, Lopez G, Pal N, Luckenbaugh DA, Hallett M. Transcranial direct current stimulation for the treatment of focal hand dystonia. Movement Disord (2011) 26(9):1698–702. doi:10.1002/mds.23691 21495074 PMC4180819

[256] Bradnam LV, Graetz LJ, McDonnell MN, Ridding MC. Anodal transcranial direct current stimulation to the cerebellum improves handwriting and cyclic drawing kinematics in focal hand dystonia. Front Hum Neurosci (2015) 9:286. doi:10.3389/fnhum.2015.00286 26042019 PMC4435234

[257] Furuya S, Nitsche MA, Paulus W, Altenmüller E. Surmounting retraining limits in musicians’ dystonia by transcranial stimulation. Ann Neurol (2014) 75(5): 700–7. doi:10.1002/ana.24151 24706370

[258] Rosset-Llobet J, Fàbregas-Molas S, Pascual-Leone Á. Effect of transcranial direct current stimulation on neurorehabilitation of task-specific dystonia: a double-blind, randomized clinical trial. Med Probl Performing Artists (2015) 30(3):178–84. doi:10.21091/mppa.2015.3033 26395620

[259] Nguyen JP, Suarez A, Malineau C, Dixneuf V, Mazaltarine G, Damier P. Treatment of cervical dystonia and blepharospasm by anodal tDCS of cerebellar hemispheres. A case report. Neurophysiologie Clinique (2020) 50(5):391–2. doi:10.1016/j.neucli.2020.09.006 33008674

[260] Angelakis E, Liouta E, Andreadis N, Leonardos A, Ktonas P, Stavrinou LC, Transcranial alternating current stimulation reduces symptoms in intractable idiopathic cervical dystonia: a case study. Neurosci Lett (2013) 533:39–43. doi:10.1016/j.neulet.2012.11.007 23149130

[261] Fomenko A, Chen KHS, Nankoo JF, Saravanamuttu J, Wang Y, El-Baba M, Systematic examination of low-intensity ultrasound parameters on human motor cortex excitability and behavior. eLife (2020) 9:e54497. doi:10.7554/eLife.54497 33236981 PMC7728443

[262] Verhagen L, Gallea C, Folloni D, Constans C, Jensen DEA, Ahnine H, Offline impact of transcranial focused ultrasound on cortical activation in primates. eLife (2019) 8:e40541. doi:10.7554/eLife.40541 30747105 PMC6372282

[263] Zeng K, Darmani G, Fomenko A, Xia X, Tran S, Nankoo J, Induction of human motor cortex plasticity by theta burst transcranial ultrasound stimulation. Ann Neurol (2022) 91(2):238–52. doi:10.1002/ana.26294 34964172

[264] Darmani G, Ramezanpour H, Sarica C, Annirood R, Grippe T, Nankoo JF, Individualized non-invasive deep brain stimulation of the basal ganglia using transcranial ultrasound stimulation. Nat Commun (2025) 16(1):2693. doi:10.1038/s41467-025-57883-7 40108143 PMC11923056

[265] Fox MD, Liu H, Pascual-Leone A. Identification of reproducible individualized targets for treatment of depression with TMS based on intrinsic connectivity. NeuroImage (2013) 66:151–60. doi:10.1016/j.neuroimage.2012.10.082 23142067 PMC3594474

[266] Santarnecchi E, Momi D, Sprugnoli G, Neri F, Pascual-Leone A, Rossi A, Modulation of network-to-network connectivity via spike-timing-dependent noninvasive brain stimulation. Hum Brain Mapp (2018) 39(12):4870–83. doi:10.1002/hbm.24329 30113111 PMC6866459

[267] Koch G Cortico-cortical connectivity: the road from basic neurophysiological interactions to therapeutic applications. Exp Brain Res (2020) 238(7–8):1677–84. doi:10.1007/s00221-020-05844-5 32705294

[268] Luber B, Davis SW, Deng ZD, Murphy D, Martella A, Peterchev AV, Using diffusion tensor imaging to effectively target TMS to deep brain structures. NeuroImage (2022) 249:118863. doi:10.1016/j.neuroimage.2021.118863 34974116 PMC8851689

[269] Mencarelli L, Menardi A, Neri F, Monti L, Ruffini G, Salvador R, Impact of network-targeted multichannel transcranial direct current stimulation on intrinsic and network-to-network functional connectivity. J Neurosci Res (2020) 98(10):1843–56. doi:10.1002/jnr.24690 32686203 PMC9094635

[270] Saturnino GB, Puonti O, Nielsen JD, Antonenko D, Madsen KH, Thielscher A. SimNIBS 2.1: a comprehensive pipeline for individualized electric field modelling for transcranial brain stimulation. In: Brain and human body modeling. Springer International Publishing (2019). p. 3–25. doi:10.1007/978-3-030-21293-3_1 31725247

[271] Thielscher A, Antunes A, Saturnino GB. Field modeling for transcranial magnetic stimulation: a useful tool to understand the physiological effects of TMS? In: 2015 37th annual international conference of the IEEE engineering in medicine and biology Society (EMBC). IEEE (2015). p. 222–5. doi:10.1109/EMBC.2015.7318340 26736240

[272] Tremblay S, Rogasch NC, Premoli I, Blumberger DM, Casarotto S, Chen R, Clinical utility and prospective of TMS–EEG. Clin Neurophysiol (2019) 130(5): 802–44. doi:10.1016/j.clinph.2019.01.001 30772238

[273] Casula EP, Pellicciari MC, Picazio S, Caltagirone C, Koch G. Spike-timing-dependent plasticity in the human dorso-lateral prefrontal cortex. NeuroImage (2016) 143:204–13. doi:10.1016/j.neuroimage.2016.08.060 27591116

[274] Jones AP, Choe J, Bryant NB, Robinson CSH, Ketz NA, Skorheim SW, Dose-dependent effects of closed-loop tACS delivered during slow-wave oscillations on memory consolidation. Front Neurosci (2018) 12:867. doi:10.3389/fnins.2018.00867 30538617 PMC6277682

[275] Ketz N, Jones AP, Bryant NB, Clark VP, Pilly PK. Closed-loop slow-wave tACS improves sleep-dependent long-term memory generalization by modulating endogenous oscillations. The J Neurosci (2018) 38(33):7314–26. doi:10.1523/JNEUROSCI.0273-18.2018 30037830 PMC6596034

[276] Morrison-Ham J, Clark GM, Ellis EG, Cerins A, Joutsa J, Enticott PG, (2021). Effects of non-invasive brain stimulation in dystonia: a systematic review and meta-analysis. medRxiv. doi:10.1101/2021.11.02.21265839 PMC9793065 36583118

[277] Porcacchia P, de Toledo PÁ, Rodríguez-Baena A, Martín-Rodríguez JF, Palomar FJ, Vargas-González L, Abnormal cerebellar connectivity and plasticity in isolated cervical dystonia. PLOS ONE (2019) 14(1):e0211367. doi:10.1371/journal.pone.0211367 30682155 PMC6347195

[278] Shaikh AG, Zee DS, Crawford JD, Jinnah HA. Cervical dystonia: a neural integrator disorder. Brain (2016) 139(10):2590–9. doi:10.1093/brain/aww141 27324878 PMC5840887

[279] Shadmehr R Distinct neural circuits for control of movement vs. holding still. J Neurophysiol (2017) 117(4):1431–60. doi:10.1152/jn.00840.2016 28053244 PMC5376603

[280] Klier EM, Wang H, Crawford JD. Interstitial nucleus of cajal encodes three-dimensional head orientations in fick-like coordinates. J Neurophysiol (2007) 97(1): 604–17. doi:10.1152/jn.00379.2006 17079347

[281] Leon PS, Knock SA, Woodman MM, Domide L, Mersmann J, McIntosh AR, The virtual brain: a simulator of primate brain network dynamics. Front Neuroinformatics (2013) 7:10. doi:10.3389/fninf.2013.00010 PMC3678125 23781198

[282] Rabuffo G, Lokossou HA, Li Z, Ziaee-Mehr A, Hashemi M, Quilichini PP, Mapping global brain reconfigurations following local targeted manipulations. Proc Natl Acad Sci (2025) 122(16):e2405706122. doi:10.1073/pnas.2405706122 40249780 PMC12037044

[283] Deco G, Jirsa VK, McIntosh AR. Emerging concepts for the dynamical organization of resting-state activity in the brain. Nat Rev Neurosci (2011) 12(1): 43–56. doi:10.1038/nrn2961 21170073

[284] Jirsa VK, Proix T, Perdikis D, Woodman MM, Wang H, Gonzalez-Martinez J, The virtual epileptic patient: individualized whole-brain models of epilepsy spread. NeuroImage (2017) 145:377–88. doi:10.1016/j.neuroimage.2016.04.049 27477535

[285] Clinicaltrials.gov. Improving EPilepsy surgery management and progNOsis using virtual epileptic patient software (VEP) - full text view - ClinicalTrials.gov. Available online at: https://clinicaltrials.gov/ct2/show/NCT03643016?id=NCT03643016&draw=2&rank=1&load=cart (Accessed January 13th, 2022).

[286] Zimmermann J, Perry A, Breakspear M, Schirner M, Sachdev P, Wen W, Differentiation of Alzheimer’s disease based on local and global parameters in personalized virtual brain models. NeuroImage: Clin (2018) 19:240–51. doi:10.1016/j.nicl.2018.04.017 30035018 PMC6051478

[287] Meier JM, Perdikis D, Blickensdörfer A, Stefanovski L, Liu Q, Maith O, Virtual deep brain stimulation: Multiscale co-simulation of a spiking basal ganglia model and a whole-brain mean-field model with the virtual brain. Exp Neurol (2022) 354:114111. doi:10.1016/j.expneurol.2022.114111 35569510

[288] Barabási DL, Bianconi G, Bullmore E, Burgess M, Chung S, Eliassi-Rad T, Neuroscience needs network science. The J Neurosci (2023) 43(34):5989–95. doi:10.1523/JNEUROSCI.1014-23.2023 37612141 PMC10451115

[289] Chen Y, Bukhari Q, Lin TW, Sejnowski TJ. Functional connectivity of fMRI using differential covariance predicts structural connectivity and behavioral reaction times. Netw Neurosci (2022) 6(2):614–33. doi:10.1162/netn_a_00239 35733425 PMC9207998

[290] Dawson J, Liu CY, Francisco GE, Cramer SC, Wolf SL, Dixit A, Vagus nerve stimulation paired with rehabilitation for upper limb motor function after ischaemic stroke (VNS-REHAB): a randomised, blinded, pivotal, device trial. The Lancet (2021) 397(10284):1545–53. doi:10.1016/S0140-6736(21)00475-X PMC8862193 33894832

[291] Bhattacharya A, Mrudula K, Sreepada SS, Sathyaprabha TN, Pal PK, Chen R, An overview of noninvasive brain stimulation: basic principles and clinical applications. Can J Neurol Sci/J Canadien des Sci Neurologiques (2021) 49:1–14. doi:10.1017/cjn.2021.158 34238393

[292] Mondal B, Choudhury S, Banerjee R, Roy A, Chatterjee K, Basu P, Non-invasive vagus nerve stimulation improves clinical and molecular biomarkers of Parkinson’s disease in patients with freezing of gait. Npj Parkinson’s Dis (2021) 7(1):46. doi:10.1038/s41531-021-00190-x 34045464 PMC8160211

[293] McClelland VM, Lin JP. Dystonia in childhood: how insights from paediatric research enrich the network theory of dystonia. Adv Neurobiol (2023) 31:1–22. doi:10.1007/978-3-031-26220-3_1 37338693

[294] Gonzalez-Latapi P, Marotta N, Mencacci NE. Emerging and converging molecular mechanisms in dystonia. J Neural Transm (2021) 128:483–98. doi:10.1007/s00702-020-02290-z 33386558

[295] Altenmüller E, Ioannou CI, Lee A. Apollo’s curse: neurological causes of motor impairments in musicians. In: Progress in brain research. Elsevier B.V. (2015).p. 89–106. doi:10.1016/bs.pbr.2014.11.022 25725911

[296] Richardson SP, Altenmüller E, Alter K, Alterman RL, Chen R, Frucht S, Research priorities in limb and task-specific dystonias. Front Neurol (2017) 18. doi:10.3389/fneur.2017.00170 PMC5413505 28515706

[297] Roze E, Soumaré A, Pironneau I, Sangla S, de Cock VC, Teixeira A, Case-control study of writer’s cramp. Brain (2009) 132(3):756–64. doi:10.1093/brain/awn363 19179376

[298] Sadnicka A, Kornysheva K, Rothwell JC, Edwards MJ. A unifying motor control framework for task-specific dystonia. Nat Rev Neurol (2018) 14(2):116–24. doi:10.1038/nrneurol.2017.146 29104291 PMC5975945

[299] Rosset-Llobet J, Fàbregas-Molas S. Rehabilitation and plasticity of task-specific focal hand dystonia. In: Treatment of Dystonia. Cambridge University Press (2018). p. 256–60. doi:10.1017/9781316459324.052

[300] Sadnicka A, Rosset-Llobet J. A motor control model of task-specific dystonia and its rehabilitation. Prog Brain Res (2019) 249:269–83. doi:10.1016/bs.pbr.2019.04.011 31325986

[301] Sadnicka A, Edwards MJ. Between nothing and everything: phenomenology in movement disorders. Movement Disord (2023) 38(10):1767–73. doi:10.1002/mds.29584 37735886

[302] Sanger TD. A computational model of deep-brain stimulation for acquired dystonia in children. Front Comput Neurosci (2018) 12:77. doi:10.3389/fncom.2018.00077 30294268 PMC6158364

[303] He S, Baig F, Mostofi A, Pogosyan A, Debarros J, Green AL, Closed-loop deep brain stimulation for essential tremor based on thalamic local field potentials. Movement Disord (2021) 36(4):863–73. doi:10.1002/mds.28513 33547859 PMC7610625

[304] Schreglmann SR, Wang D, Peach RL, Li J, Zhang X, Latorre A, Non-invasive suppression of essential tremor via phase-locked disruption of its temporal coherence. Nat Commun (2021) 12(1):363. doi:10.1038/s41467-020-20581-7 33441542 PMC7806740

[305] Pauw JD, der Velden KV, Meirte J, Daele UV, Truijen S, Cras P, The effectiveness of physiotherapy for cervical dystonia: a systematic literature review. J Neurol (2014) 261(10):1857–65. doi:10.1007/s00415-013-7220-8 24413637

[306] van den Dool J, Visser B, Koelman JH, Engelbert RH, Tijssen MA. Long-term specialized physical therapy in cervical dystonia: outcomes of a randomized controlled trial. Arch Phys Med Rehabil (2019) 100(8):1417–25. doi:10.1016/j.apmr.2019.01.013 30796919

[307] Shaikh AG, Beylergil SB, Scorr L, Kilic-Berkmen G, Freeman A, Klein C, Dystonia and tremor. Neurology (2021) 96(4):e563–e574. doi:10.1212/WNL.0000000000011049 33046615 PMC7905789

[308] Haubenberger D, Hallett M. Essential tremor. New Engl J Med (2018) 378(19):1802–10. doi:10.1056/NEJMcp1707928 29742376

[309] Shaikh AG, Jinnah HA. Interdisciplinary insights into tremor in dystonia: navigating clinical controversies, definitional challenges, and pathophysiological complexities. Parkinsonism and Relat Disord (2024) 122:106068. doi:10.1016/j.parkreldis.2024.106068 38548571

[310] Shaikh AG, Fasano A, Pandey S, Helmich RC, Albanese A, Vidailhet M, Challenges in describing tremor and dystonia. Neurology (2025) 104(2):e210209. doi:10.1212/WNL.0000000000210209 39724539 PMC12289376

[311] Panyakaew P, Jinnah HA, Shaikh AG. Clinical features, pathophysiology, treatment, and controversies of tremor in dystonia. J Neurol Sci (2022) 435:120199. doi:10.1016/j.jns.2022.120199 35259651 PMC9100855

[312] Kirke DN, Battistella G, Kumar V, Rubien-Thomas E, Choy M, Rumbach A, Neural correlates of dystonic tremor: a multimodal study of voice tremor in Spasmodic dysphonia. Brain Imaging Behav (2017) 11(1):166–75. doi:10.1007/s11682-016-9513-x 26843004 PMC4972702

[313] Semenova U, Medvednik R, Popov V, Jinnah HA, Shaikh AG, Sedov A. Neuronal activity of pallidal *versus* cerebellar receiving thalamus in patients with cervical dystonia. The Cerebellum (2021) 20(2):151–9. doi:10.1007/s12311-020-01194-8 33009654

[314] Sedov A, Dzhalagoniya I, Semenova U, Gamaleya A, Tomskiy A, Jinnah HA, Unraveling the neural signatures: distinct pallidal patterns in dystonia subtypes. Parkinsonism and Relat Disord (2025) 130:107207. doi:10.1016/j.parkreldis.2024.107207 39631221

[315] Sedov A, Usova S, Semenova U, Gamaleya A, Tomskiy A, Beylergil SB, Pallidal activity in cervical dystonia with and without head tremor. The Cerebellum (2020) 19(3):409–18. doi:10.1007/s12311-020-01119-5 32095996 PMC8327359

[316] Nieuwhof F, Toni I, Buijink AWG, van Rootselaar AF, van de Warrenburg BPC, Helmich RC. Phase-locked transcranial electrical brain stimulation for tremor suppression in dystonic tremor syndromes. Clin Neurophysiol (2022) 140:239–50. doi:10.1016/j.clinph.2022.03.020 35469732

[317] Avanzino L, Martino D, van de Warrenburg BPC, Schneider SA, Abbruzzese G, Defazio G, Cortical excitability is abnormal in patients with the “fixed dystonia” syndrome. Movement Disord (2008) 23(5):646–52. doi:10.1002/mds.21801 18175341

[318] Frucht L, Perez DL, Callahan J, MacLean J, Song PC, Sharma N, Functional dystonia: differentiation from primary dystonia and multidisciplinary treatments. Front Neurol (2021) 11:605262. doi:10.3389/fneur.2020.605262 33613415 PMC7894256

[319] Tomic A, Agosta F, Sarasso E, Petrovic I, Basaia S, Pesic D, Are there two different forms of functional dystonia? A multimodal brain structural MRI study. Mol Psychiatry (2020) 25(12):3350–9. doi:10.1038/s41380-018-0222-2 30120414

[320] Schrag AE, Mehta AR, Bhatia KP, Brown RJ, Frackowiak RSJ, Trimble MR, The functional neuroimaging correlates of psychogenic versus organic dystonia. Brain (2013) 136(3):770–81. doi:10.1093/brain/awt008 23436503 PMC3580272

[321] Maurer CW, LaFaver K, Ameli R, Epstein SA, Hallett M, Horovitz SG. Impaired self-agency in functional movement disorders. Neurology (2016) 87(6): 564–70. doi:10.1212/WNL.0000000000002940 27385746 PMC4977370

[322] Canu E, Agosta F, Tomic A, Sarasso E, Petrovic I, Piramide N, Breakdown of the affective-cognitive network in functional dystonia. Hum Brain Mapp (2020) 41(11):3059–76. doi:10.1002/hbm.24997 32243055 PMC7336141

[323] Espay AJ, Maloney T, Vannest J, Norris MM, Eliassen JC, Neefus E, Dysfunction in emotion processing underlies functional (psychogenic) dystonia. Movement Disord (2018) 33(1):136–45. doi:10.1002/mds.27217 29124784 PMC5767134

[324] Ramos VFML, Pillai AS, Lungu C, Ostrem J, Starr P, Hallett M. Intraoperative neurophysiology in deep brain surgery for psychogenic dystonia. Ann Clin Translational Neurol (2015) 2(6):707–10. doi:10.1002/acn3.206 PMC4479530 26125045

[325] Spagnolo PA, Parker J, Horovitz S, Hallett M. Corticolimbic modulation via intermittent theta burst stimulation as a novel treatment for functional movement disorder: a proof-of-concept study. Brain Sci (2021) 11(6):791. doi:10.3390/brainsci11060791 34203993 PMC8232716

[326] Foerster BR, Nascimento TD, DeBoer M, Bender MA, Rice IC, Truong DQ, Excitatory and inhibitory brain metabolites as targets of motor cortex tDCS therapy in functional dystonia. Arthritis and Rheumatol (2015) 67(2):576–81. doi:10.1002/art.38945 PMC4446981 25371383

